# Corporate restructuring and firm performance in Vietnam: The moderating role of digital transformation

**DOI:** 10.1371/journal.pone.0303491

**Published:** 2024-05-20

**Authors:** Duc Hong Vo, Anh The Vo, Co Thi-Huyen Dinh, Ngoc Phu Tran

**Affiliations:** The CBER – Research Centre in Business, Economics & Resources, Ho Chi Minh City Open University, Ho Chi Minh City, Vietnam; University of Rome Tor Vergata: Universita degli Studi di Roma Tor Vergata, ITALY

## Abstract

In the digital age, firms should continually innovate and adapt to remain competitive and enhance performance. Innovation and adaptation require firms to take a holistic approach to their corporate structuring to ensure efficiency and effectiveness to stay competitive. This study examines how corporate restructuring impacts firm performance in Vietnam. We then investigate the moderating role of digital transformation in the corporate restructuring–firm performance nexus. We use content analysis, with a focus on particular terms, including "digitalization," "big data," "cloud computing," "blockchain," and "information technology" for 11 years, from 2011 to 2021. The frequency index from these keywords is developed to proxy the digital transformation for the Vietnamese listed firms. A final sample includes 118 Vietnamese listed firms with sufficient data for the analysis using the generalized method of moments (GMM) approach. The results indicate that corporate restructuring, including financial, portfolio, and operational restructuring, has a negative effect on firm performance in Vietnam. Digital transformation also negatively affects firm performance. However, corporate restructuring implemented in conjunction with digital transformation improves the performance of Vietnamese listed firms. These findings largely remain unchanged across various robustness analyses.

## 1. Introduction

In today’s rapidly changing business landscape, companies must continually adapt and innovate to remain competitive [1]. One of the most significant challenges facing modern firms is the need to embrace digital transformation [2]. The Fourth Industrial Revolution, also known as Industry 4.0, is characterized by integrating digital technologies into all aspects of business operations, from production to marketing and customer service [3, 4]. In this context, corporate restructuring can play a critical role in helping firms stay ahead of the curve.

Corporate restructuring refers to the process of making changes to a company’s organizational structure, business processes, or financial systems to improve efficiency and profitability [5]. Corporate restructuring emerges as a potent instrument for corporations navigating the complexities of modern business landscapes [6]. Financial, operational, and strategic restructuring collectively contribute to reshaping organizations, optimizing resources, and enhancing corporate performance [7]. Acknowledging the potential challenges and trade-offs inherent in restructuring processes is vital. When approached with strategic foresight and a comprehensive understanding of business dynamics, corporate restructuring becomes a pivotal driver of sustained success and competitiveness in the ever-evolving corporate landscape [8].

On the other hand, digital transformation refers to integrating digital technology into all aspects of a business, fundamentally changing how a company operates and delivers value to customers [9, 10]. The adoption of digital technology has been accelerating in recent years and significantly impacted firms’ performance [11]. Digital transformation can lead to improved operational efficiency. By automating processes and leveraging data analytics, firms can identify and eliminate bottlenecks, reduce manual errors, and streamline workflows. For example, companies can use machine learning algorithms to optimize supply chain management, reduce inventory costs and increase order fulfilment rates. These efficiencies can lead to cost savings, increased productivity, and faster time-to-market for new products and services [12, 13]. In addition, digital transformation can improve a firm’s agility and responsiveness to changing market conditions. By leveraging data and analytics, firms can gain real-time insights into market trends, customer preferences, and competitive landscapes. This approach allows companies to adapt quickly to changing market conditions and adjust their strategies accordingly. Furthermore, firms can use social media as a monitoring tool to track customer sentiment and respond to customer feedback promptly, leading to improved customer satisfaction and retention [14–16]. Moreover, digital transformation can enhance the customer experience. By leveraging digital technologies such as mobile apps, chatbots, and personalized marketing, firms can provide more engaging and personalized customer experiences. Doing so can increase customer loyalty, repeat business, and positive word-of-mouth referrals. For instance, companies can use data analytics to provide personalized product recommendations and targeted advertising campaigns, leading to higher conversion rates and revenue growth [14].

The COVID-19 pandemic has affected businesses worldwide, including in Vietnam, regardless of size. The pandemic has left business owners unsure about what actions to take, and their operations have been greatly impacted [15]. It is crucial for businesses to realize that sustainability is now a vital aspect of their operations in the face of an unexpected crisis [16]. Corporate restructuring is only considered necessary for large businesses already showing signs of revenue and profit decline. However, it is now essential for businesses to consider corporate restructuring even when external environmental factors require them to adapt and change their business models [15]. The Vietnamese Government views digital transformation as crucial to sustaining economic growth and well-being. Vietnam is recognized as having one of the most rapidly expanding digital economies in Southeast Asia. There is an anticipation that by 2030, the digital industry will account for 30 per cent of Vietnam’s GDP [17]. Hence, digital transformation is fundamental during the Fourth Industrial Revolution, when successful businesses utilize technology as a catalyst for growth and success [18].

Our literature review confirms the necessity for further research on the impact of corporate restructuring on firm performance in the Fourth Industrial Revolution, particularly for emerging markets such as Vietnam. Moreover, previous studies have neglected considering the combined effects of corporate restructuring and digital transformation on firm performance. These observations inspire us to conduct this study.

This study adds to the current literature in several ways. *First*, we investigate the independent impacts of corporate restructuring and digital transformation on firm performance, specifically in the context of the digital revolution and for emerging markets such as Vietnam. *Second*, the study examines how digital transformation can moderate the relationship between corporate restructuring and firm performance. This moderating effect of digital transformation has largely been ignored in the literature, particularly for emerging markets such as Vietnam. *Third*, the findings provide practical implications for managers regarding managing and utilizing digital transformation to gain long-term competitive advantages and improve operational efficiency in the digital age.

Following this introduction, the remainder of the study is structured as follows. The theoretical foundation is presented in Section 2. The methodology and data used in this study are described in Section 3. Section 4 presents and analyzes the empirical results. Finally, the study provides and discusses concluding remarks and managerial implications in Section 5 of the paper.

## 2. Literature review

### 2.1 Corporate restructuring and firm performance

Corporate restructuring includes mergers and acquisitions, divestitures, reorganizations, and changes to management or ownership structure [19]. Corporate restructuring can be broadly categorized into three types: financial restructuring, operational restructuring, and strategic restructuring [20]. Financial restructuring involves changing the firm’s capital structure, such as issuing new debt or equity to improve its financial position. Financial restructurings, such as debt restructurings or equity issuances, can also impact firm performance. Studies have found that financial restructuring can positively impact firm performance by improving liquidity and reducing financial distress [6, 8]. Financial restructuring can also have negative effects, such as diluting shareholder value and increased debt levels [7]. The operational restructuring aims to improve the efficiency and effectiveness of the firm’s operations, such as reducing costs and streamlining processes. Operational restructuring, such as cost-cutting and streamlining operations, can also impact firm performance. Studies have found that operational restructuring can positively impact firm performance by improving efficiency and reducing costs [21, 22]. However, operational restructuring can also have negative effects, such as decreased employee morale and reduced innovation [23, 24]. In contrast, strategic restructuring involves changing the firm’s business model, entering new markets, and divesting non-core assets. Divestitures and spin-offs involve selling or spinning off non-core assets or business units. On the one hand, various studies have generally found that divestitures and spin-offs positively impact firm performance by allowing it to focus on its core competencies and reduce costs [25]. These types of corporate restructuring can also improve financial performance by generating cash and reducing debt levels [26]. On the other hand, divestitures and spin-offs can also have negative effects, such as loss of synergies and reduced economies of scale [27].

### 2.2 Digital transformation and firm performance

While digital transformation has been hailed as a key driver of growth and innovation, particularly in developed markets [1, 28], its impact on firm performance in emerging markets, such as Vietnam, has been mixed [29]. In recent years, emerging markets have seen a rapid increase in digital transformation initiatives spurred by rising internet penetration, mobile phone usage, and the availability of low-cost digital tools [13, 29]. However, the negative impact of digital transformation on firm performance in emerging markets cannot be ignored [30]. One key challenge is the digital divide that exists in emerging markets. While internet penetration is increasing, there are still significant gaps in access to digital tools and infrastructure, particularly in rural areas. This can lead to a digital divide between urban and rural areas and between different income groups, limiting the potential benefits of digital transformation for firms [31]. Another challenge is the skills gap that exists in many emerging markets. While there is a growing pool of digital talent in some countries, many firms struggle to find and retain skilled workers with the necessary expertise in data analytics, cybersecurity, and software development. This weakness can limit firms’ ability to fully leverage digital transformation’s potential [30]. Digital transformation can be costly and time-consuming for firms, particularly those in emerging markets with limited financial resources and no experience implementing digital technologies. Digital transformation can result in high upfront costs and disruption to existing business processes, negatively impacting firm performance in the short term [32]. Moreover, the rapid pace of technological change and innovation in the digital space can also pose challenges for firms in emerging markets. Keeping up with the latest developments and trends can be difficult, particularly for smaller firms with limited resources, and failure to do so can result in a loss of market share and competitive advantage [33]. Digital transformation can also result in significant changes to customer behaviour and expectations, which can pose challenges for firms in emerging markets. For example, increased use of e-commerce and mobile payments can make it difficult for firms to compete on price and differentiate their products and services while also increasing the need for strong customer service and digital marketing capabilities [34, 35].

### 2.3 The moderating role of digital transformation in the relationship between corporate restructuring and firm performance

Corporate restructuring entails a strategic overhaul of organizational components such as financial structures, operational processes, and strategic frameworks [6]. Corporate restructuring has been employed to optimize resource allocation, enhance operational efficiency, and align businesses with evolving market demands [25]. The success of restructuring initiatives traditionally hinged on effective implementation and adaptability to changing circumstances [27]. Simultaneously, the advent of digital transformation has ushered in a paradigm shift in the way businesses operate [34]. Characterized by the infusion of technology into various aspects of organizational processes, digital transformation aims to improve agility, innovation, and overall competitiveness [32]. This includes the integration of data analytics, automation, artificial intelligence, and other cutting-edge technologies to streamline operations and create new value propositions. Digital transformation operates as a moderating force in the relationship between corporate restructuring and corporate performance. In the context of restructuring initiatives, digital transformation moderates the effectiveness of these endeavors by influencing the speed, depth, and adaptability of change. Leveraging digital tools can enhance the efficiency of restructuring processes, allowing organizations to respond more adeptly to market shifts and dynamic customer expectations [14].

In recent years, emerging markets have witnessed a rapid surge in digital transformation initiatives propelled by increased internet accessibility, mobile phone usage, and the availability of low-cost digital tools [13]. However, the limitations of digital transformation on firm performance in emerging markets cannot be overlooked [30]. A significant challenge is the existing digital divide in these markets. Despite the growing internet access, substantial gaps persist in accessing digital tools and infrastructure, especially in rural areas. This may lead to a digital divide between urban and rural areas, as well as among different income groups, limiting the benefits of digital transformation for companies [31]. Another challenge is the scarcity of skills and knowledge in implementing digital transformation in many emerging markets. While the number of digital workforces is increasing in some countries, many companies still struggle to recruit and retain employees with necessary skills in data analysis, cybersecurity, and software development [30]. Moreover, the rapid pace of technological change and innovation in the digital space can pose challenges for companies in emerging markets like Vietnam. Keeping up with the latest developments and trends can be challenging, especially for small companies with limited resources, and failing to do so may result in market share loss and competitive disadvantage [33].

Digital transformation can enhance the production and business management processes. By applying information technology, automation, and artificial intelligence, companies can optimize production processes, minimize human errors, and increase productivity [15]. Vietnam is participating in the global market, and competition is intensifying. Digital transformation helps businesses in Vietnam enhance competitiveness by improving product quality, reducing production costs, and quickly adapting to changes in international market demands [16]. Digital transformation enables businesses to establish strong connections in the supply chain, facilitating more efficient management of inventory, transportation, and customer demand forecasting [15]. Therefore, examining the moderating role of digital transformation in the relationship between corporate restructuring and firm performance in an emerging market like Vietnam is crucial.

## 3. Model and data

### 3.1 Research model

Based on the empirical model from [35, 36], this paper investigates the impact of corporate restructuring on firm performance in the presence of digital transformation for 118 Vietnamese listed firms from 2011 to 2021 using the following model:

$${\text{ROA}}_{it}=\beta_{0}+\beta_{1}{\text{DT}}_{it}+\beta_{2}{\text{FR}}_{it}+\beta_{3}{\text{PR}}_{it}+\beta_{4}{\text{OR}}_{it}+\beta_{5}{\text{SIZE}}_{it}+\varepsilon_{it}$$ (1)

in which *i* and *t* represent a firm and time, respectively. ROA represents the return on assets. DT denotes digital transformation. FR represents financial restructuring. PR signifies portfolio restructuring. OR denotes operational restructuring, and SIZE is the natural logarithm of the total assets.

While corporate restructuring (including financial, portfolio, and operational) may affect firm performance, we examine the effects of corporate restructuring on firm performance. In addition, we would also like to test whether digital transformation moderates the effects of corporate restructuring on firm performance. Thus, we add an interaction term constructed as the product of each kind of corporate restructuring (including financial restructuring, portfolio restructuring, and operational restructuring) and digital transformation (DT*FR, DT*PR and DT*OR) to Eq (1) as an additional explanatory variable. The regression is as below:

$${\text{ROA}}_{it}=\beta_{0}+\beta_{1}{\text{DT}}_{it}+\beta_{2}{\text{FR}}_{it}+\beta_{3}{\text{PR}}_{it}+\beta_{4}{\text{OR}}_{it}+\beta_{5}\text{DT}*{\text{FR}}_{it}+\beta_{6}\text{DT}*{\text{PR}}_{it}+\beta_{7}\text{DT}*{\text{OR}}_{it}+\beta_{8}{\text{SIZE}}_{it}+\varepsilon_{it}$$ (2)

The focus of this study is on the digital transformation of Vietnamese-listed firms. The language used in the annual reports of these firms can reveal their plans and strategic direction for digital transformation [36]. In addition, [37] suggests that the frequency of a term in an annual report indicates its significance level. When it comes to measuring large sample texts, [38] recommends using the word frequency approach as the most suitable quantitative method. Therefore, analyzing the text in the annual reports of listed companies can effectively show their strategic orientation [39]. "Digital transformation" is an essential development strategy, and the related words and phrases are likely to be present in the annual reports of these companies. Thus, it is feasible and reasonable to use word frequency statistics from the featured lexicon involving "digital transformation" within annual reports to depict the level of digital transformation of listed companies [40].

To begin the process, the first step is to choose the most fitting keywords that accurately represent the digital transformation behaviour of the company based on the initial seed word. Next, similar words are developed by performing Vietnamese word segmentation using Word Embedding on the company’s annual reports. The words with high similarity to the seed word are then selected, with a manual review of those words that have more than 50 per cent similarity to the original word. Finally, the validity of the keyword set is checked through a correlation analysis of the frequency of the chosen keywords. The results show that the keywords are highly correlated. Furthermore, many of these words are statistically significant, with "digital transformation" at a significance level of 5 per cent or higher. This finding indicates that the selected set of words is appropriate.

The core technologies for digital transformation are artificial intelligence (AI), blockchain, cloud computing, and big data [36]. This paper incorporates Vietnamese terms including "digitalization," "big data," "cloud computing," "blockchain," and "information technology" for statistical measurement. Data for this study is manually collected from the annual reports of listed companies over 11 years, from 2011 to 2021. The frequency of the above-featured words is counted to create a total word frequency. The natural logarithm of the total frequency is used as an index to evaluate the digital transformation performance of listed firms. Firms that do not have complete data for at least five years are not considered. Following the data cleaning process, 118 of the 747 firms listed in Vietnam are available for further analysis.

In addition, corporate restructuring is measured through the variables of financial restructuring, portfolio restructuring and operational restructuring [21, 22], which can be summarised as follows:

- FR is financial restructuring, measured by the change in the total debt-to-equity ratio.
- PR denotes portfolio restructuring, measured by the change in the ratio of fixed assets to total assets.
- OR signifies operational restructuring, measured by the change in operating expenses over total income.

In addition, principal component analysis (PCA) method is also used to build the corporate restructuring (CR) variable, as follows:

$$\text{CR}=\sum_{i=1}^{n}\omega_{i}X_{i}$$

*Where*: ω_i_ is the component loadings or weights, and X_i_ is corporate restructuring components: financial restructuring (FR), portfolio restructuring (PR), and operational restructuring (OR).

The study employs the natural logarithm of the total assets (SIZE). The selection of the control variable is based on its significant impact on a company’s performance, as established by [41]. In addition, this control variable has been used in previous studies on firm performance, including those conducted by [42, 43].

### 3.2 Data

This study uses information obtained from annual reports of 118 listed corporates in Vietnam from 2011 to 2021. The necessary information is readily accessible, but certain data are missing. Consequently, the study relies on an unbalanced sample size of 1,251 observations over the stated period.

## 4. Empirical results and discussions

### 4.1. The descriptive statistics

Tables 1 and 2 present the descriptive statistics and correlation matrix between all variables. The range of the dependent variable, ROA, is between 0.001 and 0.486, with an average value of 0.076. The average value of digital transformation is 3.089, with a standard deviation of 1.171. The mean values of financial restructuring, portfolio restructuring, and operational restructuring are -0.032, -0.001, and 0.231, respectively. The large standard deviations of the variables suggest they are widely dispersed around their means.

**Table 1:** The descriptive statistics of the entire sample.

Variables	Observations	Mean	Min.	Max.	Std. Dev.
ROA	1,223	0.076	0.001	0.486	0.066
DT	1,251	3.089	0.000	6.000	1.171
FR	1,226	-0.032	-13.282	2.816	0.716
PR	1,224	-0.001	-0.696	0.823	0.055
OR	1,202	0.231	-70.526	125.012	6.014
SIZE	1,240	7.003	2.833	20.827	1.937

*Notes*: **ROA** represents the return on assets; **DT** denotes digital transformation; **FR** represents financial restructuring; **PR** signifies portfolio restructuring; **OR** denotes operational restructuring; and **SIZE** is the natural logarithm of the total assets.

**Table 2:** The pairwise correlation matrix.

Variable	ROA	DT	FR	PR	OR	SIZE
ROA	1.0000					
DT	-0.039	1.0000				
FR	-0.061**	0.020	1.0000			
PR	-0.074***	-0.001	-0.057**	1.0000		
OR	-0.104***	-0.007	0.016	0.064**	1.0000	
SIZE	-0.229***	0.006	-0.016	0.013	0.024	1.0000

Notes:

** and *** are significant at 5 per cent and 1 per cent levels, respectively

**ROA** represents the return on assets; **DT** denotes digital transformation; **FR** represents financial restructuring; **PR** signifies portfolio restructuring; **OR** denotes operational restructuring; and **SIZE** is the natural logarithm of the total assets.

The Fisher-type test, proposed by [44], is based on the idea of estimating the optimal number of lags for an autoregressive model using the Akaike Information Criterion (AIC) or the Bayesian Information Criterion (BIC) and then applying the Fisher-type test to the estimated residuals of the model. The test statistic is calculated as the sum of squares of the estimated residuals for the optimal lag length, normalized by an estimate of the asymptotic variance. The null hypothesis of non-stationarity is rejected if the test statistic exceeds a critical value from the Fisher distribution. As presented in Table 3, the test results indicate that the variables used in our analysis are stationary.

**Table 3:** The panel unit-root test results.

Variables	Inverse chi-squared	Inverse normal	Inverse logit	Modified inv. chi-squared
ROA	507.287***	-2.900***	-6.136***	12.487***
DT	515.243***	-7.012***	-9.191***	12.853****
FR	674.590***	-8.129***	-12.551***	20.366***
PR	1035.433***	-12.154***	-22.260***	37.046***
OR	941.773***	-11.369***	-19.729***	32.716***
DT*FR	808.420***	-9.672***	-16.153***	26.552***
DT*PR	1087.146***	-13.042***	-23.909***	39.436***
DT*OR	1019.375***	-12.285***	-21.893***	36.304***
SIZE	349.393***	3.414	2.036	5.219***

Notes:

*** is significant at 1 per cent.

**ROA** represents the return on assets; **DT** denotes digital transformation; **FR** represents financial restructuring; **PR** signifies portfolio restructuring; **OR** denotes operational restructuring; DT*FR, DT*PR and DT*OR are interaction variables; and **SIZE** is the natural logarithm of the total assets.

Previous studies [45, 46] argue that panel data often exhibits heteroskedasticity and autocorrelation. Heteroskedasticity occurs when the variability of the residuals (i.e., the differences between the actual and predicted values of the dependent variable) changes as the values of the independent variables change. This violation of the assumption of homoskedasticity (i.e., the constant variance of errors) can lead to biased and inconsistent estimates of regression coefficients and standard errors, affecting the validity of statistical inferences and predictions based on the model [47]. Meanwhile, autocorrelation can lead to biased and inefficient estimates of regression coefficients, as well as inflated standard errors and invalid hypothesis testing [48]. In this study, the modified Wald and Wooldridge tests are employed to investigate the presence of heteroskedasticity and autocorrelation in our two models. Based on the results presented in Table 4, it is concluded that both heteroskedasticity and autocorrelation are present in the analysis.

**Table 4:** The modified Wald & Wooldridge test results.

	Wooldridge test			Modified Wald test		
	F-test	*p*-value	Presence of autocorrelation	χ^2^	*p*-value	Presence of heteroskedasticity
Eq 1	6.186	0.014	√	90684.29	0.000	√
Eq 2	6.552	0.011	√	83276.60	0.000	√

### 4.2. The moderating role of digital transformation on the relationship between corporate restructuring and firm performance

We utilize the generalized method of moments (GMM) to investigate how digital transformation moderates the effects of corporate restructuring on firm performance. This statistical technique can deal with various problems in panel estimation, such as autocorrelation, heteroscedasticity, and endogeneity, which could affect the model’s results [49, 50]. The GMM method estimates the parameters by minimizing the difference between the observed data and the model’s predicted values based on certain moment conditions that reflect the characteristics of the data-generating process [51].

Empirical results from Eq (1) are presented in Table 5. Firm performance is adversely affected by corporate restructuring, including financial restructuring, portfolio restructuring, and operational restructuring. This finding is consistent with previous studies [23, 52, 53]. These findings provide additional evidence of the drawbacks of corporate restructuring in Vietnam. Various factors can be used to explain the negative effects of corporate restructuring on firm performance. The first reason is a lack of managerial competence in dealing with non-traditional company activities [23]. Any revenue gains from corporate restructuring could be quickly offset by the expenses associated with non-traditional activities [54]. Firm managers may be prone to the trap of novelty, ignoring core company activities [52]. Besides, operational restructuring can result in the loss of key personnel or the disruption of critical relationships with suppliers or customers, adversely affecting company performance over time [53]. As a result, firm managers should concentrate on strategies to increase core revenue to avoid potential losses from restructuring activities.

**Table 5:** The moderating role of digital transformation on the relationship between corporate restructuring and firm performance.

Variables	Eq 1	Eq 2
ROA_t-1_	0.455***	0.448***
DT	0.001	-0.017***
FR	-0.007***	-0.018***
PR	-0.031**	0.144***
OR	-0.001***	-0.002***
DT*FR		0.003***
DT*PR		0.056***
DT*OR		0.001***
SIZE	0.059***	-0.002***
_cons	0.059***	0.113***
AR (2) test	0.176	0.131
Sargan test	0.000	0.000
Hansen test	0.258	0.313

Notes:

*, ** and *** are significant at 10 per cent, 5 per cent and 1 per cent levels, respectively

**ROA** represents the return on assets; **DT** denotes digital transformation; **FR** represents financial restructuring; **PR** signifies portfolio restructuring; **OR** denotes operational restructuring; DT*FR, DT*PR and DT*OR are interaction variables; and **SIZE** is the natural logarithm of the total assets.

In addition, our findings confirm the negative effects of digital transformation on firm performance in Vietnam. These findings align with previous studies [11, 32]. Small businesses in emerging markets often face the common obstacle of digital transformation [33]. [11] highlight that established companies resist digital transformation due to counterproductive habits or dependence on traditional approaches. Vietnamese firms continue encountering numerous obstacles concerning digital transformation, including corporate culture and employee expertise, infrastructure and technology, and ecosystems. Consequently, these effects of digital transformation have resulted in a detrimental effect on firm performance in Vietnam.

Interestingly, our results confirm that the joint effects of corporate restructuring and digital transformation boost firm performance. These findings indicate that corporate restructuring should be implemented in conjunction with digital transformation. One of the key benefits of corporate restructuring is that the process can help firms become more efficient and productive. For example, by consolidating operations or divesting non-core assets, firms can focus on their core competencies and improve their overall performance [21, 22]. Corporate restructuring, when conducted together with digital transformation, can also help firms reduce costs, streamline processes, and improve decision-making, all of which can positively impact firm performance [26, 55].

### 4.3. The robustness analysis

To further examine the link between corporate restructuring, digital transformation, and firm performance, we utilize the principal component analysis (PCA) method to develop a corporate restructuring index. PCA can reduce the potential for multicollinearity, which occurs when there is a high correlation among variables in a data set. Multicollinearity can cause problems when constructing indices because it can lead to unstable coefficients and make it difficult to interpret the relationships among variables [56]. PCA can help address this issue by creating a new set of uncorrelated variables, which can improve the accuracy and reliability of the index [57]. We construct the corporate restructuring index (CR) as follows:

$$\text{CR}=\sum_{i=1}^{n}\omega_{i}X_{i}$$

Where: ω_i_ represents the component loadings or weights, and X_i_ denotes the original proxy, including financial restructuring, portfolio restructuring and operational restructuring.

In addition, we divide our sample of Vietnamese listed firms into two categories: technology firms and non-technology firms. [58, 59] consider that technology firms that utilize technological superiority as a critical component of their approach are expected to gain the most from digital transformation. Empirical findings from our robustness analysis are presented in Table 6.

**Table 6:** The robustness analysis—The moderating role of digital transformation on the corporate restructuring—Firm performance nexus.

Variables	All firms	Technology firms	Non-technology firms
ROA_t-1_	0.517***	0.927***	0.503***
DT	-0.022***	0.002**	-0.001
CR	-0.008***	-0.011***	-0.048**
DT*CR	0.001**	0.004**	0.011*
SIZE	-0.002**	-0.001	-0.004***
_cons	0.120***	0.003	0.069***
AR (2) test	0.119	0.572	0.128
Sargan test	0.000	0.972	0.000
Hansen test	0.155	0.896	0.377

Notes:

*, ** and *** are significant at 10 per cent, 5 per cent and 1 per cent levels, respectively.

**ROA** represents the return on assets; **DT** denotes digital transformation; **CR** represents corporate restructuring; DT*CR is interaction variables; and **SIZE** is the natural logarithm of the total assets.

The results shown in Table 6 reinforce the moderating role of digital transformation in the connection between corporate restructuring and firm performance for both technology and non-tech firms in Vietnam. Besides, our findings also suggest that digital transformation is a crucial factor driving the performance of technology firms, which aligns with earlier research [60, 61]. Furthermore, there is a consensus among experts that digital transformation can enhance production flexibility, boost output capacity, and improve product quality for technology firms [62]. However, there is no significant correlation between digital transformation and the performance of non-technology firms.

## 5. Concluding remarks and managerial implications

The Fourth Industrial Revolution, the industry 4.0 era, has significantly changed the global economy. Companies that fail to adapt risk being left behind. Corporate restructuring has become necessary for companies to remain competitive in this new era. Through corporate restructuring, companies can align with new market demands, optimize their operations, foster innovation and agility, and attract and retain top talent. Firms that embrace restructuring are better equipped to succeed in the industry 4.0 era and create value for their shareholders, employees, and customers. However, corporate restructuring is not obviously linked with success given its significant costs involved for firms, particularly in emerging markets.

As such, this study examines the moderating effects of digital transformation on the corporate restructuring—firm performance nexus. Our study employs the generalized method of moments (GMM) estimation technique for panel data of 118 listed firms in Vietnam from 2011 to 2021. The results are summarized as follows. Corporate restructuring, including financial restructuring, portfolio restructuring, and operational restructuring, has a significant and negative impact on the performance of firms in Vietnam. Digital transformation also appears to affect the performance of listed firms in Vietnam negatively. However, corporate restructuring in conjunction with digital transformation can be a driving force to improve firm performance in Vietnam. These findings are robust with different settings, including developing the corporate restructuring index and sub-samples of technology and non-tech firms.

Managerial implications have emerged based on the findings from our study. *First*, digital transformation has the potential to drive growth and innovation. However, it can also have a negative impact on firm performance in emerging markets such as Vietnam. The digital divide, skills gap, upfront costs, technological change, and changes in customer behaviour pose significant challenges that firms must overcome to leverage the potential benefits of digital transformation fully. As such, it is crucial for firms in emerging markets to carefully consider the costs and benefits of digital transformation and develop a comprehensive strategy that considers the unique challenges and opportunities of their restructuring context. *Second*, the process of digitizing listed firms in Vietnam cannot be accomplished quickly. Each firm should conduct a comprehensive analysis and restructuring in accordance with its characteristics and size of operation. *Third*, to achieve successful corporate restructuring, firm managers need to confront the intricate and ever-changing connections between emerging digital technologies and the trade-offs associated with restructuring concerning their financial, portfolio and operational activities, which are becoming increasingly uncertain and complex. *Final*ly, the Vietnamese Government should implement customized incentive policies to encourage corporate restructuring during the digitalization era. Unique policies should be implemented to address issues such as financial challenges, lack of digital talent, and insufficient planning for digital strategies. By doing so, these firms can more effectively achieve their digital transformation, stay current with the digital age, and enhance their performance.

## Supporting information

S1 Annex (PDF)

## References

[1] Schonherr S, Eller R, Kallmuenzer A, Peters M. Organizational learning and sustainable tourism: the enabling role of digital transformation. J. Knowl. Manag. 2023; Vol. ahead-of-print No. ahead-of-print. doi: 10.1108/JKM-06-2022-0434

[2] Goldfarb A, Tucker C. Digital economics. *J*. *Econ*. *Lit*. 2019; 57(1):3–43.

[3] Gong C, Ribiere V. Understanding the role of organizational agility in the context of digital transformation: an integrative literature review. VINE J. Inf. Knowl. Manag. Syst. 2023; Vol. ahead-of-print No. ahead-of-print. doi: 10.1108/VJIKMS-09-2022-0312

[4] Zhao F, Wallis J, Singh M. E-government development and the digital economy: a reciprocal relationship. Internet Res. 2015; 25(5):734–766.

[5] Agrawal A, Goldfarb A. Restructuring research: Communication costs and the democratization of university innovation. Am. Econ. Rev. 2008; 98(4):1578–1590. doi: 10.1257/aer.98.4.1578

[6] Denis DJ, Kruse TA. Managerial discipline and corporate restructuring following performance declines. J. Financ. Econ. 2000; 55(3):391–424.

[7] Cruz-García P, Forte A, Peiró-Palomino J. On the drivers of profitability in the banking industry in restructuring times: a Bayesian perspective. Appl. Econ. Ana. 2020; 28(83):111–131.

[8] Dzingirai M. Demystifying corporate restructuring strategy through digital transformation: lessons learned from the banking sector of Zimbabwe. In *Emerging challenges*, *solutions*, *and best practices for digital enterprise transformation* (pp. 164–181). IGI Global; 2021.

[9] Gil-Gomez H, Guerola-Navarro V, Oltra-Badenes R, Lozano-Quilis JA. Customer relationship management: Digital transformation and sustainable business model innovation. Econ. Res.-Ekon. Istraz. 2020; 33(1):2733–2750. doi: 10.1080/1331677X.2019.1676283

[10] Mucha T, Ma S, Abhari K. Riding a bicycle while building its wheels: the process of machine learning-based capability development and IT-business alignment practices. Internet Res. 2023; 33(7):168–205.

[11] Magnusson J, Elliot V, Hagberg J. Digital transformation: why companies resist what they need for sustained performance. J. Bus. Strategy. 2022; 43(5):316–322.

[12] Bhatti A, Malik H, Kamal AZ, Aamir A, Ullah Z. Much-needed digital business transformation through big data, internet of things and blockchain capabilities: implications for strategic performance in telecommunication sector. Bus. Process Manag. J. 2021; 27(6):1854–1873.

[13] Liu QL, Zhang Y, Lei YY, Chen GJ. Research on process, logic and implementation mechanism of digital enabling enterprise innovation. Stud. Sci. Sci. 2022; 40(1):150–159.

[14] Shan Y, Xu H, Zhou LX, Zhou Q. Digital and intelligent empowerment: how to form organizational resilience in crisis?: an exploratory case study based on forest cabin’s turning crisis into opportunity. Manag. World, 2021; 37(3):84–104.

[15] Le LHV, Huynh TLD, Weber BS, Nguyen BKQ. Different firm responses to the COVID-19 pandemic shocks: machine-learning evidence on the Vietnamese labour market. Int. J. Emerg. Mark. 2023; 18(9):2501–2522.

[16] Nguyen KD, Pham HX, Truong QT, Nguyen HM. The COVID-19 Pandemic Impact and Responses in Emerging Economies: Evidence from Vietnamese Firms. Econom. 2023; 11(1):10. doi: http%3A//dx.doi.org/10.3390/economies11010010

[17] Source of Asia. Vietnam’s digital transformation Outlook 2022. https://www.sourceofasia.com/vietnams-digital-transformation-outlook-2022/ (accessed 12 March 2023)

[18] Nguyen TV. Strategy, Culture, Human Resource, IT Capability, Digital Transformation and Firm Performance—Evidence from Vietnamese Enterprises. In Sriboonchitta S., Kreinovich V., Yamaka W. *(eds)* *Behavioral Predictive Modeling in Economics*. *Studies in Computational Intelligence*, vol 897. Springer, Cham; 2021.

[19] Best MH. The new competition: institutions of industrial restructuring. Harvard University Press; 1990

[20] Gaughan PA. Mergers, acquisitions, and corporate restructurings. John Wiley & Sons; 2010.

[21] Bartlett CA, Ghoshal S. Managing across borders: The transnational solution. Harvard Business School Press; 1990

[22] Sebastian IM, Ross JW, Beath C, Mocker M, Moloney KG, Fonstad NO. How big old companies navigate digital transformation. MIS Q. Exec. 2017; 16(3):197–213.

[23] Githaiga PN. Human capital, income diversification and bank performance—an empirical study of East African banks. Asian J. Account. Res. 2021; 6(1):95–108.

[24] Celine B, Cédric D. Financialization, globalization and the making of profits by leading retailers. Socio-Econ. Rev. 2012; 10(2):241–266.

[25] Cheng RCN, Men X, Hsieh JJPA, Cheng ZJ, Cui X, Wang T, et al. The effects of IT chargeback on strategic alignment and performance: the contingent roles of business executives’ IT competence and CIOs’ business competence. Internet Res. 2023; 33(1):57–83.

[26] Khin S, Ho TC. Digital technology, digital capability and organizational performance: A mediating role of digital innovation. Int. J. Innov. Sci. 2020; 11(2):177–195.

[27] Sherman HD, Rupert TJ. Do bank mergers have hidden or foregone value? Realized and unrealized operating synergies in one bank merger. Eur. J. Oper. Res., 2006; 168(1):253–268.

[28] Lin Q. The theoretical framework of enterprise digital innovation: insights from a qualitative meta-analysis. Eur. J. Innov. Manag. 2023; Vol. ahead-of-print No. ahead-of-print. doi: 10.1108/EJIM-09-2022-0496

[29] Zhang T, Shi ZZ, Shi YR, Chen NJ. Enterprise digital transformation and production efficiency: mechanism analysis and empirical research. Econ. Res.-Ekon. Istraz. 2022; 35(1):2781–2792.

[30] Nell PC, Foss NJ, Klein PG, Schmitt J. Avoiding digitalization traps: tools for top managers. Bus. Horiz. 2021; 64(2):163–169

[31] Tekic Z, Koroteev D. From disruptively digital to proudly analogue: a holistic typology of digital transformation strategies. Bus. Horiz. 2019; 62(6):683–693.

[32] Brunetti F, Matt DT, Bonfanti A, De Longhi A, Pedrini G, Orzes G. Digital transformation challenges: strategies emerging from a multi-stakeholder approach. Total Qual. Manag. 2020; 32(4):697–724.

[33] Heredia J, Castillo-Vergara M, Geldes C, Gamarra FMC, Flores A, Heredia W. How do digital capabilities affect firm performance? The mediating role of technological capabilities in the "new normal”. J. Innov. Knowl. 2022; 7(2):100171. doi: 10.1016/j.jik.2022.100171

[34] Garcez A, Franco M, Silva R. The soft skills bases in digital academic entrepreneurship in relation to digital transformation. Innov. Manag. Rev. 2023; 20(4):393–408.

[35] Zhang Y, Li H, Yao Z. Intellectual capital, digital transformation and firm performance: evidence based on listed companies in the Chinese construction industry. Eng. Constr. Archit. Manag. 2023; Vol. ahead-of-print No. ahead-of-print. doi: 10.1108/ECAM-06-2023-0623

[36] Zhao X, Sun X, Zhao L, Xing Y. Can the digital transformation of manufacturing enterprises promote enterprise innovation? Bus. Process Manag. J. 2022; 28(4):960–982.

[37] Unerman J. Methodological issues—Reflections on quantification in corporate social reporting content analysis. Account. Audit. Account. J. 2000; 13(5):667–681.

[38] Roberts CW. A theoretical map for selecting among text analysis methods In *Text Analysis for the Social Sciences; Roberts*, *C*.*W*., *Ed*.; Lawrence Erlbaum Associates: Mahway, NJ, USA; 1997.

[39] Wu JZ, Xiao SF. Innovation attention shift, R&D spending leap and firm performance: evidence from China. Nankai Bus. Rev. 2016; 19(2):182–192.

[40] Chen QJ, Wang YM, Wan MF. Research on peer effect of enterprise digital transformation and influencing factors. Chin. J. Manag. 2021; 18(5):653–663.

[41] Fama E, French KR. The cross-section of expected stock returns. J. Finance. 1992; 47(2):427–465.

[42] Asiaei K, Rezaee Z, Bontis N, Barani O, Sapiei NS. Knowledge assets, capabilities and performance measurement systems: a resource orchestration theory approach. J. Knowl. Manag. 2021; 25(8):1947–1976.

[43] Sukumar A, Jafari-Sadeghi V, Garcia-Perez A, Dutta DK. The potential link between corporate innovations and corporate competitiveness: evidence from IT firms in the UK. J. Knowl. Manag. 2020; 24(5):965–983.

[44] Choi I. Unit root tests for panel data. J. Int. Money Finance. 2001; 20:249–272.

[45] Hill AD, Johnson SG, Greco LM, O’Boyle EH, Walter SL. Endogeneity: A Review and Agenda for the Methodology-Practice Divide Affecting Micro and Macro Research. J. Manage. 2021; 47(1):105–143.

[46] Tran NP, Vo DH. Do banks accumulate a higher level of intellectual capital? Evidence from an emerging market. J. Intellect. Cap. 2022; 23(2):439–457.

[47] Baum CF, Schaffer ME, Stillman S. Instrumental variables and GMM: Estimation and testing. *Stata J*. 2003; 3(1):1–31.

[48] Andrews DWK. Heteroskedasticity and autocorrelation consistent covariance matrix estimation. Econom. 1991; 59:817–858.

[49] Arellano M, Bover O. Another look at the instrumental variable estimation of error-components models. J. Econom. 1995; 68(1):29–51.

[50] Xaisongkham S, Liu X. Institutional quality, employment, FDI and environmental degradation in developing countries: evidence from the balanced panel GMM estimator. Int. J. Emerg. Mark. 2022 Vol. ahead-of-print No. ahead-of-print. doi: 10.1108/IJOEM-10-2021-1583

[51] Blundell R, Bond S. Initial conditions and moment restrictions in dynamic panel data models. J. Econom. 1998; 87(1):115–143.

[52] Stiroh KJ, Rumble A. The dark side of diversification: the case of US financial holding companies. J. Bank. Finance. 2006; 30(8):2131–2161.

[53] Haspeslagh PC, Jemison DB. Managing acquisitions: Creating value through corporate renewal. Free Press; 1991. https://books.google.com.vn/books?id=t-5sQgAACAAJ

[54] McArthur J. Mergers and Acquisitions: The Human Factor. Handbook Bus. Strategy. 2000; 1(1):217–223.

[55] Zyglidopoulos SC. The Impact of Downsizing on Corporate Reputation. Br. J. Manag. 2005; 16(3):253–259.

[56] Ahamed MM, Mallick S. Is financial inclusion good for bank stability? International evidence. J. Econ. Behav. Organ. 2019; 157:403–427.

[57] Agarwal T, Goel PA, Gartaula H, Rai M, Bijarniya D, Rahut DB, et al. Gendered impacts of climate-smart agriculture on household food security and labour migration: insights from Bihar, India. Int. J. Clim. Change Strateg. Manag. 2022; 14(1):1–19.

[58] Del Giudice M, Scuotto V, Papa A, Tarba SY, Bresciani S, Warkentin M. A self-tuning model for smart manufacturing SMEs: effects on digital innovation. *J*. *Prod*. *Innov*. *Manag*. 2021; 38(1):68–89.

[59] Thomson L. Leveraging the value from digitalization: a business model exploration of new technology-based firms in vertical farming. J. Manuf. Technol. Manag. 2022; 33(9):88–107.

[60] Shen Q, Hua Y, Huang Y, Ebstein R, Yu X, Wu Z. Knowledge management and modern digital transformation of the property management industry in China. J. Knowl. Manag. 2022; 26(8):2133–2144.

[61] Tang H, Xie Y, Liu Y, Boadu F. Distributed innovation, knowledge re-orchestration, and digital product innovation performance: the moderated mediation roles of intellectual property protection and knowledge exchange activities. J. Knowl. Manag. 2023; Vol. ahead-of-print No. ahead-of-print. doi: 10.1108/JKM-07-2022-0592

[62] Buchi G, Cugno M, Castagnoli R. Smart factory performance and Industry 4.0. Technol. Forecast. Soc. Change. 2020; 150:119790, October 2019, doi: 10.1016/j.techfore.2019.119790

