# Supplementary material for: Corporate restructuring and firm performance in Vietnam: The moderating role of digital transformation
Source: PLoS One. 2024 May 20;19(5):e0303491. doi: 10.1371/journal.pone.0303491 (PMC11104660; doi:10.1371/journal.pone.0303491)
Supplement: S1 Annex — (PDF) [file pone.0303491.s001.pdf]

ID	YEAR	FR	PR	OR	SIZE	ROA	DT*FR	DT*PR	DT*OR	DT	CR	DT*CR
BVH	2011	-	-	-	10.68	0.03	-	-	-	3.31	-	-
BVH	2012	0.02	(0.00)	(0.81)	10.74	0.04	0.08	(0.02)	(2.97)	3.68	(0.24)	(0.87)
BVH	2013	-	-	-	10.92	0.03	-	-	-	3.45	-	-
BVH	2014	(0.53)	0.01	(0.31)	10.77	0.03	(1.85)	0.02	(1.07)	3.47	(0.28)	(0.98)
BVH	2015	-	-	-	10.98	0.03	-	-	-	3.13	-	-
BVH	2016	0.90	(0.01)	0.93	11.20	0.02	2.56	(0.02)	2.66	2.86	0.60	1.72
BVH	2017	-	-	-	11.42	0.02	-	-	-	2.87	-	-
BVH	2018	1.00	(0.00)	1.77	11.64	0.01	2.96	(0.01)	5.25	2.97	0.89	2.65
BVH	2019	-	-	-	11.76	0.01	-	-	-	3.62	-	-
BVH	2020	(0.02)	(0.00)	(0.25)	11.76	0.01	(0.08)	(0.00)	(0.90)	3.58	(0.08)	(0.30)
BVH	2021	1.28	(0.00)	(1.56)	12.04	0.01	4.45	(0.01)	(5.44)	3.49	(0.01)	(0.05)
PVI	2011	-	-	-	9.01	0.06	-	-	-	3.23	-	-
PVI	2012	0.27	0.03	(0.80)	9.28	0.05	0.89	0.10	(2.64)	3.28	(0.14)	(0.45)
PVI	2013	-	-	-	9.43	0.04	-	-	-	3.01	-	-
PVI	2014	0.96	(0.06)	(0.83)	9.82	0.02	2.79	(0.17)	(2.40)	2.90	0.08	0.22
PVI	2015	-	-	-	9.65	0.05	-	-	-	2.80	-	-
PVI	2016	0.14	0.00	(0.25)	9.72	0.04	0.80	0.00	(1.47)	5.77	(0.03)	(0.15)
PVI	2017	-	-	-	9.88	0.03	-	-	-	4.27	-	-
PVI	2018	(0.08)	(0.00)	0.16	9.89	0.04	(0.21)	(0.00)	0.41	2.61	0.02	0.05
PVI	2019	-	-	-	10.00	0.04	-	-	-	2.15	-	-
PVI	2020	0.01	0.00	(0.09)	10.01	0.05	0.02	0.00	(0.15)	1.62	(0.02)	(0.04)
PVI	2021	0.01	(0.00)	0.08	10.10	0.05	0.01	(0.01)	0.16	2.06	0.02	0.05
BIC	2011	-	-	-	7.53	0.05	-	-	-	2.67	-	-
BIC	2012	(0.43)	0.00	(1.00)	7.36	0.07	(1.00)	0.01	(2.32)	2.32	(0.46)	(1.06)
BIC	2013	-	-	-	7.47	0.07	-	-	-	4.00	-	-
BIC	2014	1.17	(0.00)	(0.24)	8.03	0.04	3.69	(0.01)	(0.76)	3.16	0.35	1.09
BIC	2015	-	-	-	8.38	0.04	-	-	-	1.82	-	-
BIC	2016	0.01	0.00	0.30	8.41	0.04	0.02	0.00	0.76	2.57	0.09	0.24
BIC	2017	-	-	-	8.46	0.04	-	-	-	2.44	-	-
BIC	2018	0.16	(0.00)	(0.01)	8.54	0.04	0.39	(0.00)	(0.02)	2.42	0.05	0.13
BIC	2019	-	-	-	8.61	0.05	-	-	-	2.99	-	-
BIC	2020	(0.05)	(0.00)	(0.29)	8.67	0.06	(0.18)	(0.00)	(1.06)	3.66	(0.10)	(0.38)
BIC	2021	(0.06)	0.00	(0.18)	8.71	0.08	(0.30)	0.01	(0.96)	5.32	(0.07)	(0.39)

**VNR**	2011	-	-	-	8.31	0.09	-	-	-	4.60	-	-
**VNR**	2012	0.01	0.00	0.23	8.35	0.07	0.03	0.00	1.04	4.47	0.07	0.33
**VNR**	2013	-	-	-	8.43	0.08	-	-	-	4.32	-	-
**VNR**	2014	0.40	(0.00)	(0.05)	8.71	0.07	1.65	(0.01)	(0.21)	4.12	0.13	0.53
**VNR**	2015	-	-	-	8.77	0.05	-	-	-	3.65	-	-
**VNR**	2016	(0.13)	(0.00)	0.17	8.76	0.05	(0.46)	(0.00)	0.58	3.47	0.00	0.01
**VNR**	2017	-	-	-	8.77	0.05	-	-	-	3.97	-	-
**VNR**	2018	(0.14)	(0.00)	0.07	8.81	0.05	(0.51)	(0.00)	0.27	3.76	(0.03)	(0.10)
**VNR**	2019	-	-	-	8.85	0.05	-	-	-	2.44	-	-
**VNR**	2020	(0.03)	0.00	(0.02)	8.85	0.05	(0.11)	0.00	(0.07)	3.20	(0.02)	(0.06)
**VNR**	2021	(0.04)	(0.00)	0.10	8.88	0.06	(0.12)	(0.00)	0.30	2.92	0.02	0.05
**MIG**	2011	-	-	-	6.78	0.06	-	-	-	2.02	-	-
**MIG**	2012	0.29	0.01	3.50	6.88	0.03	0.50	0.02	6.14	1.75	1.16	2.04
**MIG**	2013	-	-	-	7.20	0.03	-	-	-	2.26	-	-
**MIG**	2014	0.66	0.12	1.30	7.61	0.02	1.54	0.28	3.03	2.33	0.67	1.56
**MIG**	2015	-	-	-	7.72	0.02	-	-	-	5.39	-	-
**MIG**	2016	(0.59)	0.09	(0.77)	7.99	0.03	(1.27)	0.20	(1.66)	2.17	(0.41)	(0.89)
**MIG**	2017	-	-	-	8.01	0.02	-	-	-	2.54	-	-
**MIG**	2018	0.34	(0.02)	(4.30)	8.21	0.04	0.98	(0.05)	(12.47)	2.90	(1.18)	(3.44)
**MIG**	2019	-	-	-	8.50	0.04	-	-	-	3.04	-	-
**MIG**	2020	0.30	(0.01)	(0.62)	8.62	0.04	1.12	(0.04)	(2.34)	3.79	(0.08)	(0.32)
**MIG**	2021	0.14	(0.01)	(0.09)	8.79	0.04	0.76	(0.06)	(0.46)	5.32	0.02	0.11
**VIC**	2011	-	-	-	10.48	0.04	-	-	-	5.42	-	-
**VIC**	2012	(0.58)	0.01	(0.07)	10.93	0.05	(2.22)	0.04	(0.26)	3.80	(0.23)	(0.86)
**VIC**	2013	-	-	-	11.24	0.13	-	-	-	2.54	-	-
**VIC**	2014	(0.85)	0.09	0.79	11.41	0.06	(3.12)	0.34	2.89	3.67	(0.04)	(0.13)
**VIC**	2015	-	-	-	11.89	0.02	-	-	-	3.64	-	-
**VIC**	2016	0.11	0.01	(0.53)	12.10	0.03	0.41	0.05	(1.89)	3.58	(0.11)	(0.41)
**VIC**	2017	-	-	-	12.27	0.04	-	-	-	2.89	-	-
**VIC**	2018	(1.16)	0.00	(0.45)	12.57	0.05	(3.43)	0.01	(1.32)	2.96	(0.55)	(1.62)
**VIC**	2019	-	-	-	12.91	0.04	-	-	-	3.35	-	-
**VIC**	2020	(0.24)	0.03	0.15	12.95	0.03	(0.88)	0.11	0.57	3.70	(0.03)	(0.11)
**VIC**	2021	(0.43)	0.01	11.05	12.97	0.01	(1.54)	0.03	39.99	3.62	3.19	11.54
**SDI**	2011	-	-	-	8.94	0.02	-	-	-	3.69	-	-

**SDI**	2012	(5.46)	(0.02)	0.35	9.32	0.06	(20.00)	(0.06)	1.26	3.66	(1.86)	(6.82)
**SDI**	2013	-	-	-	9.44	0.12	-	-	-	3.55	-	-
**SDI**	2014	(0.82)	0.17	0.01	9.24	0.18	(2.89)	0.59	0.05	3.54	(0.23)	(0.82)
**SDI**	2015	-	-	-	8.59	0.10	-	-	-	3.63	-	-
**SDI**	2016	1.01	(0.09)	(0.19)	9.24	0.09	4.44	(0.39)	(0.82)	4.38	0.28	1.22
**SDI**	2017	-	-	-	10.04	0.12	-	-	-	1.01	-	-
**SDI**	2018	(0.76)	0.00	0.67	10.02	0.03	(1.97)	0.00	1.75	2.60	(0.07)	(0.18)
**SDI**	2019	#DIV/0!	#DIV/0!	#DIV/0!	#NUM!	#DIV/0!	#DIV/0!	#DIV/0!	#DIV/0!	3.08	#DIV/0!	#DIV/0!
**SDI**	2020	#DIV/0!	#DIV/0!	#DIV/0!	#NUM!	#DIV/0!	#DIV/0!	#DIV/0!	#DIV/0!	2.58	#DIV/0!	#DIV/0!
**SDI**	2021	#DIV/0!	#DIV/0!	#DIV/0!	#NUM!	#DIV/0!	#DIV/0!	#DIV/0!	#DIV/0!	2.75	#DIV/0!	#DIV/0!
**REE**	2011	-	-	-	8.57	0.12	-	-	-	3.32	-	-
**REE**	2012	0.19	(0.00)	(0.03)	8.79	0.12	0.66	(0.00)	(0.12)	3.51	0.06	0.20
**REE**	2013	-	-	-	8.85	0.16	-	-	-	3.43	-	-
**REE**	2014	0.01	0.06	(0.10)	9.04	0.15	0.06	0.28	(0.48)	4.62	(0.01)	(0.03)
**REE**	2015	-	-	-	9.17	0.11	-	-	-	2.24	-	-
**REE**	2016	0.04	(0.00)	(0.03)	9.34	0.12	0.09	(0.01)	(0.07)	2.46	0.00	0.01
**REE**	2017	-	-	-	9.57	0.12	-	-	-	2.45	-	-
**REE**	2018	(0.08)	(0.01)	(0.16)	9.65	0.14	(0.20)	(0.01)	(0.37)	2.38	(0.08)	(0.19)
**REE**	2019	-	-	-	9.88	0.10	-	-	-	2.48	-	-
**REE**	2020	(0.09)	0.03	0.08	9.93	0.09	(0.24)	0.09	0.23	2.81	0.00	0.01
**REE**	2021	0.26	0.38	0.05	10.37	0.08	1.03	1.47	0.18	3.90	0.24	0.92
**PDR**	2011	-	-	-	8.44	0.00	-	-	-	1.39	-	-
**PDR**	2012	0.38	(0.00)	0.33	8.55	0.00	0.93	(0.01)	0.83	2.49	0.23	0.58
**PDR**	2013	-	-	-	8.64	0.00	-	-	-	1.55	-	-
**PDR**	2014	0.16	(0.00)	(6.75)	8.71	0.01	0.38	(0.00)	(16.12)	2.39	(1.98)	(4.73)
**PDR**	2015	-	-	-	8.93	0.03	-	-	-	2.39	-	-
**PDR**	2016	0.42	0.00	0.10	9.11	0.03	0.95	0.00	0.22	2.25	0.18	0.41
**PDR**	2017	-	-	-	9.21	0.06	-	-	-	2.82	-	-
**PDR**	2018	(0.37)	0.00	(0.13)	9.31	0.07	(1.32)	0.00	(0.46)	3.57	(0.17)	(0.61)
**PDR**	2019	-	-	-	9.54	0.08	-	-	-	3.81	-	-
**PDR**	2020	(0.19)	0.00	(0.02)	9.66	0.10	(0.81)	0.00	(0.07)	4.31	(0.07)	(0.31)
**PDR**	2021	(0.48)	0.00	(0.01)	9.93	0.11	(1.46)	0.01	(0.03)	3.03	(0.17)	(0.53)
**DXG**	2011	-	-	-	6.79	0.06	-	-	-	2.92	-	-
**DXG**	2012	(0.64)	(0.00)	(1.41)	6.70	0.10	(1.54)	(0.00)	(3.36)	2.39	(0.66)	(1.57)

**DXG**	2013	-	-	-	7.15	0.09	-	-	-	2.45	-	-
**DXG**	2014	0.13	(0.00)	(0.68)	7.68	0.12	0.42	(0.02)	(2.20)	3.23	(0.16)	(0.52)
**DXG**	2015	-	-	-	8.18	0.15	-	-	-	2.89	-	-
**DXG**	2016	0.76	0.00	0.03	8.62	0.15	2.54	0.00	0.08	3.33	0.28	0.94
**DXG**	2017	-	-	-	9.24	0.13	-	-	-	1.42	-	-
**DXG**	2018	0.01	#DIV/0!	0.01	#NUM!	#DIV/0!	0.02	#DIV/0!	0.02	2.63	#DIV/0!	#DIV/0!
**DXG**	2019	-	-	-	9.90	0.12	-	-	-	3.43	-	-
**DXG**	2020	0.41	(0.00)	71.39	10.06	0.00	1.48	(0.01)	256.17	3.59	21.73	77.97
**DXG**	2021	(0.45)	(0.00)	(70.53)	10.25	0.09	(1.62)	(0.00)	(250.89)	3.56	(21.48)	(76.42)
**NLG**	2011	-	-	-	7.97	0.07	-	-	-	3.77	-	-
**NLG**	2012	0.07	0.00	1.55	8.03	0.02	0.23	0.00	5.30	3.41	0.49	1.69
**NLG**	2013	-	-	-	8.11	0.02	-	-	-	3.82	-	-
**NLG**	2014	(0.35)	(0.00)	(1.77)	8.28	0.04	(1.37)	(0.01)	(6.96)	3.94	(0.66)	(2.60)
**NLG**	2015	-	-	-	8.52	0.05	-	-	-	4.48	-	-
**NLG**	2016	(0.10)	(0.00)	(0.28)	8.73	0.08	(0.37)	(0.00)	(1.08)	3.84	(0.12)	(0.46)
**NLG**	2017	-	-	-	8.98	0.12	-	-	-	3.38	-	-
**NLG**	2018	(0.31)	(0.00)	0.00	9.17	0.12	(0.87)	(0.00)	0.00	2.81	(0.11)	(0.31)
**NLG**	2019	-	-	-	9.30	0.11	-	-	-	3.22	-	-
**NLG**	2020	0.27	(0.00)	0.17	9.52	0.07	0.75	(0.00)	0.46	2.77	0.15	0.41
**NLG**	2021	(0.28)	(0.00)	0.15	10.07	0.07	(1.48)	(0.01)	0.76	5.20	(0.06)	(0.31)
**KBC**	2011	-	-	-	9.39	0.01	-	-	-	4.73	-	-
**KBC**	2012	(13.28)	0.00	(3.89)	9.37	0.04	(49.52)	0.01	(14.52)	3.73	(5.95)	(22.16)
**KBC**	2013	-	-	-	9.44	0.01	-	-	-	3.08	-	-
**KBC**	2014	0.66	(0.00)	(4.04)	9.48	0.03	2.62	(0.01)	(16.17)	4.00	(0.99)	(3.95)
**KBC**	2015	-	-	-	9.52	0.05	-	-	-	5.26	-	-
**KBC**	2016	0.00	(0.00)	0.04	9.59	0.06	0.00	(0.01)	0.19	4.45	0.01	0.06
**KBC**	2017	-	-	-	9.67	0.05	-	-	-	5.31	-	-
**KBC**	2018	(0.03)	0.00	0.13	9.74	0.06	(0.07)	0.00	0.34	2.71	0.03	0.08
**KBC**	2019	-	-	-	9.71	0.08	-	-	-	2.57	-	-
**KBC**	2020	0.65	(0.00)	0.82	10.08	0.02	2.24	(0.02)	2.84	3.44	0.48	1.65
**KBC**	2021	(0.34)	(0.00)	(0.37)	10.33	0.04	(0.96)	(0.01)	(1.04)	2.82	(0.23)	(0.66)
**DIG**	2011	-	-	-	8.48	0.03	-	-	-	2.83	-	-
**DIG**	2012	0.07	(0.01)	23.80	8.54	0.00	0.20	(0.02)	64.90	2.73	7.22	19.68
**DIG**	2013	-	-	-	8.44	0.01	-	-	-	2.65	-	-

**DIG**	2014	0.14	(0.01)	(1.39)	8.52	0.01	0.37	(0.03)	(3.77)	2.71	(0.37)	(1.01)
**DIG**	2015	-	-	-	8.53	0.01	-	-	-	3.70	-	-
**DIG**	2016	0.18	(0.00)	(3.21)	8.68	0.02	0.53	(0.01)	(9.21)	2.87	(0.91)	(2.60)
**DIG**	2017	-	-	-	8.71	0.04	-	-	-	2.79	-	-
**DIG**	2018	0.02	0.01	(0.11)	8.83	0.06	0.07	0.05	(0.41)	3.63	(0.02)	(0.08)
**DIG**	2019	-	-	-	9.01	0.06	-	-	-	3.35	-	-
**DIG**	2020	0.42	0.02	(0.23)	9.38	0.08	1.62	0.09	(0.87)	3.83	0.09	0.35
**DIG**	2021	(0.27)	(0.02)	(0.03)	9.73	0.08	(1.50)	(0.12)	(0.18)	5.51	(0.12)	(0.63)
**HDG**	2011	-	-	-	7.40	0.13	-	-	-	4.95	-	-
**HDG**	2012	0.58	0.03	1.24	7.51	0.03	2.85	0.13	6.14	4.95	0.59	2.92
**HDG**	2013	-	-	-	7.75	0.08	-	-	-	3.04	-	-
**HDG**	2014	(0.52)	0.04	(0.14)	7.74	0.09	(1.68)	0.12	(0.46)	3.21	(0.22)	(0.71)
**HDG**	2015	-	-	-	8.02	0.06	-	-	-	5.80	-	-
**HDG**	2016	1.32	0.14	0.76	8.81	0.04	4.21	0.44	2.43	3.20	0.75	2.39
**HDG**	2017	-	-	-	9.04	0.04	-	-	-	4.50	-	-
**HDG**	2018	0.08	0.07	(1.13)	9.31	0.09	0.45	0.38	(6.21)	5.51	(0.29)	(1.59)
**HDG**	2019	-	-	-	9.54	0.10	-	-	-	5.63	-	-
**HDG**	2020	(0.74)	0.06	0.00	9.54	0.11	(2.40)	0.19	0.01	3.25	(0.24)	(0.79)
**HDG**	2021	(0.54)	0.32	0.08	9.67	0.10	(1.13)	0.67	0.17	2.08	(0.06)	(0.13)
**FLC**	2011	-	-	-	6.11	0.04	-	-	-	2.10	-	-
**FLC**	2012	(0.26)	0.08	0.91	7.66	0.02	(0.45)	0.14	1.60	1.77	0.21	0.37
**FLC**	2013	-	-	-	7.65	0.07	-	-	-	1.87	-	-
**FLC**	2014	(0.29)	(0.02)	(0.24)	8.59	0.08	(0.57)	(0.03)	(0.48)	1.98	(0.18)	(0.36)
**FLC**	2015	-	-	-	9.19	0.12	-	-	-	1.64	-	-
**FLC**	2016	0.56	(0.04)	0.48	9.79	0.07	1.31	(0.10)	1.12	2.36	0.33	0.78
**FLC**	2017	-	-	-	10.03	0.02	-	-	-	2.53	-	-
**FLC**	2018	0.18	(0.01)	0.06	10.16	0.03	0.47	(0.04)	0.16	2.68	0.08	0.21
**FLC**	2019	-	-	-	10.37	0.02	-	-	-	1.88	-	-
**FLC**	2020	(0.79)	(0.02)	1.80	10.54	0.01	(1.89)	(0.04)	4.31	2.39	0.26	0.61
**FLC**	2021	#DIV/0!	#DIV/0!	#DIV/0!	#NUM!	#DIV/0!	#DIV/0!	#DIV/0!	#DIV/0!	2.10	#DIV/0!	#DIV/0!
**ITA**	2011	-	-	-	9.11	0.01	-	-	-	2.19	-	-
**ITA**	2012	0.15	(0.00)	8.88	9.21	0.00	0.36	(0.00)	21.11	2.38	2.74	6.51
**ITA**	2013	-	-	-	9.28	0.01	-	-	-	2.46	-	-
**ITA**	2014	(0.00)	(0.00)	(2.09)	9.39	0.01	(0.00)	(0.01)	(4.63)	2.21	(0.63)	(1.40)

**ITA**	2015	-	-	-	9.45	0.01	-	-	-	2.06	-	-
**ITA**	2016	(0.12)	(0.00)	1.34	9.47	0.00	(0.22)	(0.00)	2.44	1.81	0.36	0.66
**ITA**	2017	-	-	-	9.47	0.00	-	-	-	1.86	-	-
**ITA**	2018	0.01	(0.00)	(10.77)	9.48	0.01	0.01	(0.00)	(25.97)	2.41	(3.25)	(7.85)
**ITA**	2019	-	-	-	9.50	0.02	-	-	-	1.85	-	-
**ITA**	2020	(0.00)	0.00	(0.16)	9.51	0.02	(0.01)	0.00	(0.33)	2.07	(0.05)	(0.10)
**ITA**	2021	(0.05)	0.00	(0.39)	9.49	0.02	(0.06)	0.00	(0.47)	1.21	(0.13)	(0.16)
**IJC**	2011	-	-	-	8.39	0.09	-	-	-	5.54	-	-
**IJC**	2012	0.13	(0.01)	0.25	8.44	0.05	0.61	(0.05)	1.14	4.52	0.12	0.54
**IJC**	2013	-	-	-	8.48	0.04	-	-	-	4.60	-	-
**IJC**	2014	0.60	(0.04)	(0.18)	8.81	0.04	3.07	(0.21)	(0.90)	5.14	0.15	0.75
**IJC**	2015	-	-	-	8.90	0.02	-	-	-	3.42	-	-
**IJC**	2016	0.60	(0.02)	0.28	9.12	0.02	2.02	(0.07)	0.93	3.37	0.29	0.99
**IJC**	2017	-	-	-	8.99	0.03	-	-	-	3.71	-	-
**IJC**	2018	(0.15)	(0.00)	(0.21)	9.00	0.03	(0.51)	(0.02)	(0.73)	3.47	(0.12)	(0.41)
**IJC**	2019	-	-	-	8.93	0.05	-	-	-	4.06	-	-
**IJC**	2020	(0.91)	0.01	(0.11)	8.80	0.07	(1.56)	0.02	(0.18)	1.72	(0.35)	(0.61)
**IJC**	2021	(1.17)	(0.01)	(0.20)	8.83	0.11	(3.57)	(0.02)	(0.62)	3.04	(0.48)	(1.48)
**NBB**	2011	-	-	-	7.84	0.04	-	-	-	1.45	-	-
**NBB**	2012	(0.20)	0.01	(0.27)	7.97	0.08	(0.96)	0.02	(1.25)	4.73	(0.15)	(0.72)
**NBB**	2013	-	-	-	8.03	0.01	-	-	-	1.94	-	-
**NBB**	2014	(0.36)	(0.02)	(0.33)	8.05	0.02	(0.66)	(0.03)	(0.60)	1.82	(0.24)	(0.43)
**NBB**	2015	-	-	-	8.17	0.01	-	-	-	3.46	-	-
**NBB**	2016	0.79	(0.03)	0.49	8.51	0.01	0.96	(0.03)	0.59	1.21	0.42	0.51
**NBB**	2017	-	-	-	8.51	0.02	-	-	-	1.19	-	-
**NBB**	2018	(0.12)	(0.00)	(0.96)	8.54	0.04	(0.14)	(0.00)	(1.15)	1.20	(0.33)	(0.40)
**NBB**	2019	-	-	-	8.64	0.08	-	-	-	1.19	-	-
**NBB**	2020	(0.31)	0.03	0.53	8.32	0.11	(1.82)	0.15	3.11	5.91	0.06	0.34
**NBB**	2021	(0.12)	(0.01)	(0.24)	8.38	0.10	(0.74)	(0.04)	(1.44)	5.99	(0.12)	(0.71)
**SCR**	2011	-	-	-	8.82	0.00	-	-	-	5.08	-	-
**SCR**	2012	(0.22)	0.00	(30.31)	8.79	0.02	(1.11)	0.00	(149.78)	4.94	(9.24)	(45.68)
**SCR**	2013	-	-	-	8.63	0.01	-	-	-	2.88	-	-
**SCR**	2014	(0.12)	(0.00)	(0.10)	8.59	0.01	(0.29)	(0.00)	(0.24)	2.48	(0.07)	(0.18)
**SCR**	2015	-	-	-	8.52	0.04	-	-	-	1.87	-	-

**SCR**	2016	0.65	0.02	(1.02)	8.92	0.03	3.70	0.09	(5.80)	5.67	(0.07)	(0.39)
**SCR**	2017	-	-	-	9.16	0.03	-	-	-	4.58	-	-
**SCR**	2018	(0.32)	0.05	0.11	9.29	0.03	(1.91)	0.27	0.66	5.90	(0.07)	(0.39)
**SCR**	2019	-	-	-	9.30	0.03	-	-	-	4.68	-	-
**SCR**	2020	0.04	(0.00)	1.39	9.34	0.02	0.21	(0.01)	8.22	5.90	0.43	2.56
**SCR**	2021	(0.39)	0.00	0.42	9.19	0.02	(1.02)	0.01	1.10	2.63	(0.01)	(0.03)
**D2D**	2011	-	-	-	6.58	0.08	-	-	-	2.96	-	-
**D2D**	2012	0.31	(0.42)	0.02	6.73	0.07	1.07	(1.45)	0.07	3.41	(0.03)	(0.09)
**D2D**	2013	-	-	-	7.01	0.06	-	-	-	3.88	-	-
**D2D**	2014	(0.23)	0.03	(0.07)	7.02	0.06	(1.10)	0.16	(0.34)	4.75	(0.09)	(0.45)
**D2D**	2015	-	-	-	7.01	0.06	-	-	-	4.76	-	-
**D2D**	2016	0.55	(0.00)	(0.14)	7.23	0.05	2.83	(0.00)	(0.74)	5.15	0.15	0.79
**D2D**	2017	-	-	-	7.13	0.08	-	-	-	3.05	-	-
**D2D**	2018	0.43	(0.00)	(0.01)	7.37	0.07	1.67	(0.01)	(0.05)	3.93	0.15	0.58
**D2D**	2019	-	-	-	7.65	0.22	-	-	-	2.68	-	-
**D2D**	2020	(0.57)	0.00	0.03	7.60	0.17	(1.56)	0.00	0.08	2.75	(0.19)	(0.53)
**D2D**	2021	(0.44)	(0.00)	0.03	7.49	0.17	(1.55)	(0.00)	0.09	3.52	(0.15)	(0.53)
**SJS**	2011	-	-	-	8.52	0.02	-	-	-	4.42	-	-
**SJS**	2012	0.88	(0.70)	(0.79)	8.61	0.05	4.99	(3.97)	(4.49)	5.69	(0.16)	(0.91)
**SJS**	2013	-	-	-	8.63	0.01	-	-	-	3.84	-	-
**SJS**	2014	(0.41)	(0.02)	(1.81)	8.60	0.04	(1.83)	(0.10)	(8.12)	4.49	(0.70)	(3.14)
**SJS**	2015	-	-	-	8.65	0.05	-	-	-	4.63	-	-
**SJS**	2016	(0.06)	(0.00)	(0.20)	8.69	0.04	-	-	-		(0.08)	-
**SJS**	2017	-	-	-	8.75	0.03	-	-	-	3.71	-	-
**SJS**	2018	0.10	0.03	(0.01)	8.77	0.02	0.58	0.16	(0.07)	5.59	0.04	0.24
**SJS**	2019	-	-	-	8.80	0.02	-	-	-	4.98	-	-
**SJS**	2020	0.30	(0.00)	0.39	8.86	0.02	1.66	(0.02)	2.16	5.60	0.22	1.24
**SJS**	2021	(0.11)	(0.00)	0.02	8.85	0.02	(0.51)	(0.00)	0.08	4.50	(0.04)	(0.16)
**IDI**	2011	-	-	-	7.29	0.03	-	-	-	4.58	-	-
**IDI**	2012	0.05	(0.01)	0.79	7.29	0.02	0.16	(0.03)	2.59	3.27	0.25	0.83
**IDI**	2013	-	-	-	7.52	0.02	-	-	-	1.70	-	-
**IDI**	2014	(0.09)	(0.02)	(2.23)	7.58	0.05	(0.20)	(0.04)	(5.08)	2.28	(0.71)	(1.62)
**IDI**	2015	-	-	-	8.15	0.03	-	-	-	2.78	-	-
**IDI**	2016	(0.49)	(0.10)	0.50	8.53	0.02	(1.02)	(0.21)	1.04	2.09	(0.06)	(0.12)

**IDI**	2017	-	-	-	8.65	0.06	-	-	-	2.16	-	-
**IDI**	2018	(0.10)	0.03	(0.51)	8.80	0.11	(0.55)	0.17	(2.83)	5.56	(0.18)	(0.99)
**IDI**	2019	-	-	-	8.92	0.05	-	-	-	5.30	-	-
**IDI**	2020	(0.02)	(0.01)	2.74	8.95	0.02	(0.06)	(0.02)	8.14	2.97	0.82	2.43
**IDI**	2021	(0.17)	(0.01)	(1.41)	8.93	0.02	(0.26)	(0.01)	(2.16)	1.53	(0.49)	(0.75)
**SZL**	2011	-	-	-	6.89	0.06	-	-	-	2.20	-	-
**SZL**	2012	0.01	0.02	0.78	6.99	0.05	0.03	0.05	1.88	2.41	0.25	0.59
**SZL**	2013	-	-	-	7.04	0.04	-	-	-	2.16	-	-
**SZL**	2014	0.25	(0.02)	(0.02)	7.12	0.04	0.64	(0.04)	(0.05)	2.55	0.08	0.20
**SZL**	2015	-	-	-	7.16	0.04	-	-	-	2.52	-	-
**SZL**	2016	(0.10)	(0.03)	(0.23)	7.32	0.08	(0.41)	(0.13)	(0.97)	4.14	(0.12)	(0.48)
**SZL**	2017	-	-	-	7.32	0.07	-	-	-	1.08	-	-
**SZL**	2018	(0.11)	0.00	(0.01)	7.38	0.08	(0.11)	0.00	(0.01)	1.05	(0.04)	(0.04)
**SZL**	2019	-	-	-	7.40	0.08	-	-	-	1.09	-	-
**SZL**	2020	0.67	(0.01)	0.02	7.41	0.08	0.74	(0.01)	0.02	1.10	0.24	0.27
**SZL**	2021	(0.14)	(0.01)	0.00	7.43	0.07	(0.15)	(0.01)	0.00	1.14	(0.05)	(0.06)
**TDH**	2011	-	-	-	7.73	0.02	-	-	-	1.22	-	-
**TDH**	2012	(0.05)	(0.01)	(1.15)	7.70	0.01	(0.23)	(0.07)	(5.67)	4.94	(0.37)	(1.82)
**TDH**	2013	-	-	-	7.72	0.01	-	-	-	4.64	-	-
**TDH**	2014	0.06	0.03	(2.45)	7.80	0.02	0.06	0.03	(2.52)	1.03	(0.71)	(0.73)
**TDH**	2015	-	-	-	7.84	0.02	-	-	-	1.25	-	-
**TDH**	2016	(0.25)	(0.00)	(2.68)	7.94	0.05	(0.26)	(0.00)	(2.87)	1.07	(0.90)	(0.96)
**TDH**	2017	-	-	-	7.96	0.06	-	-	-	1.03	-	-
**TDH**	2018	0.28	0.08	0.90	8.43	0.03	0.93	0.26	3.04	3.37	0.40	1.34
**TDH**	2019	-	-	-	8.58	0.04	-	-	-	2.66	-	-
**TDH**	2020	0.41	(0.01)	0.95	8.63	0.05	0.94	(0.02)	2.19	2.30	0.43	0.99
**TDH**	2021	0.48	(0.08)	(1.31)	7.52	0.47	2.27	(0.37)	(6.24)	4.78	(0.25)	(1.20)
**TDC**	2011	-	-	-	7.90	0.09	-	-	-	4.68	-	-
**TDC**	2012	0.71	0.02	0.36	8.16	0.06	3.94	0.13	1.99	5.51	0.37	2.06
**TDC**	2013	-	-	-	8.39	0.04	-	-	-	3.13	-	-
**TDC**	2014	1.69	(0.01)	0.32	8.74	0.02	8.78	(0.07)	1.67	5.19	0.70	3.63
**TDC**	2015	-	-	-	8.90	0.02	-	-	-	4.71	-	-
**TDC**	2016	(0.19)	(0.05)	(0.35)	8.89	0.02	(0.82)	(0.22)	(1.49)	4.27	(0.19)	(0.82)
**TDC**	2017	-	-	-	8.97	0.02	-	-	-	4.42	-	-

**TDC**	2018	(0.88)	(0.00)	0.14	8.82	0.02	(3.94)	(0.01)	0.63	4.50	(0.27)	(1.23)
**TDC**	2019	-	-	-	8.74	0.03	-	-	-	1.09	-	-
**TDC**	2020	(0.63)	0.00	(0.70)	8.64	0.05	(0.69)	0.00	(0.77)	1.10	(0.44)	(0.48)
**TDC**	2021	(0.32)	0.00	0.53	8.56	0.03	(0.35)	0.00	0.59	1.12	0.05	0.05
**LHG**	2011	-	-	-	7.51	0.07	-	-	-	1.08	-	-
**LHG**	2012	(0.35)	(0.04)	0.82	7.42	0.04	(1.29)	(0.16)	3.00	3.65	0.11	0.39
**LHG**	2013	-	-	-	7.41	0.03	-	-	-	6.00	-	-
**LHG**	2014	(0.25)	(0.01)	0.77	7.30	0.03	(1.32)	(0.06)	3.99	5.20	0.14	0.71
**LHG**	2015	-	-	-	7.23	0.02	-	-	-	5.50	-	-
**LHG**	2016	0.26	(0.01)	(1.68)	7.33	0.13	0.71	(0.02)	(4.58)	2.73	(0.42)	(1.13)
**LHG**	2017	-	-	-	7.59	0.10	-	-	-	1.37	-	-
**LHG**	2018	0.02	(0.00)	(0.06)	7.66	0.10	0.05	(0.01)	(0.13)	2.10	(0.01)	(0.02)
**LHG**	2019	-	-	-	7.71	0.08	-	-	-	1.78	-	-
**LHG**	2020	0.15	0.00	(0.03)	7.86	0.09	0.81	0.02	(0.18)	5.44	0.04	0.24
**LHG**	2021	(0.08)	(0.00)	(0.17)	7.95	0.13	(0.44)	(0.02)	(0.92)	5.39	(0.08)	(0.44)
**IDV**	2011	-	-	-	5.68	0.05	-	-	-	4.73	-	-
**IDV**	2012	0.75	(0.01)	(0.44)	5.62	0.03	3.50	(0.04)	(2.08)	4.68	0.13	0.61
**IDV**	2013	-	-	-	5.76	0.06	-	-	-	3.41	-	-
**IDV**	2014	(1.39)	(0.16)	(0.51)	6.03	0.12	(4.99)	(0.59)	(1.82)	3.60	(0.71)	(2.54)
**IDV**	2015	-	-	-	6.22	0.11	-	-	-	3.16	-	-
**IDV**	2016	(0.31)	(0.06)	0.01	6.43	0.13	(0.97)	(0.19)	0.04	3.12	(0.13)	(0.40)
**IDV**	2017	-	-	-	6.56	0.13	-	-	-	2.77	-	-
**IDV**	2018	(0.23)	(0.01)	0.01	6.63	0.10	(0.57)	(0.02)	0.02	2.49	(0.08)	(0.21)
**IDV**	2019	-	-	-	6.83	0.12	-	-	-	4.57	-	-
**IDV**	2020	(0.44)	0.01	(0.03)	7.14	0.19	(2.09)	0.06	(0.16)	4.79	(0.16)	(0.78)
**IDV**	2021	(0.42)	(0.01)	(0.01)	7.25	0.12	-	-	-		(0.16)	-
**HQC**	2011	-	-	-	7.93	0.02	-	-	-		-	-
**HQC**	2012	(0.23)	0.00	0.17	7.91	0.01	(1.10)	0.00	0.83	4.86	(0.03)	(0.14)
**HQC**	2013	-	-	-	8.05	0.01	-	-	-	5.88	-	-
**HQC**	2014	(1.01)	(0.00)	(1.01)	8.31	0.01	(4.84)	(0.00)	(4.84)	4.77	(0.67)	(3.20)
**HQC**	2015	-	-	-	8.76	0.11	-	-	-	3.03	-	-
**HQC**	2016	0.10	0.00	9.02	8.83	0.00	0.60	0.01	53.96	5.98	2.76	16.53
**HQC**	2017	-	-	-	8.75	0.01	-	-	-	5.10	-	-
**HQC**	2018	0.06	(0.00)	1.31	8.79	0.01	-	-	-		0.41	-

**HQC**	2019	-	-	-	8.81	0.01	-	-	-		-	-
**HQC**	2020	0.06	(0.00)	7.44	8.85	0.00	0.15	(0.00)	20.16	2.71	2.27	6.15
**HQC**	2021	0.54	(0.00)	1.48	9.14	0.00	2.46	(0.00)	6.74	4.55	0.64	2.92
**DLG**	2011	-	-	-	7.56	0.02	-	-	-	5.19	-	-
**DLG**	2012	(1.66)	(0.01)	26.71	7.69	0.00	(6.53)	(0.04)	105.00	3.93	7.48	29.38
**DLG**	2013	-	-	-	7.79	0.00	-	-	-	4.00	-	-
**DLG**	2014	(0.30)	0.08	(16.15)	8.32	0.01	(1.18)	0.33	(63.69)	3.94	(4.96)	(19.57)
**DLG**	2015	-	-	-	8.83	0.01	-	-	-	5.53	-	-
**DLG**	2016	(0.52)	(0.00)	1.92	8.86	0.01	(1.98)	(0.01)	7.27	3.78	0.39	1.48
**DLG**	2017	-	-	-	9.03	0.01	-	-	-	2.54	-	-
**DLG**	2018	0.02	0.02	8.87	9.07	0.00	0.05	0.05	20.04	2.26	2.70	6.09
**DLG**	2019	-	-	-	9.06	0.00	-	-	-	3.21	-	-
**DLG**	2020	0.77	0.05	(17.15)	9.02	0.10	2.37	0.16	(52.64)	3.07	(4.89)	(15.01)
**DLG**	2021	(0.23)	0.01	125.01	8.86	0.00	(0.91)	0.05	487.38	3.90	37.71	147.02
**LGL**	2011	-	-	-	6.72	0.01	-	-	-	4.87	-	-
**LGL**	2012	0.24	(0.12)	(0.40)	6.82	0.01	0.75	(0.36)	(1.24)	3.14	(0.07)	(0.23)
**LGL**	2013	-	-	-	6.60	0.06	-	-	-	2.69	-	-
**LGL**	2014	(0.32)	(0.04)	(0.64)	6.57	0.05	(0.75)	(0.10)	(1.47)	2.31	(0.32)	(0.75)
**LGL**	2015	-	-	-	6.71	0.02	-	-	-	2.83	-	-
**LGL**	2016	2.82	(0.02)	(1.84)	7.51	0.01	7.66	(0.06)	(4.99)	2.72	0.45	1.22
**LGL**	2017	-	-	-	7.71	0.06	-	-	-	2.16	-	-
**LGL**	2018	(1.10)	(0.00)	0.29	7.79	0.06	(3.26)	(0.01)	0.85	2.96	(0.31)	(0.92)
**LGL**	2019	-	-	-	7.48	0.04	-	-	-	4.27	-	-
**LGL**	2020	0.07	(0.00)	0.76	7.50	0.01	0.26	(0.01)	2.86	3.76	0.25	0.95
**LGL**	2021	(0.12)	(0.00)	2.59	7.44	0.01	(0.65)	(0.01)	13.49	5.21	0.74	3.84
**VPH**	2011	-	-	-	7.44	0.01	-	-	-	5.90	-	-
**VPH**	2012	0.12	(0.00)	(1.40)	7.49	0.00	0.68	(0.02)	(8.10)	5.78	(0.38)	(2.21)
**VPH**	2013	-	-	-	7.49	0.01	-	-	-	5.79	-	-
**VPH**	2014	(0.06)	(0.01)	4.36	7.45	0.00	(0.22)	(0.04)	17.05	3.91	1.29	5.06
**VPH**	2015	-	-	-	7.42	0.09	-	-	-	5.08	-	-
**VPH**	2016	(0.13)	0.00	0.07	7.47	0.06	(0.68)	0.00	0.37	5.42	(0.02)	(0.13)
**VPH**	2017	-	-	-	7.56	0.13	-	-	-	2.28	-	-
**VPH**	2018	(0.04)	(0.00)	0.54	7.64	0.09	(0.08)	(0.00)	1.01	1.88	0.15	0.28
**VPH**	2019	-	-	-	7.49	0.03	-	-	-	1.63	-	-

**VPH**	2020	0.26	(0.00)	1.89	7.64	0.01	0.51	(0.01)	3.75	1.98	0.66	1.31
**VPH**	2021	(0.09)	(0.00)	(2.42)	7.63	0.05	(0.17)	(0.00)	(4.45)	1.84	(0.76)	(1.41)
**UDC**	2011	-	-	-	7.44	0.01	-	-	-	2.84	-	-
**UDC**	2012	0.14	0.02	2.78	7.47	0.01	0.57	0.08	11.61	4.17	0.90	3.74
**UDC**	2013	-	-	-	7.50	0.00	-	-	-	3.48	-	-
**UDC**	2014	(0.19)	(0.03)	(2.00)	7.41	0.00	(0.80)	(0.13)	(8.49)	4.24	(0.68)	(2.90)
**UDC**	2015	-	-	-	7.49	0.01	-	-	-	4.71	-	-
**UDC**	2016	(1.78)	0.03	2.50	6.98	0.01	(7.02)	0.11	9.86	3.94	0.13	0.50
**UDC**	2017	-	-	-	7.05	0.01	-	-	-	3.25	-	-
**UDC**	2018	0.03	0.29	0.20	7.05	0.01	0.09	1.04	0.72	3.58	0.17	0.60
**UDC**	2019	-	-	-	6.97	0.01	-	-	-	2.42	-	-
**UDC**	2020	0.18	(0.03)	13.08	7.00	0.00	0.77	(0.11)	56.30	4.30	4.01	17.26
**UDC**	2021	0.04	0.00	(15.81)	6.96	0.02	0.18	0.00	(68.44)	4.33	(4.76)	(20.62)
**RCL**	2011	-	-	-	5.57	0.15	-	-	-	4.49	-	-
**RCL**	2012	(0.18)	0.00	(0.08)	5.56	0.12	(0.89)	0.00	(0.42)	5.04	(0.09)	(0.45)
**RCL**	2013	-	-	-	5.75	0.08	-	-	-	3.74	-	-
**RCL**	2014	(0.22)	0.00	0.04	5.68	0.09	(0.84)	0.00	0.16	3.87	(0.07)	(0.25)
**RCL**	2015	-	-	-	5.62	0.09	-	-	-	2.81	-	-
**RCL**	2016	(0.25)	0.82	0.18	5.63	0.05	(0.86)	2.80	0.61	3.40	0.24	0.82
**RCL**	2017	-	-	-	5.84	0.06	-	-	-	4.93	-	-
**RCL**	2018	0.92	(0.02)	0.19	6.30	0.03	4.22	(0.08)	0.86	4.60	0.38	1.75
**RCL**	2019	-	-	-	6.56	0.02	-	-	-	5.46	-	-
**RCL**	2020	(0.14)	0.00	0.19	6.50	0.02	(0.69)	0.02	0.93	4.98	0.01	0.04
**RCL**	2021	(0.49)	(0.01)	(0.51)	6.28	0.04	(2.86)	(0.03)	(2.96)	5.78	(0.33)	(1.93)
**NHA**	2011	-	-	-	4.72	0.27	-	-	-	6.00	-	-
**NHA**	2012	0.05	0.42	2.87	4.77	0.01	0.28	2.29	15.60	5.44	1.03	5.59
**NHA**	2013	-	-	-	4.78	0.01	-	-	-	5.22	-	-
**NHA**	2014	(0.04)	0.09	(3.57)	4.72	0.06	(0.21)	0.56	(21.43)	6.00	(1.06)	(6.37)
**NHA**	2015	-	-	-	5.21	0.09	-	-	-	4.76	-	-
**NHA**	2016	(0.39)	0.10	0.09	4.97	0.15	(1.89)	0.48	0.42	4.82	(0.08)	(0.39)
**NHA**	2017	-	-	-	5.06	0.19	-	-	-	4.35	-	-
**NHA**	2018	0.00	(0.12)	(0.03)	5.24	0.18	0.02	(0.72)	(0.16)	5.82	(0.05)	(0.28)
**NHA**	2019	-	-	-	5.59	0.32	-	-	-	5.16	-	-
**NHA**	2020	0.03	(0.05)	0.16	5.76	0.13	0.12	(0.20)	0.61	3.87	0.04	0.16

**NHA**	2021	0.66	(0.08)	3.40	6.23	0.01	3.94	(0.50)	20.42	6.00	1.24	7.42
**RCD**	2011						-	-	-	5.44	-	-
**RCD**	2012						-	-	-	5.81	-	-
**RCD**	2013	-	-	-	6.02	0.01	-	-	-	4.27	-	-
**RCD**	2014	0.39	-	(0.08)	5.97	0.01	1.22	-	(0.26)	3.15	0.11	0.36
**RCD**	2015	-	-	-	6.80	0.01	-	-	-	3.34	-	-
**RCD**	2016	(11.13)	-	(1.32)	5.97	0.36	(27.64)	-	(3.28)	2.48	(4.40)	(10.91)
**RCD**	2017	-	-	-	5.81	0.03	-	-	-	5.23	-	-
**RCD**	2018	0.10	-	7.11	5.52	0.00	0.19	-	13.16	1.85	2.19	4.05
**RCD**	2019	-	-	-	5.08	0.01	-	-	-	3.07	-	-
**RCD**	2020	0.02	-	(4.95)	4.56	0.41	0.07	-	(13.45)	2.72	(1.49)	(4.04)
**RCD**	2021	(0.02)	-	0.45	4.52	0.02	(0.06)	-	1.15	2.56	0.13	0.33
**TKC**	2011	-	-	-	5.88	0.03	-	-	-	1.34	-	-
**TKC**	2012	0.69	(0.00)	9.11	6.08	0.00	1.01	(0.00)	13.27	1.46	3.00	4.37
**TKC**	2013	-	-	-	5.86	0.01	-	-	-	2.88	-	-
**TKC**	2014	(0.24)	0.08	(2.00)	5.76	0.01	(0.47)	0.15	(3.84)	1.92	(0.67)	(1.28)
**TKC**	2015	-	-	-	6.26	0.02	-	-	-	1.65	-	-
**TKC**	2016	0.70	0.04	(1.05)	6.50	0.03	1.15	0.07	(1.70)	1.63	(0.05)	(0.08)
**TKC**	2017	-	-	-	7.01	0.03	-	-	-	1.28	-	-
**TKC**	2018	(1.86)	0.01	1.99	6.76	0.02	(4.06)	0.02	4.33	2.18	(0.06)	(0.14)
**TKC**	2019	-	-	-	6.52	0.01	-	-	-	2.58	-	-
**TKC**	2020	2.30	(0.04)	(6.20)	6.98	0.01	-	-	-		(1.06)	-
**TKC**	2021	2.51	(0.02)	12.75	7.33	0.00	11.52	(0.09)	58.49	4.59	4.75	21.79
**SSI**	2011	-	-	-	8.78	0.02	-	-	-	1.56	-	-
**SSI**	2012	0.26	(0.01)	(0.18)	8.98	0.06	0.61	(0.02)	(0.43)	2.37	0.03	0.08
**SSI**	2013	-	-	-	8.95	0.07	-	-	-	5.72	-	-
**SSI**	2014	0.10	(0.00)	(0.01)	9.10	0.10	0.50	(0.01)	(0.04)	5.10	0.03	0.16
**SSI**	2015	-	-	-	9.50	0.08	-	-	-	2.24	-	-
**SSI**	2016	(0.14)	0.00	0.24	9.49	0.08	(0.24)	0.00	0.41	1.70	0.02	0.04
**SSI**	2017	-	-	-	9.84	0.07	-	-	-	1.25	-	-
**SSI**	2018	0.42	(0.00)	0.09	10.08	0.07	0.84	(0.01)	0.18	1.98	0.18	0.35
**SSI**	2019	-	-	-	10.21	0.04	-	-	-	5.18	-	-
**SSI**	2020	0.75	(0.00)	(0.16)	10.48	0.04	3.66	(0.01)	(0.77)	4.90	0.22	1.08
**SSI**	2021	(0.05)	(0.00)	(0.27)	10.84	0.07	(0.23)	(0.00)	(1.20)	4.44	(0.10)	(0.44)

**HCM**	2011	-	-	-	7.87	0.09	-	-	-	5.47	-	-
**HCM**	2012	0.18	(0.00)	(0.07)	8.07	0.10	0.26	(0.00)	(0.10)	1.42	0.04	0.06
**HCM**	2013	-	-	-	8.05	0.12	-	-	-	1.25	-	-
**HCM**	2014	(0.00)	(0.00)	(0.04)	8.26	0.12	(0.00)	(0.00)	(0.07)	1.85	(0.01)	(0.02)
**HCM**	2015	-	-	-	8.19	0.08	-	-	-	1.53	-	-
**HCM**	2016	(0.07)	(0.00)	(0.11)	8.19	0.11	(0.10)	(0.00)	(0.17)	1.53	(0.06)	(0.09)
**HCM**	2017	-	-	-	8.81	0.10	-	-	-	1.68	-	-
**HCM**	2018	(0.68)	0.00	0.82	8.57	0.16	(1.23)	0.01	1.50	1.82	0.01	0.01
**HCM**	2019	-	-	-	8.92	0.07	-	-	-	1.93	-	-
**HCM**	2020	1.07	(0.00)	(0.02)	9.43	0.05	1.64	(0.00)	(0.04)	1.53	0.38	0.58
**HCM**	2021	0.51	(0.00)	(0.12)	10.10	0.06	1.50	(0.01)	(0.35)	2.91	0.15	0.43
**VND**	2011	-	-	-	7.31	0.14	-	-	-	2.10	-	-
**VND**	2012	0.05	0.00	0.49	7.40	0.05	0.10	0.00	0.97	1.98	0.17	0.33
**VND**	2013	-	-	-	7.57	0.07	-	-	-	2.99	-	-
**VND**	2014	(0.02)	(0.00)	0.04	7.99	0.07	(0.06)	(0.01)	0.11	2.63	0.00	0.01
**VND**	2015	-	-	-	8.66	0.04	-	-	-	2.02	-	-
**VND**	2016	(0.32)	(0.00)	0.69	8.54	0.04	(1.00)	(0.00)	2.15	3.13	0.09	0.29
**VND**	2017	-	-	-	8.99	0.07	-	-	-	1.59	-	-
**VND**	2018	0.32	(0.00)	0.61	9.26	0.04	0.44	(0.00)	0.82	1.36	0.30	0.41
**VND**	2019	-	-	-	9.36	0.04	-	-	-	1.25	-	-
**VND**	2020	0.36	(0.00)	(0.71)	9.61	0.06	0.46	(0.00)	(0.92)	1.29	(0.09)	(0.11)
**VND**	2021	(0.14)	(0.00)	(0.60)	10.52	0.08	(0.24)	(0.00)	(0.97)	1.62	(0.23)	(0.38)
**MBS**	2011	-	-	-	8.02	0.19	-	-	-	1.51	-	-
**MBS**	2012	(0.34)	(0.00)	10.20	7.93	0.01	(0.50)	(0.00)	15.10	1.48	2.96	4.38
**MBS**	2013	-	-	-	7.88	0.00	-	-	-	1.51	-	-
**MBS**	2014	0.18	(0.00)	(0.11)	8.03	0.02	0.51	(0.00)	(0.32)	2.81	0.03	0.09
**MBS**	2015	-	-	-	8.05	0.00	-	-	-	4.02	-	-
**MBS**	2016	0.26	0.00	0.25	8.17	0.00	0.53	0.00	0.52	2.03	0.17	0.35
**MBS**	2017	-	-	-	8.36	0.01	-	-	-	2.27	-	-
**MBS**	2018	(0.74)	0.00	(10.72)	8.24	0.05	(2.07)	0.01	(30.04)	2.80	(3.51)	(9.82)
**MBS**	2019	-	-	-	8.47	0.06	-	-	-	2.37	-	-
**MBS**	2020	0.56	(0.00)	(0.07)	8.86	0.05	1.95	(0.01)	(0.23)	3.47	0.18	0.63
**MBS**	2021	(0.17)	0.00	(0.10)	9.31	0.07	(0.51)	0.01	(0.31)	3.07	(0.09)	(0.28)
**SHS**	2011	-	-	-	7.07	0.32	-	-	-	2.79	-	-

**SHS**	2012	0.34	(0.01)	3.78	7.31	0.02	0.57	(0.01)	6.24	1.65	1.26	2.09
**SHS**	2013	-	-	-	7.56	0.01	-	-	-	1.50	-	-
**SHS**	2014	1.05	(0.00)	(2.22)	8.06	0.04	5.20	(0.01)	(11.01)	4.96	(0.29)	(1.46)
**SHS**	2015	-	-	-	8.12	0.04	-	-	-	5.28	-	-
**SHS**	2016	(0.29)	0.00	1.52	8.11	0.03	(1.59)	0.00	8.27	5.44	0.36	1.93
**SHS**	2017	-	-	-	8.33	0.11	-	-	-	6.00	-	-
**SHS**	2018	(0.21)	0.00	0.20	8.49	0.08	(1.09)	0.00	1.05	5.20	(0.01)	(0.07)
**SHS**	2019	-	-	-	8.71	0.05	-	-	-		-	-
**SHS**	2020	(0.11)	(0.00)	(0.67)	8.84	0.14	(0.11)	(0.00)	(0.67)	1.01	(0.24)	(0.24)
**SHS**	2021	(0.35)	0.00	(0.12)	9.30	0.16	(0.35)	0.00	(0.12)	1.01	(0.16)	(0.16)
**FTS**	2011	#DIV/0!	#DIV/0!	#DIV/0!	#NUM!	#DIV/0!	#DIV/0!	#DIV/0!	#DIV/0!	1.01	#DIV/0!	#DIV/0!
**FTS**	2012	#DIV/0!	#DIV/0!	#DIV/0!	#NUM!	#DIV/0!	#DIV/0!	#DIV/0!	#DIV/0!	2.12	#DIV/0!	#DIV/0!
**FTS**	2013	#DIV/0!	#DIV/0!	#DIV/0!	#NUM!	#DIV/0!	#DIV/0!	#DIV/0!	#DIV/0!	5.22	#DIV/0!	#DIV/0!
**FTS**	2014	#DIV/0!	#DIV/0!	#DIV/0!	7.68	0.08	#DIV/0!	#DIV/0!	#DIV/0!	1.75	#DIV/0!	#DIV/0!
**FTS**	2015	-	-	-	7.65	0.08	-	-	-	1.19	-	-
**FTS**	2016	(0.52)	0.01	(0.04)	7.31	0.12	(0.91)	0.02	(0.07)	1.74	(0.20)	(0.34)
**FTS**	2017	-	-	-	7.49	0.12	-	-	-	1.22	-	-
**FTS**	2018	0.11	(0.03)	(0.11)	7.82	0.22	0.16	(0.04)	(0.15)	1.42	(0.00)	(0.00)
**FTS**	2019	-	-	-	7.83	0.10	-	-	-	1.57	-	-
**FTS**	2020	0.35	(0.02)	0.04	8.13	0.07	0.62	(0.03)	0.06	1.79	0.13	0.23
**FTS**	2021	1.63	(0.03)	(0.18)	9.15	0.10	3.00	(0.05)	(0.33)	1.84	0.52	0.96
**BSI**	2011	-	-	-	8.06	0.07	-	-	-	1.80	-	-
**BSI**	2012	(2.88)	0.00	0.35	7.18	0.02	(4.17)	0.00	0.51	1.45	(0.93)	(1.34)
**BSI**	2013	-	-	-	7.34	0.01	-	-	-	2.60	-	-
**BSI**	2014	0.17	0.00	(2.08)	7.52	0.04	0.99	0.00	(11.87)	5.71	(0.57)	(3.23)
**BSI**	2015	-	-	-	7.78	0.04	-	-	-	4.99	-	-
**BSI**	2016	(1.28)	0.00	0.96	7.28	0.09	(4.19)	0.01	3.13	3.27	(0.17)	(0.55)
**BSI**	2017	-	-	-	7.76	0.09	-	-	-	5.14	-	-
**BSI**	2018	(0.72)	0.01	(0.16)	7.47	0.14	(3.88)	0.03	(0.87)	5.38	(0.31)	(1.64)
**BSI**	2019	-	-	-	7.78	0.06	-	-	-	5.28	-	-
**BSI**	2020	0.23	(0.00)	0.09	7.98	0.06	1.13	(0.02)	0.44	4.97	0.11	0.53
**BSI**	2021	1.53	(0.00)	(0.21)	8.70	0.07	5.23	(0.01)	(0.71)	3.41	0.49	1.66
**CTS**	2011	-	-	-	7.30	0.06	-	-	-	3.39	-	-
**CTS**	2012	(0.48)	(0.00)	(0.10)	7.00	0.09	(2.53)	(0.01)	(0.55)	5.33	(0.20)	(1.08)

**CTS**	2013	-	-	-	6.99	0.08	-	-	-	5.95	-	-
**CTS**	2014	0.03	0.03	0.08	7.04	0.07	0.11	0.13	0.33	3.90	0.05	0.18
**CTS**	2015	-	-	-	7.19	0.07	-	-	-	5.12	-	-
**CTS**	2016	(0.00)	(0.00)	0.10	7.26	0.07	(0.02)	(0.00)	0.48	4.88	0.03	0.14
**CTS**	2017	-	-	-	7.52	0.07	-	-	-	5.50	-	-
**CTS**	2018	0.39	(0.02)	0.19	7.85	0.07	-	-	-		0.19	-
**CTS**	2019	-	-	-	7.94	0.05	-	-	-	4.13	-	-
**CTS**	2020	0.84	(0.01)	0.13	8.36	0.04	3.67	(0.05)	0.57	4.37	0.34	1.48
**CTS**	2021	0.72	(0.01)	(0.39)	8.81	0.07	3.02	(0.02)	(1.64)	4.22	0.14	0.58
**VDS**	2011	-	-	-	6.82	0.14	-	-	-	4.01	-	-
**VDS**	2012	(0.92)	0.01	1.17	6.40	0.05	(3.24)	0.02	4.13	3.51	0.03	0.09
**VDS**	2013	-	-	-	6.53	0.01	-	-	-	5.16	-	-
**VDS**	2014	0.33	(0.01)	(3.60)	7.00	0.10	1.29	(0.03)	(14.28)	3.97	(0.97)	(3.87)
**VDS**	2015	-	-	-	7.13	0.01	-	-	-		-	-
**VDS**	2016	0.33	(0.00)	(2.98)	7.37	0.04	0.56	(0.01)	(5.02)	1.69	(0.78)	(1.32)
**VDS**	2017	-	-	-	7.52	0.07	-	-	-	2.20	-	-
**VDS**	2018	(0.03)	(0.00)	0.39	7.57	0.04	(0.07)	(0.00)	1.09	2.83	0.11	0.30
**VDS**	2019	-	-	-	7.74	0.02	-	-	-	1.66	-	-
**VDS**	2020	0.00	(0.00)	(1.06)	7.85	0.08	0.01	(0.00)	(2.15)	2.03	(0.32)	(0.65)
**VDS**	2021	0.25	0.00	(0.15)	8.30	0.13	0.37	0.00	(0.24)	1.52	0.04	0.06
**BVS**	2011	-	-	-	7.21	0.07	-	-	-	2.95	-	-
**BVS**	2012	0.07	(0.00)	0.13	7.34	0.05	-	-	-		0.06	-
**BVS**	2013	-	-	-	7.49	0.05	-	-	-	3.61	-	-
**BVS**	2014	(0.07)	(0.00)	(0.13)	7.54	0.07	(0.23)	(0.01)	(0.41)	3.06	(0.07)	(0.21)
**BVS**	2015	-	-	-	7.65	0.06	-	-	-	4.70	-	-
**BVS**	2016	(0.26)	0.00	0.11	7.53	0.06	(1.06)	0.01	0.47	4.16	(0.06)	(0.24)
**BVS**	2017	-	-	-	7.70	0.07	-	-	-	5.73	-	-
**BVS**	2018	0.43	(0.00)	0.25	7.98	0.04	0.74	(0.00)	0.43	1.70	0.23	0.39
**BVS**	2019	-	-	-	7.78	0.06	-	-	-	1.70	-	-
**BVS**	2020	0.49	(0.00)	(0.06)	8.14	0.05	1.18	(0.01)	(0.13)	2.40	0.16	0.38
**BVS**	2021	0.85	(0.00)	0.02	8.65	0.06	1.53	(0.00)	0.04	1.81	0.31	0.56
**VIX**	2011	-	-	-	5.87	0.03	-	-	-	-	-	-
**VIX**	2012	0.01	0.00	(1.44)	5.62	0.18	0.01	0.00	(1.54)	1.07	(0.43)	(0.46)
**VIX**	2013	-	-	-	5.76	0.13	-	-	-	1.40	-	-

**VIX**	2014	0.10	(0.01)	0.02	6.08	0.23	0.12	(0.01)	0.03	1.30	0.04	0.05
**VIX**	2015	-	-	-	6.85	0.09	-	-	-	1.40	-	-
**VIX**	2016	0.16	(0.00)	0.37	7.00	0.06	0.42	(0.01)	0.97	2.60	0.17	0.44
**VIX**	2017	-	-	-	7.19	0.07	-	-	-		-	-
**VIX**	2018	(0.05)	(0.00)	(0.69)	7.33	0.18	(0.10)	(0.01)	(1.54)	2.25	(0.22)	(0.51)
**VIX**	2019	-	-	-	7.48	0.08	-	-	-	1.92	-	-
**VIX**	2020	(0.03)	(0.00)	(0.34)	7.66	0.19	(0.13)	(0.00)	(1.31)	3.83	(0.12)	(0.44)
**VIX**	2021	0.05	(0.00)	(0.02)	8.46	0.19	0.24	(0.00)	(0.09)	4.97	0.01	0.06
**IVS**	2011	-	-	#DIV/0!	5.27		-	-	#DIV/0!	4.90	#DIV/0!	#DIV/0!
**IVS**	2012	0.06	(0.03)	#DIV/0!	5.34	0.01	0.31	(0.13)	#DIV/0!	4.77	#DIV/0!	#DIV/0!
**IVS**	2013	-	-	-	5.42	0.01	-	-	-	3.70	-	-
**IVS**	2014	0.02	(0.02)	(6.00)	5.49	0.05	0.02	(0.02)	(6.34)	1.06	(1.81)	(1.92)
**IVS**	2015	-	-	-	5.44	0.03	-	-	-	1.09	-	-
**IVS**	2016	(0.36)	(0.03)	#DIV/0!	5.85		(0.39)	(0.03)	#DIV/0!	1.08	#DIV/0!	#DIV/0!
**IVS**	2017	-	-	#DIV/0!	5.85		-	-	#DIV/0!	1.08	#DIV/0!	#DIV/0!
**IVS**	2018	(0.00)	(0.00)	#DIV/0!	5.85	0.01	(0.00)	(0.02)	#DIV/0!	5.63	#DIV/0!	#DIV/0!
**IVS**	2019	-	-	-	6.51	0.09	-	-	-	4.55	-	-
**IVS**	2020	0.00	0.01	2.22	6.53	0.01	0.00	0.04	7.08	3.19	0.68	2.16
**IVS**	2021	0.18	(0.01)	(2.34)	6.74	0.05	0.58	(0.02)	(7.77)	3.32	(0.65)	(2.15)
**APG**	2011	-	-	-	4.62	0.38	-	-	-		-	-
**APG**	2012	(0.01)	#DIV/0!	#DIV/0!	#NUM!	#DIV/0!	-	#DIV/0!	#DIV/0!		#DIV/0!	#DIV/0!
**APG**	2013	-	-	-	5.01	0.03	-	-	-	1.97	-	-
**APG**	2014	(0.23)	0.00	0.28	4.91	0.06	(0.77)	0.01	0.93	3.38	0.00	0.01
**APG**	2015	-	-	-	5.04	0.16	-	-	-	2.23	-	-
**APG**	2016	(0.05)	(0.01)	#DIV/0!	5.00		(0.23)	(0.03)	#DIV/0!	4.54	#DIV/0!	#DIV/0!
**APG**	2017	-	-	-	4.99	0.07	-	-	-	4.74	-	-
**APG**	2018	0.01	-	0.06	5.00	0.06	0.05	-	0.22	4.01	0.02	0.09
**APG**	2019	-	-	-	5.96	0.08	-	-	-		-	-
**APG**	2020	0.01	0.01	0.01	6.03	0.07	0.02	0.03	0.02	3.58	0.01	0.02
**APG**	2021	0.13	(0.01)	(0.13)	7.07	0.26	0.70	(0.06)	(0.70)	5.51	0.00	0.02
**HBS**	2011	-	-	-	5.96	0.00	-	-	-	5.89	-	-
**HBS**	2012	(0.02)	(0.02)	#DIV/0!	5.93		(0.13)	(0.08)	#DIV/0!	5.29	#DIV/0!	#DIV/0!
**HBS**	2013	-	-	#DIV/0!	5.92		-	-	#DIV/0!	5.27	#DIV/0!	#DIV/0!
**HBS**	2014	0.04	(0.00)	#DIV/0!	5.96	0.01	0.19	(0.00)	#DIV/0!	5.40	#DIV/0!	#DIV/0!

**HBS**	2015	-	-	-	5.97	0.00	-	-	-	4.96	-	-
**HBS**	2016	(0.05)	-	(2.00)	5.92	0.01	(0.18)	-	(6.89)	3.45	(0.62)	(2.15)
**HBS**	2017	-	-	-	5.99	0.01	-	-	-		-	-
**HBS**	2018	(0.07)	0.01	-	5.94	0.01	(0.33)	0.06	-	4.88	(0.02)	(0.10)
**HBS**	2019	-	-	-	5.96	0.02	-	-	-	3.90	-	-
**HBS**	2020	0.01	(0.01)	0.58	5.97	0.01	0.01	(0.01)	0.86	1.50	0.17	0.26
**HBS**	2021	0.00	(0.00)	(0.20)	6.00	0.02	0.02	(0.01)	(0.79)	3.95	(0.06)	(0.24)
**FPT**	2011	-	-	-	9.61	0.17	-	-	-	1.31	-	-
**FPT**	2012	(0.43)	0.04	0.02	9.56	0.17	(1.56)	0.15	0.06	3.66	(0.14)	(0.49)
**FPT**	2013	-	-	-	9.77	0.15	-	-	-	1.00	-	-
**FPT**	2014	0.40	(0.01)	0.33	10.03	0.11	0.41	(0.01)	0.33	1.01	0.24	0.25
**FPT**	2015	-	-	-	10.17	0.11	-	-	-	1.01	-	-
**FPT**	2016	0.05	(0.01)	0.20	10.30	0.10	0.05	(0.01)	0.21	1.05	0.07	0.08
**FPT**	2017	-	-	-	10.13	0.17	-	-	-	3.27	-	-
**FPT**	2018	0.13	0.01	(0.13)	10.30	0.13	0.14	0.01	(0.14)	1.09	0.01	0.01
**FPT**	2019	-	-	-	10.42	0.14	-	-	-	1.17	-	-
**FPT**	2020	0.26	(0.03)	(0.06)	10.64	0.13	0.40	(0.04)	(0.10)	1.58	0.06	0.10
**FPT**	2021	0.26	(0.01)	0.00	10.89	0.12	-	-	-		0.09	-
**CMG**	2011	-	-	-	7.33	0.07	-	-	-	1.32	-	-
**CMG**	2012	0.21	0.07	20.13	7.42	0.01	0.83	0.27	80.85	4.02	6.18	24.83
**CMG**	2013	-	-	-	7.43	0.02	-	-	-	5.14	-	-
**CMG**	2014	(0.24)	0.00	(40.55)	7.47	0.07	(0.33)	0.01	(55.34)	1.36	(12.35)	(16.85)
**CMG**	2015	-	-	-	7.69	0.07	-	-	-	4.12	-	-
**CMG**	2016	0.07	0.07	(0.11)	7.79	0.08	0.21	0.20	(0.35)	3.10	0.01	0.04
**CMG**	2017	-	-	-	8.01	0.07	-	-	-	5.79	-	-
**CMG**	2018	0.32	(0.05)	(0.24)	8.22	0.07	0.54	(0.09)	(0.41)	1.69	0.02	0.04
**CMG**	2019	-	-	-	8.44	0.06	-	-	-	1.21	-	-
**CMG**	2020	0.08	(0.02)	(0.33)	8.51	0.06	0.10	(0.02)	(0.39)	1.20	(0.08)	(0.09)
**CMG**	2021	0.18	(0.01)	(0.07)	8.74	0.06	0.51	(0.01)	(0.21)	2.85	0.04	0.12
**SAM**	2011	-	-	-	7.88	0.07	-	-	-	1.85	-	-
**SAM**	2012	(0.00)	0.00	(1.05)	7.92	0.05	(0.01)	0.01	(2.13)	2.03	(0.32)	(0.64)
**SAM**	2013	-	-	-	8.00	0.05	-	-	-	2.52	-	-
**SAM**	2014	0.11	0.01	0.48	8.08	0.03	0.27	0.03	1.15	2.37	0.19	0.45
**SAM**	2015	-	-	-	8.18	0.02	-	-	-	3.22	-	-

**SAM**	2016	0.04	0.06	2.43	8.21	0.01	0.14	0.20	7.96	3.28	0.77	2.53
**SAM**	2017	-	-	-	8.37	0.03	-	-	-	2.96	-	-
**SAM**	2018	0.17	(0.03)	0.40	8.53	0.03	0.47	(0.09)	1.11	2.82	0.17	0.48
**SAM**	2019	-	-	-	8.56	0.03	-	-	-	2.57	-	-
**SAM**	2020	(0.17)	(0.02)	(1.50)	8.64	0.02	(0.44)	(0.05)	(3.80)	2.54	(0.52)	(1.32)
**SAM**	2021	0.01	(0.03)	(0.22)	8.93	0.03	0.02	(0.09)	(0.60)	2.74	(0.08)	(0.21)
**ST8**	2011	-	-	-	5.79	0.15	-	-	-	2.42	-	-
**ST8**	2012	(0.05)	(0.22)	(0.46)	5.74	0.21	-	-	-		(0.23)	-
**ST8**	2013	-	-	-	5.87	0.19	-	-	-	2.08	-	-
**ST8**	2014	(0.04)	(0.07)	0.10	5.96	0.20	(0.10)	(0.17)	0.22	2.27	(0.01)	(0.02)
**ST8**	2015	-	-	-	6.09	0.18	-	-	-	2.61	-	-
**ST8**	2016	0.03	0.03	0.25	6.01	0.20	0.08	0.07	0.67	2.66	0.10	0.26
**ST8**	2017	-	-	-	6.15	0.08	-	-	-	2.64	-	-
**ST8**	2018	(0.03)	(0.05)	(1.86)	6.11	0.12	(0.07)	(0.12)	(4.74)	2.55	(0.59)	(1.50)
**ST8**	2019	-	-	-	6.16	0.06	-	-	-	2.48	-	-
**ST8**	2020	0.26	(0.03)	2.27	6.28	0.04	0.44	(0.05)	3.94	1.74	0.77	1.33
**ST8**	2021	(0.19)	0.01	(1.23)	6.16	0.04	(0.87)	0.03	(5.80)	4.71	(0.44)	(2.05)
**POT**	2011	-	-	-	6.47	0.02	-	-	-	3.98	-	-
**POT**	2012	(0.04)	0.02	(2.06)	6.45	0.02	(0.13)	0.05	(7.04)	3.42	(0.63)	(2.16)
**POT**	2013	-	-	-	6.42	0.01	-	-	-	2.64	-	-
**POT**	2014	0.74	(0.01)	(0.54)	6.61	0.02	1.50	(0.02)	(1.09)	2.03	0.10	0.20
**POT**	2015	-	-	-	7.26	0.03	-	-	-	3.10	-	-
**POT**	2016	(1.19)	0.03	(0.39)	6.97	0.03	(3.86)	0.09	(1.27)	3.25	(0.53)	(1.74)
**POT**	2017	-	-	-	7.52	0.02	-	-	-	2.36	-	-
**POT**	2018	1.56	(0.01)	(1.21)	7.76	0.02	3.48	(0.02)	(2.70)	2.24	0.19	0.43
**POT**	2019	-	-	-	7.74	0.01	-	-	-	2.18	-	-
**POT**	2020	(0.69)	0.01	1.50	7.65	0.01	(1.55)	0.02	3.36	2.24	0.21	0.47
**POT**	2021	0.66	(0.03)	0.43	7.75	0.01	2.11	(0.10)	1.36	3.18	0.36	1.13
**ELC**	2011	-	-	-	7.04	0.11	-	-	-	3.07	-	-
**ELC**	2012	(0.53)	0.05	(0.20)	6.85	0.13	(1.41)	0.14	(0.53)	2.63	(0.23)	(0.62)
**ELC**	2013	-	-	-	6.87	0.04	-	-	-	2.67	-	-
**ELC**	2014	0.08	0.03	(0.82)	6.99	0.06	0.20	0.07	(2.04)	2.49	(0.21)	(0.52)
**ELC**	2015	-	-	-	7.04	0.08	-	-	-	2.15	-	-
**ELC**	2016	(0.10)	(0.03)	0.04	7.06	0.10	(0.23)	(0.06)	0.10	2.28	(0.03)	(0.07)

**ELC**	2017	-	-	-	6.96	0.05	-	-	-	2.41	-	-
**ELC**	2018	0.00	(0.02)	5.91	6.96	0.01	0.01	(0.06)	20.02	3.39	1.78	6.04
**ELC**	2019	-	-	-	7.25	0.03	-	-	-	2.86	-	-
**ELC**	2020	0.04	(0.01)	0.37	7.31	0.03	0.12	(0.04)	1.10	3.00	0.12	0.36
**ELC**	2021	(0.44)	(0.04)	(1.03)	7.04	0.05	(1.06)	(0.08)	(2.49)	2.42	(0.48)	(1.16)
**SMT**	2011	-	-	-	3.81	0.02	-	-	-	3.92	-	-
**SMT**	2012	0.08	(0.08)	(10.50)	4.03	0.11	0.28	(0.27)	(36.56)	3.48	(3.17)	(11.04)
**SMT**	2013	-	-	-	4.52	0.10	-	-	-	2.35	-	-
**SMT**	2014	0.28	0.01	0.39	4.74	0.10	0.71	0.02	0.99	2.54	0.22	0.56
**SMT**	2015	#DIV/0!	#DIV/0!	#DIV/0!	#NUM!	#DIV/0!	#DIV/0!	#DIV/0!	#DIV/0!	2.92	#DIV/0!	#DIV/0!
**SMT**	2016	#DIV/0!	#DIV/0!	#DIV/0!	5.23	0.08	#DIV/0!	#DIV/0!	#DIV/0!	3.15	#DIV/0!	#DIV/0!
**SMT**	2017	-	-	-	5.30	0.07	-	-	-	2.22	-	-
**SMT**	2018	0.11	(0.02)	(0.46)	5.37	0.07	0.21	(0.05)	(0.89)	1.94	(0.11)	(0.21)
**SMT**	2019	-	-	-	5.37	0.04	-	-	-	2.17	-	-
**SMT**	2020	(0.11)	0.06	5.50	5.26	0.02	(0.33)	0.18	16.44	2.99	1.64	4.92
**SMT**	2021	0.78	0.02	7.25	5.53	0.01	2.28	0.06	21.13	2.91	2.48	7.22
**HIG**	2011	-	-	-	6.37	0.03	-	-	-	3.07	-	-
**HIG**	2012	(0.31)	0.00	(3.48)	6.25	0.05	(0.54)	0.01	(6.04)	1.74	(1.16)	(2.02)
**HIG**	2013	-	-	-	6.48	0.01	-	-	-	3.00	-	-
**HIG**	2014	(0.20)	(0.00)	(11.07)	6.37	0.01	(0.62)	(0.00)	(35.09)	3.17	(3.42)	(10.83)
**HIG**	2015	-	-	-	6.40	0.04	-	-	-	3.05	-	-
**HIG**	2016	0.01	(0.03)	3.99	6.27	0.02	-	-	-		1.20	-
**HIG**	2017	-	-	-	6.75	0.00	-	-	-	2.59	-	-
**HIG**	2018	(0.79)	0.01	8.83	6.37	0.01	(1.78)	0.02	19.98	2.26	2.39	5.41
**HIG**	2019	-	-	-	6.24	0.01	-	-	-	1.93	-	-
**HIG**	2020	0.10	(0.03)	(10.67)	6.23	0.02	0.24	(0.07)	(25.10)	2.35	(3.20)	(7.52)
**HIG**	2021	(0.01)	-	17.50	6.32	0.01	(0.01)	-	37.56	2.15	5.29	11.35
**SBD**	2011	#DIV/0!	#DIV/0!	#DIV/0!	#NUM!	#DIV/0!	#DIV/0!	#DIV/0!	#DIV/0!	2.09	#DIV/0!	#DIV/0!
**SBD**	2012	#DIV/0!	#DIV/0!	#DIV/0!	#NUM!	#DIV/0!	#DIV/0!	#DIV/0!	#DIV/0!	2.00	#DIV/0!	#DIV/0!
**SBD**	2013	#DIV/0!	#DIV/0!	#DIV/0!	#NUM!	#DIV/0!	#DIV/0!	#DIV/0!	#DIV/0!	2.57	#DIV/0!	#DIV/0!
**SBD**	2014	#DIV/0!	#DIV/0!	#DIV/0!	#NUM!	#DIV/0!	#DIV/0!	#DIV/0!	#DIV/0!	2.37	#DIV/0!	#DIV/0!
**SBD**	2015	-	-	-	6.24	0.03	-	-	-	2.00	-	-
**SBD**	2016	0.39	(0.03)	(0.77)	6.42	0.03	1.01	(0.08)	(2.01)	2.61	(0.10)	(0.27)
**SBD**	2017	-	-	-	6.56	0.04	-	-	-	2.39	-	-

**SBD**	2018	(0.44)	(0.01)	4.37	6.62	0.02	(0.98)	(0.01)	9.63	2.20	1.16	2.55
**SBD**	2019	-	-	-	6.61	0.03	-	-	-	2.28	-	-
**SBD**	2020	0.45	(0.01)	9.88	6.58	0.01	0.86	(0.03)	18.82	1.91	3.14	5.99
**SBD**	2021	0.66	(0.01)	(1.63)	6.64	0.01	0.70	(0.01)	(1.73)	1.07	(0.26)	(0.28)
**UNI**	2011	-	-	-	5.14	0.05	-	-	-	2.99	-	-
**UNI**	2012	0.10	0.36	(3.50)	5.21	0.09	0.15	0.55	(5.33)	1.52	(0.90)	(1.37)
**UNI**	2013	-	-	-	5.19	0.01	-	-	-	1.77	-	-
**UNI**	2014	(0.02)	0.02	(1.00)	5.18	0.01	(0.09)	0.07	(4.19)	4.19	(0.30)	(1.27)
**UNI**	2015	-	-	-	5.11	0.10	-	-	-	1.80	-	-
**UNI**	2016	0.01	-	3.76	5.12	0.01	0.02	-	6.80	1.81	1.14	2.07
**UNI**	2017	-	-	-	5.15	0.01	-	-	-	1.62	-	-
**UNI**	2018	(0.01)	-	#DIV/0!	5.14		(0.04)	-	#DIV/0!	3.07	#DIV/0!	#DIV/0!
**UNI**	2019	-	-	#DIV/0!	5.13		-	-	#DIV/0!	3.43	#DIV/0!	#DIV/0!
**UNI**	2020	0.07	(0.01)	#DIV/0!	5.19	0.01	0.22	(0.02)	#DIV/0!	3.16	#DIV/0!	#DIV/0!
**UNI**	2021	0.47	(0.01)	#DIV/0!	5.57		1.47	(0.02)	#DIV/0!	3.13	#DIV/0!	#DIV/0!
**CKV**	2011	-	-	-	5.24	0.03	-	-	-	2.11	-	-
**CKV**	2012	0.03	0.00	(1.13)	5.25	0.04	0.06	0.01	(2.60)	2.31	(0.33)	(0.76)
**CKV**	2013	-	-	-	5.33	0.02	-	-	-	1.85	-	-
**CKV**	2014	0.61	(0.02)	0.25	5.56	0.02	1.18	(0.04)	0.48	1.92	0.29	0.55
**CKV**	2015	-	-	-	5.33	0.02	-	-	-	2.52	-	-
**CKV**	2016	(0.11)	0.26	0.40	5.29	0.03	(0.24)	0.60	0.92	2.29	0.17	0.39
**CKV**	2017	-	-	-	5.40	0.02	-	-	-	2.37	-	-
**CKV**	2018	(0.18)	(0.02)	(1.43)	5.34	0.03	(0.44)	(0.04)	(3.54)	2.47	(0.50)	(1.24)
**CKV**	2019	-	-	-	5.24	0.04	-	-	-	2.30	-	-
**CKV**	2020	(0.24)	0.03	27.71	5.06	0.01	(0.57)	0.08	65.00	2.35	8.30	19.47
**CKV**	2021	0.19	(0.07)	(19.00)	5.14	0.01	0.47	(0.18)	(46.12)	2.43	(5.70)	(13.83)
**HPT**	2011	-	-	-	5.65	0.05	-	-	-	2.52	-	-
**HPT**	2012	0.90	(0.01)	3.63	5.92	0.02	1.54	(0.02)	6.17	1.70	1.42	2.41
**HPT**	2013	-	-	-	6.09	0.03	-	-	-	1.96	-	-
**HPT**	2014	0.20	(0.00)	(1.81)	6.14	0.03	0.43	(0.00)	(3.84)	2.12	(0.48)	(1.01)
**HPT**	2015	-	-	-	5.90	0.02	-	-	-	1.96	-	-
**HPT**	2016	0.07	(0.00)	(3.79)	5.97	0.03	0.12	(0.01)	(6.78)	1.79	(1.12)	(2.01)
**HPT**	2017	-	-	-	6.00	0.03	-	-	-	1.90	-	-
**HPT**	2018	(1.05)	0.00	(0.98)	5.75	0.06	(2.00)	0.00	(1.88)	1.91	(0.67)	(1.28)

**HPT**	2019	-	-	-	6.02	0.05	-	-	-	1.80	-	-
**HPT**	2020	(0.76)	0.07	1.77	5.82	0.05	(2.20)	0.20	5.08	2.87	0.28	0.81
**HPT**	2021	(0.62)	(0.00)	(1.77)	5.70	0.08	(1.48)	(0.00)	(4.23)	2.39	(0.76)	(1.81)
**KST**	2011	-	-	-	4.58	0.03	-	-	-	2.77	-	-
**KST**	2012	(0.12)	(0.02)	(4.83)	4.55	0.06	(0.30)	(0.05)	(12.44)	2.57	(1.51)	(3.89)
**KST**	2013	-	-	-	4.57	0.06	-	-	-	2.48	-	-
**KST**	2014	(0.15)	(0.02)	2.23	4.43	0.06	(0.45)	(0.05)	6.88	3.08	0.62	1.90
**KST**	2015	-	-	-	4.80	0.04	-	-	-	2.50	-	-
**KST**	2016	0.53	(0.01)	(0.68)	5.05	0.05	1.25	(0.02)	(1.59)	2.36	(0.02)	(0.04)
**KST**	2017	-	-	-	5.25	0.05	-	-	-		-	-
**KST**	2018	(0.68)	0.00	0.45	5.02	0.05	-	-	-		(0.11)	-
**KST**	2019	-	-	-	5.16	0.07	-	-	-	1.95	-	-
**KST**	2020	(0.22)	0.01	0.14	5.11	0.07	(0.48)	0.03	0.32	2.25	(0.03)	(0.07)
**KST**	2021	(0.89)	0.01	0.87	4.62	0.05	(2.07)	0.03	2.03	2.33	(0.05)	(0.12)
**ONE**	2011	-	-	-	4.91	0.07	-	-	-	2.12	-	-
**ONE**	2012	0.60	(0.01)	0.14	5.15	0.05	1.15	(0.01)	0.27	1.91	0.26	0.49
**ONE**	2013	-	-	-	5.04	0.06	-	-	-	1.92	-	-
**ONE**	2014	1.15	(0.01)	0.83	5.43	0.03	1.54	(0.01)	1.12	1.34	0.66	0.89
**ONE**	2015	-	-	-	5.98	0.03	-	-	-	1.37	-	-
**ONE**	2016	(1.58)	(0.00)	(0.25)	5.86	0.03	(2.37)	(0.00)	(0.37)	1.50	(0.64)	(0.97)
**ONE**	2017	-	-	-	5.94	0.03	-	-	-	1.63	-	-
**ONE**	2018	(0.16)	(0.00)	1.25	5.88	0.02	(0.31)	(0.01)	2.47	1.97	0.32	0.63
**ONE**	2019	-	-	-	5.70	0.02	-	-	-	1.31	-	-
**ONE**	2020	1.89	0.04	(0.73)	6.19	0.02	2.43	0.05	(0.94)	1.29	0.47	0.61
**ONE**	2021	0.35	(0.01)	(0.21)	6.27	0.02	0.40	(0.01)	(0.24)	1.14	0.06	0.07
**VTC**	2011	-	-	-	4.54	0.07	-	-	-		-	-
**VTC**	2012	(0.14)	0.04	5.14	4.44	0.02	(0.47)	0.14	16.97	3.30	1.52	5.01
**VTC**	2013	-	-	-	4.45	0.03	-	-	-	3.50	-	-
**VTC**	2014	0.81	(0.12)	(2.83)	4.92	0.06	2.63	(0.38)	(9.22)	3.25	(0.61)	(1.97)
**VTC**	2015	-	-	-	4.86	0.09	-	-	-	3.54	-	-
**VTC**	2016	0.67	(0.09)	0.58	5.29	0.06	2.57	(0.35)	2.21	3.84	0.38	1.47
**VTC**	2017	-	-	-	6.07	0.04	-	-	-	3.55	-	-
**VTC**	2018	0.29	(0.01)	0.88	6.16	0.03	1.07	(0.03)	3.27	3.73	0.36	1.36
**VTC**	2019	-	-	-	6.28	0.03	-	-	-		-	-

**VTC**	2020	(1.69)	0.06	(0.04)	5.95	0.04	-	-	-		(0.60)	-
**VTC**	2021	(1.01)	0.01	27.57	5.54	0.00	-	-	-		7.97	-
**VAT**	2011	-	-	#DIV/0!	3.47		-	-	#DIV/0!		#DIV/0!	#DIV/0!
**VAT**	2012	(0.82)	0.04	#DIV/0!	3.14		(2.88)	0.15	#DIV/0!	3.52	#DIV/0!	#DIV/0!
**VAT**	2013	-	-	-	3.47	0.03	-	-	-	3.26	-	-
**VAT**	2014	1.43	(0.01)	1.33	4.04	0.05	3.54	(0.03)	3.31	2.48	0.91	2.26
**VAT**	2015	-	-	-	4.70	0.06	-	-	-	2.85	-	-
**VAT**	2016	0.64	(0.01)	13.57	4.98	0.01	-	-	-		4.33	-
**VAT**	2017	#DIV/0!	#DIV/0!	#DIV/0!	#NUM!	#DIV/0!	#DIV/0!	#DIV/0!	#DIV/0!		#DIV/0!	#DIV/0!
**VAT**	2018	#DIV/0!	#DIV/0!	#DIV/0!	4.75		#DIV/0!	#DIV/0!	#DIV/0!		#DIV/0!	#DIV/0!
**VAT**	2019	#DIV/0!	#DIV/0!	#DIV/0!	#NUM!	#DIV/0!	#DIV/0!	#DIV/0!	#DIV/0!		#DIV/0!	#DIV/0!
**VAT**	2020	#DIV/0!	#DIV/0!	#DIV/0!	#NUM!	#DIV/0!	#DIV/0!	#DIV/0!	#DIV/0!	4.32	#DIV/0!	#DIV/0!
**VAT**	2021	#DIV/0!	#DIV/0!	#DIV/0!	#NUM!	#DIV/0!	#DIV/0!	#DIV/0!	#DIV/0!	1.56	#DIV/0!	#DIV/0!
**PLX**	2011	#DIV/0!	#DIV/0!	#DIV/0!	#NUM!	#DIV/0!	#DIV/0!	#DIV/0!	#DIV/0!	1.54	#DIV/0!	#DIV/0!
**PLX**	2012	#DIV/0!	#DIV/0!	#DIV/0!	10.92	0.02	#DIV/0!	#DIV/0!	#DIV/0!	2.53	#DIV/0!	#DIV/0!
**PLX**	2013	-	-	-	10.96	0.04	-	-	-	2.55	-	-
**PLX**	2014	0.42	0.01	22.48	10.92	0.01	1.09	0.02	57.97	2.58	6.95	17.93
**PLX**	2015	-	-	-	10.83	0.07	-	-	-	2.89	-	-
**PLX**	2016	(0.76)	(0.01)	(1.27)	10.90	0.12	(1.94)	(0.03)	(3.26)	2.57	(0.66)	(1.70)
**PLX**	2017	-	-	-	11.03	0.08	-	-	-	2.34	-	-
**PLX**	2018	(0.20)	0.02	0.24	10.94	0.09	(0.39)	0.04	0.48	1.98	0.01	0.02
**PLX**	2019	-	-	-	11.03	0.09	-	-	-	1.90	-	-
**PLX**	2020	0.15	0.01	5.52	11.02	0.02	0.28	0.01	10.32	1.87	1.73	3.22
**PLX**	2021	(0.24)	(0.03)	(4.53)	11.08	0.06	(0.52)	(0.06)	(9.87)	2.18	(1.47)	(3.19)
**OIL**	2011	#DIV/0!	#DIV/0!	#DIV/0!	#NUM!	#DIV/0!	#DIV/0!	#DIV/0!	#DIV/0!	2.24	#DIV/0!	#DIV/0!
**OIL**	2012	#DIV/0!	#DIV/0!	#DIV/0!	#NUM!	#DIV/0!	#DIV/0!	#DIV/0!	#DIV/0!	2.40	#DIV/0!	#DIV/0!
**OIL**	2013	#DIV/0!	#DIV/0!	#DIV/0!	#NUM!	#DIV/0!	#DIV/0!	#DIV/0!	#DIV/0!	2.56	#DIV/0!	#DIV/0!
**OIL**	2014	#DIV/0!	#DIV/0!	#DIV/0!	#NUM!	#DIV/0!	#DIV/0!	#DIV/0!	#DIV/0!	2.33	#DIV/0!	#DIV/0!
**OIL**	2015	-	-	-	9.93	0.04	-	-	-	1.41	-	-
**OIL**	2016	(0.02)	(0.01)	0.90	9.97	0.03	(0.02)	(0.01)	1.14	1.26	0.26	0.33
**OIL**	2017	#DIV/0!	#DIV/0!	#DIV/0!	#NUM!	#DIV/0!	#DIV/0!	#DIV/0!	#DIV/0!	1.34	#DIV/0!	#DIV/0!
**OIL**	2018	#DIV/0!	#DIV/0!	#DIV/0!	10.11	0.00	#DIV/0!	#DIV/0!	#DIV/0!	2.02	#DIV/0!	#DIV/0!
**OIL**	2019	-	-	-	10.18	0.02	-	-	-	2.90	-	-
**OIL**	2020	(0.35)	0.01	16.16	10.00	0.01	(1.26)	0.02	58.78	3.64	4.76	17.32

**OIL**	2021	0.34	(0.03)	(19.90)	10.21	0.03	1.64	(0.15)	(95.58)	4.80	(5.90)	(28.36)
**BSR**	2011	#DIV/0!	#DIV/0!	#DIV/0!	#NUM!	#DIV/0!	#DIV/0!	#DIV/0!	#DIV/0!	3.56	#DIV/0!	#DIV/0!
**BSR**	2012	#DIV/0!	#DIV/0!	#DIV/0!	#NUM!	#DIV/0!	#DIV/0!	#DIV/0!	#DIV/0!		#DIV/0!	#DIV/0!
**BSR**	2013	#DIV/0!	#DIV/0!	#DIV/0!	#NUM!	#DIV/0!	#DIV/0!	#DIV/0!	#DIV/0!	3.22	#DIV/0!	#DIV/0!
**BSR**	2014	#DIV/0!	#DIV/0!	#DIV/0!	#NUM!	#DIV/0!	#DIV/0!	#DIV/0!	#DIV/0!	2.26	#DIV/0!	#DIV/0!
**BSR**	2015	#DIV/0!	#DIV/0!	#DIV/0!	#NUM!	#DIV/0!	#DIV/0!	#DIV/0!	#DIV/0!		#DIV/0!	#DIV/0!
**BSR**	2016	#DIV/0!	#DIV/0!	#DIV/0!	11.05	0.08	#DIV/0!	#DIV/0!	#DIV/0!	1.90	#DIV/0!	#DIV/0!
**BSR**	2017	-	-	-	11.06	0.13	-	-	-	3.44	-	-
**BSR**	2018	(0.14)	0.05	3.89	10.88	0.00	(0.51)	0.17	14.09	3.62	1.14	4.13
**BSR**	2019	-	-	-	10.89	0.06	-	-	-	2.12	-	-
**BSR**	2020	0.23	(0.06)	(0.03)	10.93	0.05	0.39	(0.11)	(0.06)	1.71	0.05	0.08
**BSR**	2021	(0.02)	(0.10)	(0.20)	11.11	0.10	(0.03)	(0.15)	(0.30)	1.50	(0.10)	(0.15)
**PVS**	2011	-	-	-	10.07	0.08	-	-	-	1.51	-	-
**PVS**	2012	(0.68)	0.02	(0.36)	9.96	0.08	(1.12)	0.03	(0.58)	1.63	(0.35)	(0.57)
**PVS**	2013	-	-	-	10.08	0.10	-	-	-	1.89	-	-
**PVS**	2014	(0.03)	(0.04)	(0.02)	10.18	0.09	(0.12)	(0.15)	(0.06)	3.44	(0.03)	(0.11)
**PVS**	2015	-	-	-	10.18	0.08	-	-	-	4.86	-	-
**PVS**	2016	(0.05)	(0.02)	(0.05)	10.15	0.05	(0.19)	(0.06)	(0.18)	3.57	(0.04)	(0.14)
**PVS**	2017	-	-	-	10.08	0.04	-	-	-		-	-
**PVS**	2018	(0.16)	(0.04)	(0.05)	10.05	0.04	(0.31)	(0.08)	(0.10)	1.96	(0.09)	(0.17)
**PVS**	2019	-	-	-	10.17	0.04	-	-	-	1.76	-	-
**PVS**	2020	0.01	(0.00)	0.29	10.18	0.04	0.02	(0.01)	0.83	2.88	0.09	0.25
**PVS**	2021	(0.06)	0.00	(1.03)	10.12	0.41	(0.09)	0.00	(1.54)	1.49	(0.33)	(0.50)
**PVD**	2011	-	-	-	9.83	0.07	-	-	-	3.64	-	-
**PVD**	2012	(0.26)	(0.06)	(0.13)	9.86	0.09	(0.81)	(0.20)	(0.40)	3.11	(0.15)	(0.48)
**PVD**	2013	-	-	-	9.98	0.11	-	-	-	2.71	-	-
**PVD**	2014	(0.17)	(0.02)	(0.12)	10.06	0.14	(0.32)	(0.03)	(0.23)	1.88	(0.10)	(0.19)
**PVD**	2015	-	-	-	20.83	0.09	-	-	-	1.70	-	-
**PVD**	2016	(0.16)	0.03	2.83	10.05	0.01	-	-	-		0.81	-
**PVD**	2017	-	-	-	9.99	0.01	-	-	-		-	-
**PVD**	2018	(0.10)	0.01	(3.36)	9.95	0.01	-	-	-		(1.05)	-
**PVD**	2019	-	-	-	9.95	0.01	-	-	-	5.75	-	-

**PVD**	2020	(0.01)	(0.02)	(0.89)	9.95	0.01	(0.03)	(0.06)	(2.77)	3.11	(0.28)	(0.87)
**PVD**	2021	0.02	(0.02)	6.62	9.94	0.00	0.06	(0.08)	23.41	3.54	2.00	7.07
**PVB**	2011	#DIV/0!	#DIV/0!	#DIV/0!	#NUM!	#DIV/0!	#DIV/0!	#DIV/0!	#DIV/0!	3.95	#DIV/0!	#DIV/0!
**PVB**	2012	#DIV/0!	#DIV/0!	#DIV/0!	6.64	0.10	#DIV/0!	#DIV/0!	#DIV/0!	3.66	#DIV/0!	#DIV/0!
**PVB**	2013	-	-	-	6.56	0.11	-	-	-	4.48	-	-
**PVB**	2014	0.42	(0.30)	(0.12)	6.97	0.18	0.89	(0.63)	(0.25)	2.12	0.01	0.03
**PVB**	2015	-	-	-	6.55	0.23	-	-	-	1.44	-	-
**PVB**	2016	(0.08)	0.06	0.22	6.10	0.12	(0.11)	0.08	0.32	1.46	0.06	0.09
**PVB**	2017	-	-	-	6.08	0.13	-	-	-	1.61	-	-
**PVB**	2018	0.01	(0.04)	0.10	6.15	0.06	0.02	(0.06)	0.16	1.62	0.02	0.03
**PVB**	2019	-	-	-	6.75	0.06	-	-	-	1.55	-	-
**PVB**	2020	(0.49)	0.02	(0.01)	6.38	0.13	(0.77)	0.03	(0.01)	1.55	(0.17)	(0.27)
**PVB**	2021	(0.24)	0.00	59.35	6.11	0.00	(0.34)	0.00	85.65	1.44	17.86	25.77
**POS**	2011	#DIV/0!	#DIV/0!	#DIV/0!	#NUM!	#DIV/0!	#DIV/0!	#DIV/0!	#DIV/0!	3.40	#DIV/0!	#DIV/0!
**POS**	2012	#DIV/0!	#DIV/0!	#DIV/0!	#NUM!	#DIV/0!	#DIV/0!	#DIV/0!	#DIV/0!	3.48	#DIV/0!	#DIV/0!
**POS**	2013	-	-	-	7.51	0.19	-	-	-	3.62	-	-
**POS**	2014	0.34	(0.11)	(0.09)	7.95	0.19	1.23	(0.38)	(0.32)	3.65	0.06	0.22
**POS**	2015	-	-	-	7.71	0.07	-	-	-	3.43	-	-
**POS**	2016	(0.54)	(0.03)	0.27	7.45	0.04	(1.78)	(0.11)	0.90	3.30	(0.12)	(0.40)
**POS**	2017	-	-	-	7.25	0.03	-	-	-	3.17	-	-
**POS**	2018	(0.41)	0.00	1.00	6.99	0.02	(1.30)	0.00	3.18	3.18	0.16	0.50
**POS**	2019	-	-	-	7.02	0.04	-	-	-	4.65	-	-
**POS**	2020	1.35	0.01	(0.67)	7.75	0.06	6.92	0.04	(3.43)	5.13	0.28	1.46
**POS**	2021	(0.82)	0.01	1.72	7.34	0.02	(4.62)	0.05	9.72	5.64	0.23	1.29
**PVC**	2011	-	-	-	7.40	0.12	-	-	-	5.57	-	-
**PVC**	2012	(0.29)	0.00	(0.78)	7.57	0.19	(1.22)	0.02	(3.29)	4.21	(0.34)	(1.43)
**PVC**	2013	-	-	-	7.16	0.08	-	-	-	4.14	-	-
**PVC**	2014	0.40	(0.03)	(0.42)	7.80	0.18	1.63	(0.11)	(1.69)	4.05	0.01	0.04
**PVC**	2015	-	-	-	7.60	0.13	-	-	-	4.30	-	-
**PVC**	2016	0.28	0.03	14.06	7.60	0.01	1.12	0.12	57.04	4.06	4.36	17.69
**PVC**	2017	-	-	-	7.67	0.01	-	-	-	3.65	-	-
**PVC**	2018	(0.12)	(0.00)	2.48	7.58	0.01	(0.44)	(0.01)	9.16	3.69	0.71	2.61

**PVC**	2019	-	-	-	7.48	0.03	-	-	-	3.40	-	-
**PVC**	2020	0.00	(0.01)	1.18	7.42	0.02	0.00	(0.02)	3.78	3.19	0.36	1.13
**PVC**	2021	0.23	(0.02)	(0.21)	7.50	0.02	0.73	(0.06)	(0.67)	3.20	0.01	0.04
**PTV**	2011	-	-	-	5.62	0.11	-	-	-	3.10	-	-
**PTV**	2012	(1.04)	0.10	(0.16)	6.27	0.07	(3.23)	0.31	(0.50)	3.10	(0.39)	(1.21)
**PTV**	2013	-	-	-	6.93	0.03	-	-	-	2.98	-	-
**PTV**	2014	(0.10)	0.03	1.09	6.89	0.03	(0.31)	0.11	3.53	3.23	0.31	0.99
**PTV**	2015	-	-	-	6.20	0.04	-	-	-	3.41	-	-
**PTV**	2016	0.06	(0.02)	3.48	6.21	0.01	0.20	(0.05)	11.65	3.35	1.07	3.58
**PTV**	2017	-	-	-	6.05	0.01	-	-	-	3.38	-	-
**PTV**	2018	0.39	(0.02)	(10.10)	6.17	0.06	1.42	(0.07)	(36.49)	3.61	(2.92)	(10.54)
**PTV**	2019	-	-	-	6.14	0.01	-	-	-	3.45	-	-
**PTV**	2020	(0.13)	0.03	#DIV/0!	6.05		(0.42)	0.10	#DIV/0!	3.14	#DIV/0!	#DIV/0!
**PTV**	2021	(0.32)	(0.00)	#DIV/0!	5.79	0.01	(1.14)	(0.00)	#DIV/0!	3.57	#DIV/0!	#DIV/0!
**PEQ**	2011	#DIV/0!	#DIV/0!	#DIV/0!	#NUM!	#DIV/0!	#DIV/0!	#DIV/0!	#DIV/0!	4.39	#DIV/0!	#DIV/0!
**PEQ**	2012	#DIV/0!	#DIV/0!	#DIV/0!	#NUM!	#DIV/0!	#DIV/0!	#DIV/0!	#DIV/0!	3.35	#DIV/0!	#DIV/0!
**PEQ**	2013	#DIV/0!	#DIV/0!	#DIV/0!	#NUM!	#DIV/0!	#DIV/0!	#DIV/0!	#DIV/0!	3.39	#DIV/0!	#DIV/0!
**PEQ**	2014	#DIV/0!	#DIV/0!	#DIV/0!	4.41	0.07	#DIV/0!	#DIV/0!	#DIV/0!	3.57	#DIV/0!	#DIV/0!
**PEQ**	2015	-	-	-	4.58	0.09	-	-	-	3.46	-	-
**PEQ**	2016	0.12	(0.02)	(0.78)	4.80	0.12	0.44	(0.07)	(2.88)	3.70	(0.20)	(0.74)
**PEQ**	2017	-	-	-	5.05	0.12	-	-	-	3.61	-	-
**PEQ**	2018	(0.90)	0.13	(1.22)	5.04	0.23	(2.90)	0.42	(3.93)	3.22	(0.65)	(2.09)
**PEQ**	2019	-	-	-	5.03	0.17	-	-	-	3.16	-	-
**PEQ**	2020	0.16	(0.03)	0.35	5.06	0.11	0.52	(0.08)	1.13	3.20	0.16	0.50
**PEQ**	2021	0.36	(0.04)	0.12	5.28	0.10	1.14	(0.12)	0.39	3.18	0.15	0.48
**PVE**	2011	-	-	-	6.52	0.12	-	-	-	2.94	-	-
**PVE**	2012	(0.70)	0.00	4.58	6.53	0.02	(1.92)	0.01	12.64	2.76	1.13	3.13
**PVE**	2013	-	-	-	6.71	0.03	-	-	-	2.73	-	-
**PVE**	2014	(0.03)	(0.00)	(1.04)	6.77	0.04	(0.09)	(0.01)	(2.87)	2.75	(0.33)	(0.90)
**PVE**	2015	-	-	-	6.94	0.04	-	-	-	3.65	-	-
**PVE**	2016	1.47	(0.01)	0.64	7.25	0.03	5.56	(0.05)	2.42	3.78	0.72	2.71
**PVE**	2017	-	-	-	7.35	0.02	-	-	-	3.42	-	-

**PVE**	2018	0.55	(0.01)	7.81	7.38	0.01	2.57	(0.06)	36.42	4.66	2.55	11.91
**PVE**	2019	-	-	-	7.22	0.01	-	-	-	3.57	-	-
**PVE**	2020	0.99	(0.01)	(7.00)	7.19	0.01	3.48	(0.03)	(24.68)	3.53	(1.76)	(6.22)
**PVE**	2021	0.55	(0.01)	3.67	7.18	0.01	1.84	(0.02)	12.31	3.36	1.30	4.38
**DHG**	2011	-	-	-	7.60	0.25	-	-	-	3.22	-	-
**DHG**	2012	(0.04)	(0.01)	0.06	7.77	0.25	(0.09)	(0.03)	0.16	2.47	0.00	0.01
**DHG**	2013	-	-	-	8.03	0.25	-	-	-	2.52	-	-
**DHG**	2014	(0.02)	0.02	0.70	8.16	0.21	(0.07)	0.05	1.97	2.83	0.21	0.59
**DHG**	2015	-	-	-	8.12	0.21	-	-	-	2.62	-	-
**DHG**	2016	0.03	(0.04)	0.19	8.28	0.19	0.08	(0.10)	0.50	2.67	0.05	0.14
**DHG**	2017	-	-	-	8.32	0.18	-	-	-	2.80	-	-
**DHG**	2018	(0.14)	(0.02)	(0.08)	8.34	0.17	(0.43)	(0.06)	(0.26)	3.03	(0.08)	(0.25)
**DHG**	2019	-	-	-	8.33	0.17	-	-	-	3.07	-	-
**DHG**	2020	0.02	(0.03)	(0.21)	8.40	0.18	-	-	-		(0.06)	-
**DHG**	2021	(0.03)	(0.02)	(0.02)	8.44	0.19	(0.06)	(0.05)	(0.05)	2.05	(0.03)	(0.05)
**PME**	2011	#DIV/0!	#DIV/0!	#DIV/0!	#NUM!	#DIV/0!	#DIV/0!	#DIV/0!	#DIV/0!	2.53	#DIV/0!	#DIV/0!
**PME**	2012	#DIV/0!	#DIV/0!	#DIV/0!	#NUM!	#DIV/0!	#DIV/0!	#DIV/0!	#DIV/0!	3.73	#DIV/0!	#DIV/0!
**PME**	2013	#DIV/0!	#DIV/0!	#DIV/0!	#NUM!	#DIV/0!	#DIV/0!	#DIV/0!	#DIV/0!	3.28	#DIV/0!	#DIV/0!
**PME**	2014	#DIV/0!	#DIV/0!	#DIV/0!	#NUM!	#DIV/0!	#DIV/0!	#DIV/0!	#DIV/0!	3.48	#DIV/0!	#DIV/0!
**PME**	2015	-	-	-	7.23	0.17	-	-	-	2.87	-	-
**PME**	2016	#DIV/0!	#DIV/0!	#DIV/0!	#NUM!	#DIV/0!	#DIV/0!	#DIV/0!	#DIV/0!	2.31	#DIV/0!	#DIV/0!
**PME**	2017	-	-	-	7.58	0.18	-	-	-	2.44	-	-
**PME**	2018	(0.02)	(0.00)	(0.09)	7.67	0.18	(0.05)	(0.00)	(0.26)	2.71	(0.04)	(0.10)
**PME**	2019	-	-	-	7.74	0.17	-	-	-	3.51	-	-
**PME**	2020	0.08	0.28	0.04	7.85	0.16	0.23	0.87	0.13	3.07	0.14	0.41
**PME**	2021	#DIV/0!	#DIV/0!	#DIV/0!	#NUM!	#DIV/0!	#DIV/0!	#DIV/0!	#DIV/0!	3.16	#DIV/0!	#DIV/0!
**IMP**	2011	-	-	-	6.72	0.13	-	-	-	3.52	-	-
**IMP**	2012	0.04	0.06	0.09	6.76	0.12	0.15	0.22	0.37	3.88	0.06	0.24
**IMP**	2013	-	-	-	6.77	0.11	-	-	-	4.95	-	-
**IMP**	2014	0.09	(0.06)	(0.23)	6.94	0.11	0.30	(0.19)	(0.75)	3.23	(0.06)	(0.18)
**IMP**	2015	-	-	-	7.00	0.11	-	-	-	3.20	-	-
**IMP**	2016	0.03	(0.01)	(0.10)	7.05	0.11	0.10	(0.02)	(0.33)	3.23	(0.02)	(0.07)

**IMP**	2017	-	-	-	7.48	0.08	-	-	-	3.20	-	-
**IMP**	2018	(0.09)	0.03	(0.42)	7.48	0.10	(0.29)	0.08	(1.33)	3.13	(0.15)	(0.48)
**IMP**	2019	-	-	-	7.52	0.11	-	-	-	3.13	-	-
**IMP**	2020	0.03	(0.02)	(0.51)	7.65	0.12	0.08	(0.06)	(1.55)	3.03	(0.15)	(0.46)
**IMP**	2021	0.10	(0.02)	(0.05)	7.74	0.10	0.28	(0.05)	(0.16)	3.00	0.01	0.04
**TRA**	2011	-	-	-	6.73	0.15	-	-	-		-	-
**TRA**	2012	0.00	0.01	0.09	6.88	0.18	-	-	-		0.03	-
**TRA**	2013	-	-	-	6.99	0.21	-	-	-		-	-
**TRA**	2014	(0.16)	0.01	0.23	7.03	0.19	-	-	-		0.01	-
**TRA**	2015	-	-	-	7.17	0.20	-	-	-	4.43	-	-
**TRA**	2016	0.02	(0.00)	(0.04)	7.23	0.21	0.08	(0.02)	(0.21)	4.70	(0.01)	(0.04)
**TRA**	2017	-	-	-	7.32	0.21	-	-	-	4.76	-	-
**TRA**	2018	0.09	(0.04)	(7.84)	7.37	0.14	0.41	(0.19)	(36.74)	4.69	(2.35)	(11.02)
**TRA**	2019	-	-	-	7.36	0.14	-	-	-	3.33	-	-
**TRA**	2020	0.00	(0.04)	(0.50)	7.41	0.16	0.01	(0.13)	(1.67)	3.32	(0.16)	(0.54)
**TRA**	2021	(0.06)	(0.04)	(0.40)	7.44	0.19	(0.23)	(0.14)	(1.52)	3.81	(0.16)	(0.59)
**DMC**	2011	-	-	-	6.73	0.14	-	-	-	4.67	-	-
**DMC**	2012	0.04	(0.00)	0.05	6.74	0.14	0.19	(0.02)	0.28	5.22	0.03	0.15
**DMC**	2013	-	-	-	6.93	0.13	-	-	-	5.68	-	-
**DMC**	2014	(0.33)	0.01	(0.55)	6.84	0.18	(1.05)	0.04	(1.74)	3.20	(0.28)	(0.89)
**DMC**	2015	-	-	-	6.90	0.19	-	-	-	3.30	-	-
**DMC**	2016	(0.02)	(0.04)	0.18	6.99	0.19	(0.10)	(0.22)	0.90	5.03	0.03	0.16
**DMC**	2017	-	-	-	7.17	0.20	-	-	-	1.82	-	-
**DMC**	2018	(0.07)	(0.03)	(0.34)	7.29	0.20	(0.08)	(0.03)	(0.35)	1.03	(0.14)	(0.14)
**DMC**	2019	-	-	-	7.33	0.19	-	-	-	3.18	-	-
**DMC**	2020	(0.11)	(0.00)	0.27	7.29	0.15	(0.18)	(0.00)	0.43	1.60	0.04	0.07
**DMC**	2021	0.07	(0.02)	0.05	7.39	0.12	0.08	(0.02)	0.05	1.01	0.03	0.03
**OPC**	2011	-	-	-	6.15	0.13	-	-	-	4.58	-	-
**OPC**	2012	(0.06)	(0.05)	(0.01)	6.21	0.15	(0.18)	(0.16)	(0.02)	2.97	(0.04)	(0.12)
**OPC**	2013	-	-	-	6.29	0.16	-	-	-	2.98	-	-
**OPC**	2014	(0.07)	(0.02)	0.19	6.28	0.16	(0.19)	(0.07)	0.54	2.87	0.02	0.07
**OPC**	2015	-	-	-	6.38	0.16	-	-	-	2.95	-	-

**OPC**	2016	0.13	(0.05)	0.23	6.65	0.13	0.38	(0.15)	0.70	2.96	0.10	0.29
**OPC**	2017	-	-	-	6.97	0.11	-	-	-	3.14	-	-
**OPC**	2018	0.04	0.02	(0.19)	7.08	0.11	0.12	0.05	(0.61)	3.15	(0.04)	(0.13)
**OPC**	2019	-	-	-	7.07	0.11	-	-	-	2.87	-	-
**OPC**	2020	(0.09)	0.01	(0.43)	7.05	0.12	(0.27)	0.03	(1.25)	2.93	(0.16)	(0.47)
**OPC**	2021	0.04	(0.01)	(0.08)	7.12	0.13	0.15	(0.04)	(0.27)	3.33	(0.01)	(0.04)
**MKP**	2011	-	-	-	6.33	0.18	-	-	-	4.11	-	-
**MKP**	2012	(0.03)	0.00	0.08	6.36	0.18	(0.09)	0.01	0.27	3.45	0.01	0.05
**MKP**	2013	-	-	-	6.48	0.17	-	-	-	3.41	-	-
**MKP**	2014	(0.07)	(0.04)	(0.66)	6.64	0.28	(0.22)	(0.13)	(1.99)	3.02	(0.24)	(0.72)
**MKP**	2015	-	-	-	6.79	0.15	-	-	-	2.92	-	-
**MKP**	2016	(0.11)	(0.02)	0.07	7.05	0.13	(0.38)	(0.08)	0.24	3.59	(0.03)	(0.09)
**MKP**	2017	-	-	-	7.17	0.12	-	-	-	3.78	-	-
**MKP**	2018	(0.08)	(0.01)	(0.11)	7.15	0.11	(0.15)	(0.01)	(0.20)	1.78	(0.07)	(0.12)
**MKP**	2019	-	-	-	7.29	0.06	-	-	-	2.09	-	-
**MKP**	2020	0.03	0.45	1.15	7.31	0.04	0.06	1.04	2.63	2.29	0.51	1.17
**MKP**	2021	0.12	(0.03)	5.01	7.40	0.01	-	-	-		1.55	-
**DHT**	2011	-	-	-	5.72	0.07	-	-	-	1.90	-	-
**DHT**	2012	(0.34)	(0.01)	1.02	5.68	0.06	(0.64)	(0.01)	1.89	1.85	0.18	0.34
**DHT**	2013	-	-	-	5.78	0.12	-	-	-	1.69	-	-
**DHT**	2014	0.36	(0.03)	0.17	5.91	0.11	0.74	(0.07)	0.35	2.08	0.17	0.35
**DHT**	2015	-	-	-	6.08	0.12	-	-	-	3.75	-	-
**DHT**	2016	(0.02)	(0.03)	(0.43)	6.24	0.14	(0.09)	(0.11)	(1.84)	4.24	(0.15)	(0.62)
**DHT**	2017	-	-	-	6.45	0.15	-	-	-	3.94	-	-
**DHT**	2018	(0.18)	0.01	(0.01)	6.46	0.17	(0.92)	0.06	(0.03)	5.00	(0.06)	(0.32)
**DHT**	2019	-	-	-	6.60	0.15	-	-	-	5.91	-	-
**DHT**	2020	0.26	(0.01)	(0.29)	6.82	0.13	1.53	(0.05)	(1.68)	5.82	0.00	0.02
**DHT**	2021	(0.99)	0.01	(0.02)	7.12	0.07	(3.27)	0.02	(0.07)	3.32	(0.36)	(1.19)
**DCL**	2011	-	-	-	6.67	0.02	-	-	-	2.97	-	-
**DCL**	2012	(0.16)	0.01	(3.95)	6.50	0.03	-	-	-		(1.25)	-
**DCL**	2013	-	-	-	6.42	0.06	-	-	-		-	-
**DCL**	2014	(0.24)	(0.02)	0.37	6.39	0.07	(0.73)	(0.06)	1.10	2.99	0.02	0.05

**DCL**	2015	-	-	-	6.66	0.10	-	-	-	2.90	-	-
**DCL**	2016	(0.09)	(0.03)	(0.42)	6.74	0.13	(0.26)	(0.07)	(1.22)	2.91	(0.17)	(0.48)
**DCL**	2017	-	-	-	7.11	0.08	-	-	-	2.68	-	-
**DCL**	2018	0.61	0.03	10.30	7.46	0.01	1.57	0.07	26.64	2.59	3.34	8.64
**DCL**	2019	-	-	-	7.45	0.06	-	-	-	2.59	-	-
**DCL**	2020	(0.08)	(0.02)	(0.07)	7.50	0.05	(0.25)	(0.07)	(0.21)	3.01	(0.06)	(0.18)
**DCL**	2021	(0.11)	0.11	(0.58)	7.48	0.06	(0.56)	0.54	(2.91)	5.02	(0.18)	(0.90)
**DP3**	2011	#DIV/0!	#DIV/0!	#DIV/0!	#NUM!	#DIV/0!	#DIV/0!	#DIV/0!	#DIV/0!	3.75	#DIV/0!	#DIV/0!
**DP3**	2012	#DIV/0!	#DIV/0!	#DIV/0!	4.58	0.07	#DIV/0!	#DIV/0!	#DIV/0!	4.56	#DIV/0!	#DIV/0!
**DP3**	2013	-	-	-	4.99	0.10	-	-	-	3.79	-	-
**DP3**	2014	(0.03)	0.16	(0.24)	5.02	0.11	(0.08)	0.48	(0.75)	3.07	(0.03)	(0.09)
**DP3**	2015	-	-	-	5.18	0.12	-	-	-	3.09	-	-
**DP3**	2016	(0.24)	(0.06)	(0.64)	5.27	0.15	(0.78)	(0.20)	(2.11)	3.31	(0.30)	(0.99)
**DP3**	2017	-	-	-	5.37	0.22	-	-	-	2.94	-	-
**DP3**	2018	(0.15)	0.00	(1.03)	5.48	0.43	(0.43)	0.00	(3.03)	2.95	(0.36)	(1.07)
**DP3**	2019	-	-	-	5.91	0.28	-	-	-	2.68	-	-
**DP3**	2020	0.05	(0.05)	(0.55)	6.09	0.32	0.13	(0.12)	(1.40)	2.55	(0.16)	(0.41)
**DP3**	2021	(0.06)	0.14	0.31	6.10	0.26	(0.15)	0.37	0.79	2.56	0.12	0.31
**PMC**	2011	-	-	-	5.02	0.34	-	-	-	2.55	-	-
**PMC**	2012	0.05	(0.05)	(0.00)	5.13	0.35	0.13	(0.12)	(0.01)	2.48	0.00	0.01
**PMC**	2013	-	-	-	5.34	0.35	-	-	-	2.38	-	-
**PMC**	2014	(0.03)	0.00	0.02	5.46	0.34	(0.05)	0.01	0.04	1.60	(0.00)	(0.00)
**PMC**	2015	-	-	-	5.56	0.32	-	-	-	2.40	-	-
**PMC**	2016	(0.07)	0.00	(0.04)	5.69	0.31	(0.16)	0.01	(0.09)	2.36	(0.03)	(0.08)
**PMC**	2017	-	-	-	5.81	0.28	-	-	-	2.33	-	-
**PMC**	2018	(0.06)	(0.02)	(0.04)	5.87	0.26	(0.14)	(0.04)	(0.10)	2.34	(0.04)	(0.09)
**PMC**	2019	-	-	-	5.95	0.24	-	-	-	2.58	-	-
**PMC**	2020	0.02	0.03	(0.02)	6.06	0.22	0.06	0.09	(0.06)	2.63	0.01	0.03
**PMC**	2021	(0.02)	(0.03)	(0.04)	6.11	0.18	(0.05)	(0.09)	(0.12)	2.77	(0.03)	(0.08)
**APC**	2011	-	-	-	5.62	0.13	-	-	-	3.38	-	-
**APC**	2012	(0.15)	0.03	0.87	5.52	0.07	(0.37)	0.08	2.09	2.39	0.22	0.53
**APC**	2013	-	-	-	5.40	0.08	-	-	-		-	-

**APC**	2014	(0.12)	(0.06)	(0.63)	5.40	0.14	(0.29)	(0.15)	(1.48)	2.33	(0.26)	(0.60)
**APC**	2015	-	-	-	5.45	0.15	-	-	-	2.22	-	-
**APC**	2016	(0.07)	(0.02)	(0.19)	5.50	0.21	(0.16)	(0.05)	(0.42)	2.27	(0.09)	(0.20)
**APC**	2017	-	-	-	5.71	0.28	-	-	-	1.99	-	-
**APC**	2018	(0.02)	(0.09)	(0.02)	5.92	0.25	(0.04)	(0.17)	(0.04)	1.86	(0.04)	(0.08)
**APC**	2019	-	-	-	6.93	0.07	-	-	-	2.55	-	-
**APC**	2020	(0.13)	0.08	0.33	6.92	0.05	(0.47)	0.28	1.19	3.65	0.08	0.29
**APC**	2021	(0.01)	0.53	26.30	6.91	0.00	(0.02)	1.36	67.00	2.55	8.13	20.71
**VDP**	2011	-	-	-	5.93	0.08	-	-	-	2.64	-	-
**VDP**	2012	0.05	(0.04)	0.22	5.99	0.06	0.10	(0.07)	0.40	1.79	0.07	0.13
**VDP**	2013	-	-	-	5.83	0.09	-	-	-	1.94	-	-
**VDP**	2014	0.11	(0.06)	(0.21)	5.96	0.10	0.24	(0.13)	(0.45)	2.11	(0.05)	(0.10)
**VDP**	2015	#DIV/0!	#DIV/0!	#DIV/0!	#NUM!	#DIV/0!	#DIV/0!	#DIV/0!	#DIV/0!	2.88	#DIV/0!	#DIV/0!
**VDP**	2016	#DIV/0!	#DIV/0!	#DIV/0!	6.22	0.15	#DIV/0!	#DIV/0!	#DIV/0!	2.93	#DIV/0!	#DIV/0!
**VDP**	2017	-	-	-	6.25	0.14	-	-	-		-	-
**VDP**	2018	0.12	(0.03)	0.15	6.41	0.11	0.33	(0.08)	0.43	2.87	0.08	0.22
**VDP**	2019	-	-	-	6.50	0.08	-	-	-	2.44	-	-
**VDP**	2020	0.09	(0.04)	0.22	6.80	0.07	0.19	(0.08)	0.47	2.19	0.08	0.19
**VDP**	2021	0.12	(0.01)	0.20	6.94	0.07	0.26	(0.03)	0.43	2.20	0.10	0.22
**VMD**	2011	-	-	-	8.30	0.01	-	-	-	2.06	-	-
**VMD**	2012	2.36	(0.00)	20.31	8.41	0.00	4.68	(0.01)	40.36	1.99	6.98	13.88
**VMD**	2013	-	-	-	8.54	0.01	-	-	-	4.94	-	-
**VMD**	2014	1.64	(0.00)	(0.92)	8.67	0.01	6.65	(0.01)	(3.71)	4.06	0.31	1.26
**VMD**	2015	-	-	-	8.76	0.01	-	-	-	2.54	-	-
**VMD**	2016	(5.30)	0.00	(3.67)	8.95	0.01	(12.83)	0.01	(8.89)	2.42	(3.01)	(7.29)
**VMD**	2017	-	-	-	9.16	0.00	-	-	-	2.23	-	-
**VMD**	2018	0.78	0.01	3.64	9.18	0.00	1.80	0.01	8.43	2.32	1.38	3.20
**VMD**	2019	-	-	-	9.11	0.01	-	-	-	2.30	-	-
**VMD**	2020	(3.67)	0.00	(0.98)	9.02	0.01	(8.45)	0.00	(2.26)	2.30	(1.61)	(3.71)
**VMD**	2021	(8.93)	0.01	6.21	8.62	0.01	(19.09)	0.01	13.28	2.14	(1.33)	(2.83)
**NDP**	2011	-	-	-	5.06	0.13	-	-	-	2.64	-	-
**NDP**	2012	0.10	0.01	0.24	5.16	0.13	0.31	0.04	0.69	2.94	0.11	0.33

**NDP**	2013	-	-	-	5.16	0.15	-	-	-	2.98	-	-
**NDP**	2014	(0.19)	(0.10)	(0.20)	5.09	0.17	(0.52)	(0.27)	(0.54)	2.73	(0.16)	(0.44)
**NDP**	2015	-	-	-	5.14	0.17	-	-	-	2.62	-	-
**NDP**	2016	(0.06)	(0.03)	(0.17)	5.19	0.17	(0.14)	(0.06)	(0.39)	2.26	(0.08)	(0.19)
**NDP**	2017	-	-	-	5.48	0.11	-	-	-	1.87	-	-
**NDP**	2018	0.09	(0.01)	0.02	5.59	0.12	0.29	(0.05)	0.05	3.18	0.03	0.11
**NDP**	2019	-	-	-	5.57	0.10	-	-	-	3.13	-	-
**NDP**	2020	(0.01)	(0.02)	0.23	5.59	0.09	(0.04)	(0.06)	0.74	3.16	0.06	0.19
**NDP**	2021	(0.01)	(0.03)	(0.20)	5.61	0.09	(0.04)	(0.08)	(0.64)	3.16	(0.07)	(0.23)
**NDC**	2011	#DIV/0!	#DIV/0!	#DIV/0!	#NUM!	#DIV/0!	#DIV/0!	#DIV/0!	#DIV/0!	3.25	#DIV/0!	#DIV/0!
**NDC**	2012	#DIV/0!	#DIV/0!	#DIV/0!	5.00	0.32	#DIV/0!	#DIV/0!	#DIV/0!	3.23	#DIV/0!	#DIV/0!
**NDC**	2013	-	-	-	5.04	0.23	-	-	-	3.11	-	-
**NDC**	2014	(0.03)	(0.08)	2.47	5.09	0.16	(0.09)	(0.23)	7.50	3.03	0.71	2.15
**NDC**	2015	-	-	-	5.06	0.09	-	-	-	5.10	-	-
**NDC**	2016	(0.04)	(0.02)	(2.18)	5.16	0.13	(0.18)	(0.09)	(10.71)	4.90	(0.68)	(3.33)
**NDC**	2017	-	-	-	5.41	0.18	-	-	-	4.52	-	-
**NDC**	2018	(0.03)	0.07	0.26	5.56	0.19	(0.15)	0.30	1.09	4.27	0.09	0.38
**NDC**	2019	-	-	-	5.68	0.20	-	-	-	4.35	-	-
**NDC**	2020	(0.02)	(0.09)	(1.12)	5.87	0.23	(0.09)	(0.37)	(4.39)	3.93	(0.38)	(1.48)
**NDC**	2021	0.04	(0.01)	0.30	6.05	0.20	0.17	(0.04)	1.23	4.05	0.10	0.42
**UPH**	2011	#DIV/0!	#DIV/0!	#DIV/0!	#NUM!	#DIV/0!	#DIV/0!	#DIV/0!	#DIV/0!	3.84	#DIV/0!	#DIV/0!
**UPH**	2012	#DIV/0!	#DIV/0!	#DIV/0!	#NUM!	#DIV/0!	#DIV/0!	#DIV/0!	#DIV/0!	5.22	#DIV/0!	#DIV/0!
**UPH**	2013	-	-	-	5.04	0.05	-	-	-	5.37	-	-
**UPH**	2014	#DIV/0!	#DIV/0!	#DIV/0!	#NUM!	#DIV/0!	#DIV/0!	#DIV/0!	#DIV/0!	4.84	#DIV/0!	#DIV/0!
**UPH**	2015	-	-	-	5.21	0.02	-	-	-	4.68	-	-
**UPH**	2016	(0.74)	(0.02)	(4.17)	5.26	0.02	(3.28)	(0.10)	(18.48)	4.44	(1.53)	(6.80)
**UPH**	2017	-	-	-	5.98	0.02	-	-	-	4.25	-	-
**UPH**	2018	0.04	(0.03)	(2.27)	6.02	0.03	0.16	(0.12)	(9.83)	4.33	(0.68)	(2.95)
**UPH**	2019	-	-	-	5.97	0.00	-	-	-	4.26	-	-
**UPH**	2020	0.11	0.01	(22.14)	5.97	0.02	0.34	0.02	(68.85)	3.11	(6.65)	(20.69)
**UPH**	2021	(0.01)	(0.01)	-	5.92	0.02	(0.03)	(0.02)	-	3.07	(0.01)	(0.02)
**AGP**	2011	-	-	-	4.82	0.03	-	-	-	3.03	-	-

**AGP**	2012	(0.12)	0.04	(5.89)	4.85	0.07	(0.37)	0.12	(18.75)	3.18	(1.81)	(5.76)
**AGP**	2013	-	-	-	4.98	0.10	-	-	-	3.24	-	-
**AGP**	2014	0.03	(0.06)	(1.09)	5.27	0.12	0.11	(0.19)	(3.65)	3.34	(0.34)	(1.13)
**AGP**	2015	-	-	-	5.34	0.08	-	-	-	3.33	-	-
**AGP**	2016	0.61	(0.08)	(1.76)	5.66	0.10	2.88	(0.35)	(8.26)	4.70	(0.34)	(1.58)
**AGP**	2017	-	-	-	5.84	0.11	-	-	-	5.65	-	-
**AGP**	2018	0.42	0.07	(0.60)	6.14	0.09	1.86	0.32	(2.71)	4.48	(0.01)	(0.04)
**AGP**	2019	-	-	-	6.29	0.07	-	-	-	5.25	-	-
**AGP**	2020	0.20	0.01	0.44	6.40	0.06	1.02	0.03	2.24	5.03	0.21	1.05
**AGP**	2021	0.14	(0.03)	0.03	6.51	0.06	0.73	(0.17)	0.17	5.06	0.05	0.26
**CNC**	2011	#DIV/0!	#DIV/0!	#DIV/0!	#NUM!	#DIV/0!	#DIV/0!	#DIV/0!	#DIV/0!	4.34	#DIV/0!	#DIV/0!
**CNC**	2012	#DIV/0!	#DIV/0!	#DIV/0!	#NUM!	#DIV/0!	#DIV/0!	#DIV/0!	#DIV/0!	2.38	#DIV/0!	#DIV/0!
**CNC**	2013	-	-	-	5.33	0.18	-	-	-	2.65	-	-
**CNC**	2014	#DIV/0!	#DIV/0!	#DIV/0!	#NUM!	#DIV/0!	#DIV/0!	#DIV/0!	#DIV/0!	3.68	#DIV/0!	#DIV/0!
**CNC**	2015	-	-	-	5.19	0.20	-	-	-	5.53	-	-
**CNC**	2016	(0.05)	(0.05)	(0.25)	5.24	0.20	(0.17)	(0.18)	(0.85)	3.42	(0.11)	(0.38)
**CNC**	2017	-	-	-	5.26	0.19	-	-	-	3.15	-	-
**CNC**	2018	(0.07)	0.01	(0.14)	5.19	0.20	(0.21)	0.04	(0.43)	3.06	(0.06)	(0.19)
**CNC**	2019	-	-	-	5.63	0.12	-	-	-	3.15	-	-
**CNC**	2020	(0.13)	0.07	(0.08)	5.63	0.14	(0.42)	0.21	(0.27)	3.20	(0.05)	(0.16)
**CNC**	2021	(0.12)	(0.02)	(0.29)	5.63	0.18	(0.39)	(0.06)	(0.96)	3.24	(0.14)	(0.45)
**SPM**	2011	-	-	-	6.95	0.06	-	-	-	1.96	-	-
**SPM**	2012	(0.07)	(0.04)	(0.09)	7.02	0.06	-	-	-		(0.06)	-
**SPM**	2013	-	-	-	7.02	0.02	-	-	-	1.40	-	-
**SPM**	2014	(0.03)	(0.02)	(1.49)	7.06	0.04	(0.04)	(0.02)	(2.11)	1.42	(0.46)	(0.66)
**SPM**	2015	-	-	-	7.06	0.02	-	-	-	2.13	-	-
**SPM**	2016	0.05	(0.01)	5.57	7.10	0.01	0.09	(0.02)	10.52	1.89	1.70	3.21
**SPM**	2017	-	-	-	6.86	0.02	-	-	-	1.81	-	-
**SPM**	2018	0.03	(0.01)	2.16	6.88	0.01	0.06	(0.01)	3.82	1.76	0.66	1.17
**SPM**	2019	-	-	-	6.93	0.01	-	-	-	2.67	-	-
**SPM**	2020	0.03	0.01	1.48	6.96	0.01	0.07	0.02	3.70	2.49	0.46	1.15
**SPM**	2021	(0.02)	(0.00)	(6.17)	6.97	0.02	(0.04)	(0.01)	(15.35)	2.49	(1.87)	(4.66)

**DBT**	2011	-	-	-	5.68	0.07	-	-	-	2.54	-	-
**DBT**	2012	0.34	0.02	0.77	5.83	0.05	0.84	0.06	1.94	2.51	0.36	0.91
**DBT**	2013	-	-	-	5.62	0.06	-	-	-	2.44	-	-
**DBT**	2014	0.19	(0.02)	1.70	5.76	0.05	0.47	(0.06)	4.26	2.51	0.57	1.44
**DBT**	2015	-	-	-	6.11	0.07	-	-	-	2.47	-	-
**DBT**	2016	(0.60)	0.06	(0.70)	6.28	0.06	(2.06)	0.22	(2.39)	3.42	(0.41)	(1.38)
**DBT**	2017	-	-	-	6.42	0.06	-	-	-	3.34	-	-
**DBT**	2018	0.11	(0.01)	1.49	6.42	0.04	0.40	(0.03)	5.54	3.71	0.49	1.81
**DBT**	2019	-	-	-	6.57	0.04	-	-	-	3.31	-	-
**DBT**	2020	0.63	(0.03)	6.60	6.80	0.02	1.98	(0.09)	20.69	3.14	2.21	6.94
**DBT**	2021	0.30	(0.02)	(1.18)	6.67	0.02	0.91	(0.06)	(3.54)	3.01	(0.25)	(0.76)
**DDN**	2011	#DIV/0!	#DIV/0!	#DIV/0!	#NUM!	#DIV/0!	#DIV/0!	#DIV/0!	#DIV/0!	3.12	#DIV/0!	#DIV/0!
**DDN**	2012	#DIV/0!	#DIV/0!	#DIV/0!	6.64	0.02	#DIV/0!	#DIV/0!	#DIV/0!	3.01	#DIV/0!	#DIV/0!
**DDN**	2013	-	-	-	6.73	0.02	-	-	-	3.29	-	-
**DDN**	2014	(1.38)	(0.00)	(1.36)	6.78	0.02	(4.54)	(0.01)	(4.47)	3.28	(0.91)	(2.98)
**DDN**	2015	-	-	-	6.82	0.02	-	-	-	2.96	-	-
**DDN**	2016	(0.36)	0.01	(0.14)	6.81	0.02	(1.15)	0.02	(0.45)	3.15	(0.17)	(0.54)
**DDN**	2017	-	-	-	6.77	0.03	-	-	-	3.16	-	-
**DDN**	2018	(0.50)	0.00	0.13	6.78	0.02	(1.63)	0.00	0.41	3.23	(0.14)	(0.46)
**DDN**	2019	-	-	-	6.85	0.02	-	-	-	3.29	-	-
**DDN**	2020	(1.57)	0.03	0.05	6.50	0.03	(5.36)	0.11	0.18	3.41	(0.54)	(1.84)
**DDN**	2021	(0.98)	0.01	0.11	6.28	0.04	(3.96)	0.05	0.43	4.04	(0.32)	(1.28)
**PPP**	2011	-	-	-	4.29	0.04	-	-	-	3.94	-	-
**PPP**	2012	1.39	(0.02)	0.67	4.75	0.03	5.40	(0.10)	2.59	3.89	0.69	2.69
**PPP**	2013	-	-	-	4.69	0.05	-	-	-	3.45	-	-
**PPP**	2014	(1.38)	(0.12)	(1.06)	4.78	0.06	(3.89)	(0.34)	(2.98)	2.82	(0.86)	(2.41)
**PPP**	2015	-	-	-	4.76	0.05	-	-	-	2.69	-	-
**PPP**	2016	(0.38)	(0.08)	(0.87)	4.88	0.08	(0.79)	(0.16)	(1.79)	2.06	(0.43)	(0.88)
**PPP**	2017	-	-	-	5.04	0.05	-	-	-	2.23	-	-
**PPP**	2018	(0.41)	(0.04)	-	5.06	0.05	(1.26)	(0.12)	-	3.10	(0.16)	(0.49)
**PPP**	2019	-	-	-	5.06	0.09	-	-	-	3.15	-	-
**PPP**	2020	(0.15)	0.02	0.57	4.98	0.08	(0.43)	0.05	1.64	2.90	0.12	0.36

**PPP**	2021	(0.01)	(0.07)	(0.80)	5.05	0.12	(0.03)	(0.22)	(2.30)	2.88	(0.27)	(0.78)
**BCP**	2011	-	-	-	5.09	0.03	-	-	-	2.92	-	-
**BCP**	2012	0.51	(0.12)	(2.67)	5.31	0.07	1.53	(0.34)	(7.95)	2.98	(0.66)	(1.97)
**BCP**	2013	-	-	-	5.46	0.03	-	-	-	3.00	-	-
**BCP**	2014	(0.95)	0.02	(0.40)	5.26	0.05	(2.69)	0.06	(1.13)	2.84	(0.45)	(1.29)
**BCP**	2015	-	-	-	5.19	0.02	-	-	-	3.63	-	-
**BCP**	2016	(0.09)	(0.02)	(1.53)	5.18	0.03	(0.30)	(0.07)	(5.36)	3.49	(0.50)	(1.75)
**BCP**	2017	-	-	-	5.15	0.04	-	-	-	3.28	-	-
**BCP**	2018	(2.01)	0.13	(1.21)	4.54	0.15	(5.27)	0.34	(3.19)	2.63	(1.04)	(2.74)
**BCP**	2019	-	-	-	4.32	0.04	-	-	-	2.40	-	-
**BCP**	2020	0.20	(0.01)	(3.25)	4.23	0.17	0.50	(0.02)	(8.11)	2.49	(0.91)	(2.28)
**BCP**	2021	0.55	(0.01)	2.42	4.45	0.05	1.36	(0.03)	6.01	2.49	0.92	2.30
**MKV**	2011	-	-	#DIV/0!	2.94		-	-	#DIV/0!	2.55	#DIV/0!	#DIV/0!
**MKV**	2012	0.32	0.08	#DIV/0!	2.89	0.11	1.58	0.38	#DIV/0!	4.99	#DIV/0!	#DIV/0!
**MKV**	2013	-	-	-	4.22	0.03	-	-	-	4.08	-	-
**MKV**	2014	0.17	(0.04)	(2.83)	4.36	0.04	0.64	(0.15)	(10.78)	3.80	(0.81)	(3.08)
**MKV**	2015	-	-	-	4.36	0.01	-	-	-	3.77	-	-
**MKV**	2016	(0.33)	(0.20)	(14.00)	4.88	0.02	(1.19)	(0.72)	(49.89)	3.56	(4.42)	(15.75)
**MKV**	2017	-	-	#DIV/0!	4.90		-	-	#DIV/0!	3.41	#DIV/0!	#DIV/0!
**MKV**	2018	(0.12)	0.01	#DIV/0!	4.85		(0.39)	0.04	#DIV/0!	3.40	#DIV/0!	#DIV/0!
**MKV**	2019	-	-	-	4.66	0.02	-	-	-	3.31	-	-
**MKV**	2020	(0.35)	(0.05)	(7.38)	4.60	0.08	(0.54)	(0.07)	(11.42)	1.55	(2.37)	(3.67)
**MKV**	2021	(0.05)	(0.13)	(0.29)	4.69	0.08	(0.10)	(0.27)	(0.62)	2.14	(0.15)	(0.32)
**DPP**	2011	-	-	-	4.60	0.06	-	-	-	1.68	-	-
**DPP**	2012	#DIV/0!	#DIV/0!	#DIV/0!	#NUM!	#DIV/0!	#DIV/0!	#DIV/0!	#DIV/0!		#DIV/0!	#DIV/0!
**DPP**	2013	-	-	-	4.39	0.06	-	-	-	2.10	-	-
**DPP**	2014	1.00	(0.08)	1.35	4.72	0.04	2.13	(0.17)	2.86	2.12	0.74	1.57
**DPP**	2015	-	-	-	4.30	0.05	-	-	-	2.12	-	-
**DPP**	2016	(0.13)	(0.00)	-	4.25	0.06	(0.29)	(0.00)	-	2.19	(0.05)	(0.11)
**DPP**	2017	-	-	-	4.22	0.07	-	-	-	2.34	-	-
**DPP**	2018	(0.03)	(0.08)	(0.30)	4.52	0.07	(0.06)	(0.16)	(0.63)	2.09	(0.13)	(0.26)
**DPP**	2019	-	-	-	4.49	0.08	-	-	-	2.24	-	-

**DPP**	2020	(0.16)	(0.01)	(0.14)	4.44	0.09	(0.37)	(0.03)	(0.34)	2.37	(0.10)	(0.25)
**DPP**	2021	0.16	(0.03)	(0.44)	4.53	0.10	0.35	(0.06)	(1.00)	2.24	(0.09)	(0.20)
**DNM**	2011	-	-	-	4.94	0.03	-	-	-	2.29	-	-
**DNM**	2012	(0.32)	(0.03)	(8.71)	5.00	0.11	(0.67)	(0.07)	(18.41)	2.11	(2.76)	(5.83)
**DNM**	2013	#DIV/0!	#DIV/0!	#DIV/0!	#NUM!	#DIV/0!	#DIV/0!	#DIV/0!	#DIV/0!	2.10	#DIV/0!	#DIV/0!
**DNM**	2014	#DIV/0!	#DIV/0!	#DIV/0!	4.99	0.18	#DIV/0!	#DIV/0!	#DIV/0!	1.23	#DIV/0!	#DIV/0!
**DNM**	2015	-	-	-	4.91	0.14	-	-	-	1.64	-	-
**DNM**	2016	(0.04)	(0.01)	0.11	4.91	0.14	(0.06)	(0.01)	0.17	1.65	0.02	0.03
**DNM**	2017	-	-	-	4.90	0.10	-	-	-	1.78	-	-
**DNM**	2018	0.21	(0.04)	4.34	4.99	0.03	0.35	(0.07)	7.31	1.69	1.37	2.31
**DNM**	2019	-	-	-	5.36	0.05	-	-	-	1.65	-	-
**DNM**	2020	1.76	0.12	(1.24)	6.24	0.09	2.71	0.19	(1.91)	1.54	0.30	0.46
**DNM**	2021	(0.53)	(0.07)	(0.33)	6.25	0.07	(0.89)	(0.12)	(0.55)	1.66	(0.32)	(0.52)
**DBM**	2011	#DIV/0!	#DIV/0!	#DIV/0!	#NUM!	#DIV/0!	#DIV/0!	#DIV/0!	#DIV/0!	1.84	#DIV/0!	#DIV/0!
**DBM**	2012	#DIV/0!	#DIV/0!	#DIV/0!	4.47	0.13	#DIV/0!	#DIV/0!	#DIV/0!	1.65	#DIV/0!	#DIV/0!
**DBM**	2013	-	-	-	4.43	0.15	-	-	-	1.73	-	-
**DBM**	2014	(0.10)	(0.00)	0.31	4.50	0.14	(0.19)	(0.01)	0.58	1.88	0.06	0.10
**DBM**	2015	-	-	-	4.56	0.09	-	-	-	1.79	-	-
**DBM**	2016	(0.39)	0.01	0.40	4.45	0.09	(0.68)	0.01	0.70	1.74	(0.02)	(0.03)
**DBM**	2017	-	-	-	4.63	0.09	-	-	-	1.77	-	-
**DBM**	2018	(0.15)	(0.01)	0.24	4.55	0.08	(0.20)	(0.01)	0.32	1.36	0.02	0.02
**DBM**	2019	-	-	-	4.56	0.09	-	-	-	2.41	-	-
**DBM**	2020	(0.05)	(0.01)	0.36	4.58	0.08	(0.13)	(0.03)	0.94	2.61	0.09	0.23
**DBM**	2021	(0.12)	(0.00)	(0.05)	4.60	0.10	(0.29)	(0.00)	(0.12)	2.38	(0.06)	(0.14)
**EID**	2011	#DIV/0!	#DIV/0!	#DIV/0!	#NUM!	#DIV/0!	#DIV/0!	#DIV/0!	#DIV/0!	2.35	#DIV/0!	#DIV/0!
**EID**	2012	#DIV/0!	#DIV/0!	#DIV/0!	5.69	0.12	#DIV/0!	#DIV/0!	#DIV/0!	2.35	#DIV/0!	#DIV/0!
**EID**	2013	-	-	-	5.70	0.12	-	-	-	2.35	-	-
**EID**	2014	0.16	0.00	(0.03)	5.83	0.11	0.39	0.01	(0.07)	2.41	0.05	0.12
**EID**	2015	-	-	-	5.93	0.11	-	-	-	2.55	-	-
**EID**	2016	0.11	0.01	0.21	6.05	0.11	0.33	0.03	0.63	2.99	0.11	0.32
**EID**	2017	-	-	-	6.06	0.11	-	-	-	2.59	-	-
**EID**	2018	(0.04)	(0.01)	(0.23)	6.10	0.12	(0.09)	(0.01)	(0.54)	2.35	(0.08)	(0.20)

**EID**	2019	-	-	-	6.15	0.12	-	-	-	2.43	-	-
**EID**	2020	(0.10)	(0.00)	(0.19)	6.17	0.13	(0.26)	(0.01)	(0.50)	2.66	(0.09)	(0.25)
**EID**	2021	(0.03)	0.01	0.46	6.22	0.13	(0.09)	0.02	1.27	2.79	0.13	0.36
**SED**	2011	-	-	-	5.37	0.15	-	-	-	2.56	-	-
**SED**	2012	(0.16)	-	0.09	5.36	0.15	(0.41)	-	0.24	2.51	(0.03)	(0.08)
**SED**	2013	-	-	-	5.35	0.16	-	-	-	2.50	-	-
**SED**	2014	(0.16)	0.02	0.23	5.33	0.15	(0.54)	0.07	0.80	3.49	0.02	0.07
**SED**	2015	-	-	-	5.48	0.16	-	-	-	2.77	-	-
**SED**	2016	0.06	0.00	0.26	5.61	0.16	0.15	0.01	0.59	2.25	0.10	0.23
**SED**	2017	-	-	-	5.84	0.13	-	-	-	2.03	-	-
**SED**	2018	0.03	(0.01)	0.09	5.92	0.13	0.06	(0.02)	0.20	2.11	0.04	0.08
**SED**	2019	-	-	-	6.01	0.12	-	-	-	2.27	-	-
**SED**	2020	(0.20)	0.01	(0.05)	5.98	0.13	(0.35)	0.01	(0.08)	1.74	(0.08)	(0.15)
**SED**	2021	0.28	(0.01)	0.12	6.21	0.10	0.94	(0.02)	0.40	3.31	0.14	0.45
**BED**	2011	-	-	-	4.03	0.09	-	-	-	3.68	-	-
**BED**	2012	(0.10)	(0.05)	(0.80)	4.04	0.11	(0.36)	(0.16)	(2.76)	3.45	(0.30)	(1.02)
**BED**	2013	-	-	-	4.04	0.11	-	-	-	3.47	-	-
**BED**	2014	(0.12)	(0.06)	(0.67)	3.95	0.12	(0.39)	(0.18)	(2.08)	3.13	(0.27)	(0.83)
**BED**	2015	-	-	-	3.97	0.11	-	-	-	2.86	-	-
**BED**	2016	(0.03)	(0.12)	(0.88)	3.99	0.15	(0.09)	(0.34)	(2.51)	2.87	(0.32)	(0.91)
**BED**	2017	-	-	-	3.93	0.16	-	-	-	2.97	-	-
**BED**	2018	(0.14)	(0.15)	(1.45)	4.28	0.49	(0.50)	(0.53)	(5.23)	3.62	(0.54)	(1.94)
**BED**	2019	-	-	-	3.93	0.18	-	-	-	3.58	-	-
**BED**	2020	(0.01)	(0.03)	(0.33)	3.95	0.17	(0.02)	(0.09)	(1.16)	3.49	(0.11)	(0.39)
**BED**	2021	0.01	(0.05)	0.27	3.93	0.14	0.04	(0.17)	0.87	3.23	0.07	0.22
**DAD**	2011	-	-	-	4.36	0.17	-	-	-	3.28	-	-
**DAD**	2012	0.03	0.06	(0.31)	4.47	0.20	0.08	0.17	(0.93)	3.01	(0.06)	(0.20)
**DAD**	2013	-	-	-	4.81	0.13	-	-	-	2.90	-	-
**DAD**	2014	(0.48)	0.07	(0.10)	4.57	0.15	(1.35)	0.19	(0.29)	2.80	(0.18)	(0.51)
**DAD**	2015	-	-	-	4.53	0.18	-	-	-	5.77	-	-
**DAD**	2016	0.16	(0.02)	0.35	4.60	0.17	0.68	(0.10)	1.51	4.27	0.16	0.67
**DAD**	2017	-	-	-	4.55	0.13	-	-	-	2.61	-	-

**DAD**	2018	(0.04)	0.01	(1.15)	4.54	0.17	(0.09)	0.03	(2.47)	2.15	(0.36)	(0.77)
**DAD**	2019	-	-	-	4.72	0.15	-	-	-	1.62	-	-
**DAD**	2020	(0.03)	(0.01)	0.06	4.74	0.15	(0.06)	(0.02)	0.12	2.06	0.00	0.01
**DAD**	2021	0.11	(0.03)	0.12	4.87	0.15	0.29	(0.08)	0.32	2.67	0.07	0.17
**EBS**	2011	-	-	-	5.16	0.09	-	-	-	2.32	-	-
**EBS**	2012	(0.27)	(0.07)	(0.60)	5.11	0.19	(1.07)	(0.27)	(2.40)	4.00	(0.30)	(1.20)
**EBS**	2013	-	-	-	4.98	0.07	-	-	-	3.16	-	-
**EBS**	2014	0.00	0.01	1.41	4.83	0.06	0.01	0.02	2.58	1.82	0.43	0.79
**EBS**	2015	-	-	-	5.04	0.07	-	-	-	2.57	-	-
**EBS**	2016	0.08	(0.00)	(0.78)	5.12	0.09	0.20	(0.01)	(1.91)	2.44	(0.21)	(0.51)
**EBS**	2017	-	-	-	5.04	0.10	-	-	-	2.42	-	-
**EBS**	2018	(0.02)	-	0.07	5.04	0.10	(0.06)	-	0.20	2.99	0.01	0.04
**EBS**	2019	-	-	-	5.02	0.09	-	-	-	3.66	-	-
**EBS**	2020	(0.04)	(0.01)	(0.74)	5.06	0.06	(0.22)	(0.04)	(3.95)	5.32	(0.24)	(1.29)
**EBS**	2021	0.20	0.10	(0.60)	5.26	0.08	0.90	0.47	(2.76)	4.60	(0.08)	(0.35)
**ALT**	2011	-	-	-	5.51	0.02	-	-	-	4.47	-	-
**ALT**	2012	(0.01)	(0.01)	(0.69)	5.44	0.03	(0.03)	(0.05)	(2.98)	4.32	(0.22)	(0.93)
**ALT**	2013	-	-	-	5.38	0.01	-	-	-	4.12	-	-
**ALT**	2014	0.00	(0.02)	1.00	5.40	0.01	0.01	(0.08)	3.65	3.65	0.30	1.08
**ALT**	2015	-	-	-	5.46	0.03	-	-	-	3.47	-	-
**ALT**	2016	(0.06)	0.01	0.07	5.42	0.03	(0.26)	0.05	0.28	3.97	0.00	0.01
**ALT**	2017	-	-	-	5.43	0.04	-	-	-	3.76	-	-
**ALT**	2018	0.10	(0.01)	0.13	5.53	0.03	0.24	(0.03)	0.31	2.44	0.07	0.17
**ALT**	2019	-	-	-	5.50	0.04	-	-	-	3.20	-	-
**ALT**	2020	0.23	(0.07)	3.00	5.68	0.02	0.67	(0.21)	8.75	2.92	0.96	2.81
**ALT**	2021	(0.08)	0.09	0.23	5.62	0.02	(0.16)	0.19	0.47	2.02	0.07	0.15
**STC**	2011	-	-	-	4.74	0.10	-	-	-	1.75	-	-
**STC**	2012	0.26	(0.02)	0.38	4.94	0.11	0.58	(0.04)	0.85	2.26	0.20	0.45
**STC**	2013	-	-	-	4.96	0.11	-	-	-	2.33	-	-
**STC**	2014	(0.14)	(0.03)	(0.39)	4.96	0.12	(0.76)	(0.15)	(2.08)	5.39	(0.18)	(0.95)
**STC**	2015	-	-	-	4.96	0.11	-	-	-	2.17	-	-
**STC**	2016	0.01	0.07	1.03	4.97	0.10	0.02	0.17	2.61	2.54	0.34	0.85

**STC**	2017	-	-	-	5.00	0.11	-	-	-	2.90	-	-
**STC**	2018	0.00	(0.03)	(0.80)	5.04	0.12	0.01	(0.08)	(2.42)	3.04	(0.25)	(0.76)
**STC**	2019	-	-	-	5.07	0.11	-	-	-	3.79	-	-
**STC**	2020	0.25	(0.05)	0.59	5.29	0.10	1.33	(0.24)	3.13	5.32	0.25	1.34
**STC**	2021	0.26	(0.04)	(0.27)	5.48	0.09	1.39	(0.23)	(1.47)	5.42	(0.00)	(0.02)
**BST**	2011	-	-	-	2.83	0.12	-	-	-	3.80	-	-
**BST**	2012	0.04	(0.01)	-	2.94	0.11	0.11	(0.02)	-	2.54	0.01	0.04
**BST**	2013	-	-	-	2.94	0.16	-	-	-	3.67	-	-
**BST**	2014	(0.07)	-	1.50	2.94	0.11	(0.26)	-	5.47	3.64	0.43	1.56
**BST**	2015	-	-	-	2.89	0.11	-	-	-	3.58	-	-
**BST**	2016	(0.08)	-	(0.50)	2.89	0.11	(0.22)	-	(1.44)	2.89	(0.18)	(0.52)
**BST**	2017	-	-	-	2.89	0.11	-	-	-	2.96	-	-
**BST**	2018	0.19	(0.01)	-	3.04	0.10	0.64	(0.03)	-	3.35	0.07	0.22
**BST**	2019	-	-	-	2.89	0.11	-	-	-	3.70	-	-
**BST**	2020	-	-	0.50	2.89	0.11	-	-	1.81	3.62	0.15	0.55
**BST**	2021	0.21	(0.01)	-	3.04	0.10	0.79	(0.03)	-	3.69	0.07	0.27
**SMN**	2011	#DIV/0!	#DIV/0!	#DIV/0!	#NUM!	#DIV/0!	#DIV/0!	#DIV/0!	#DIV/0!	3.66	#DIV/0!	#DIV/0!
**SMN**	2012	#DIV/0!	#DIV/0!	#DIV/0!	4.14	0.13	#DIV/0!	#DIV/0!	#DIV/0!	3.55	#DIV/0!	#DIV/0!
**SMN**	2013	-	-	-	4.33	0.11	-	-	-	3.54	-	-
**SMN**	2014	(0.26)	0.00	0.11	4.25	0.13	(0.94)	0.00	0.40	3.63	(0.06)	(0.22)
**SMN**	2015	-	-	-	4.41	0.11	-	-	-	4.38	-	-
**SMN**	2016	0.00	(0.15)	0.05	4.44	0.13	0.00	(0.15)	0.05	1.01	(0.03)	(0.03)
**SMN**	2017	-	-	-	4.50	0.12	-	-	-	2.60	-	-
**SMN**	2018	(0.09)	0.01	0.19	4.49	0.13	(0.28)	0.04	0.58	3.08	0.03	0.09
**SMN**	2019	-	-	-	4.45	0.14	-	-	-	2.58	-	-
**SMN**	2020	0.10	0.03	(0.08)	4.57	0.12	0.28	0.08	(0.23)	2.75	0.02	0.06
**SMN**	2021	0.55	(0.02)	(0.27)	4.96	0.10	1.83	(0.07)	(0.91)	3.32	0.11	0.36
**SGD**	2011	-	-	-	4.41	0.07	-	-	-	3.51	-	-
**SGD**	2012	(0.30)	0.12	(0.32)	4.25	0.16	(1.03)	0.41	(1.09)	3.43	(0.16)	(0.56)
**SGD**	2013	-	-	-	4.22	0.09	-	-	-	4.62	-	-
**SGD**	2014	0.07	-	0.33	4.28	0.08	0.16	-	0.75	2.24	0.13	0.28
**SGD**	2015	-	-	-	4.43	0.05	-	-	-	2.46	-	-

**SGD**	2016	0.06	0.02	(0.20)	4.53	0.05	0.13	0.05	(0.49)	2.45	(0.03)	(0.08)
**SGD**	2017	-	-	-	4.62	0.07	-	-	-	2.38	-	-
**SGD**	2018	0.02	-	(0.04)	4.62	0.08	0.05	-	(0.09)	2.48	(0.00)	(0.01)
**SGD**	2019	-	-	-	4.58	0.06	-	-	-	2.81	-	-
**SGD**	2020	0.10	0.10	0.53	4.69	0.05	0.37	0.38	2.08	3.90	0.23	0.89
**SGD**	2021	0.29	(0.02)	1.47	4.83	0.02	0.40	(0.03)	2.04	1.39	0.54	0.75
**LBE**	2011	-	-	-	3.18	0.13	-	-	-	2.49	-	-
**LBE**	2012	(0.16)	(0.00)	0.67	3.09	0.14	(0.25)	(0.00)	1.03	1.55	0.14	0.22
**LBE**	2013	-	-	-	3.14	0.13	-	-	-	2.39	-	-
**LBE**	2014	-	-	0.83	3.14	0.09	-	-	1.99	2.39	0.25	0.60
**LBE**	2015	-	-	-	3.09	0.09	-	-	-	2.25	-	-
**LBE**	2016	-	-	(1.00)	3.09	0.14	-	-	(2.82)	2.82	(0.30)	(0.85)
**LBE**	2017	-	-	-	3.14	0.13	-	-	-	3.57	-	-
**LBE**	2018	0.00	0.00	1.17	3.09	0.09	0.02	0.02	4.45	3.81	0.36	1.36
**LBE**	2019	-	-	-	3.04	0.10	-	-	-	4.31	-	-
**LBE**	2020	-	-	0.50	3.04	0.10	-	-	1.51	3.03	0.15	0.46
**LBE**	2021	0.45	(0.06)	-	3.40	0.07	1.32	(0.17)	-	2.92	0.14	0.42
**ECI**	2011	-	-	-	3.50	0.15	-	-	-	2.39	-	-
**ECI**	2012	(0.13)	(0.00)	0.37	3.58	0.17	(0.32)	(0.01)	0.90	2.45	0.06	0.16
**ECI**	2013	-	-	-	3.64	0.18	-	-	-	3.23	-	-
**ECI**	2014	0.03	0.00	1.00	3.61	0.11	0.07	0.00	2.89	2.89	0.31	0.90
**ECI**	2015	-	-	-	3.58	0.11	-	-	-	3.33	-	-
**ECI**	2016	0.03	(0.00)	0.50	3.61	0.11	0.05	(0.00)	0.71	1.42	0.16	0.23
**ECI**	2017	-	-	-	3.64	0.11	-	-	-	2.63	-	-
**ECI**	2018	0.06	-	0.25	3.71	0.10	0.19	-	0.86	3.43	0.10	0.33
**ECI**	2019	-	-	-	3.76	0.12	-	-	-	3.59	-	-
**ECI**	2020	0.06	-	(0.47)	3.81	0.13	0.21	-	(1.66)	3.56	(0.12)	(0.43)
**ECI**	2021	0.05	-	-	3.87	0.13	0.18	-	-	3.77	0.02	0.06
**TPH**	2011	-	-	#DIV/0!	4.11		-	-	#DIV/0!	3.41	#DIV/0!	#DIV/0!
**TPH**	2012	(0.58)	0.30	#DIV/0!	3.85	0.04	(2.23)	1.13	#DIV/0!	3.82	#DIV/0!	#DIV/0!
**TPH**	2013	-	-	-	3.66	0.05	-	-	-	3.94	-	-
**TPH**	2014	(0.07)	(0.03)	-	3.66	0.05	(0.32)	(0.11)	-	4.48	(0.03)	(0.15)

**TPH**	2015	-	-	-	3.66	0.05	-	-	-	3.84	-	-
**TPH**	2016	0.09	0.02	1.00	3.71	0.02	0.29	0.06	3.38	3.38	0.34	1.15
**TPH**	2017	-	-	-	3.69	0.05	-	-	-	2.81	-	-
**TPH**	2018	0.04	0.02	1.00	3.71	0.05	0.14	0.07	3.22	3.22	0.32	1.05
**TPH**	2019	-	-	-	3.74	0.05	-	-	-	2.77	-	-
**TPH**	2020	0.05	(0.03)	0.50	3.85	0.04	0.28	(0.17)	2.60	5.20	0.16	0.83
**TPH**	2021	-	0.02	(0.50)	3.87	0.04	-	0.07	(2.37)	4.73	(0.15)	(0.69)
